# New fruit waste-derived activated carbons of high adsorption performance towards metal, metalloid, and polymer species in multicomponent systems

**DOI:** 10.1038/s41598-025-85409-0

**Published:** 2025-01-07

**Authors:** Sylwia Kukowska, Piotr Nowicki, Katarzyna Szewczuk-Karpisz

**Affiliations:** 1 Institute of Agrophysics, Polish Academy of Sciences, Doświadczalna 4, Lublin, 20-290 Poland; 2 Department of Applied Chemistry, Faculty of Chemistry, Adam Mickiewicz University in Poznań, Uniwersytetu Poznańskiego 8, Poznań, 61-614 Poland

**Keywords:** Direct/indirect physical activation, Conventional/microwave heating, Adsorption, Polymers, Pollutants, Waste management, Environmental sciences, Natural hazards, Chemistry, Engineering

## Abstract

The main aim of the study was to develop new fruit waste-derived activated carbons of high adsorption performance towards metals, metalloids, and polymers by the use of carbon dioxide (CO_2_)-consuming, microwave-assisted activation. The authors compared morphology, surface chemistry, textural parameters, and elemental composition of precursors (chokeberry seeds, black currant seeds, orange peels), as well as biochars (BCs) and activated carbons (ACs) obtained from them. The adsorption mechanisms of metals (copper, cadmium), metalloids (arsenic, selenium), and macromolecular compounds (bacterial exopolysaccharide, ionic polyacrylamides) on the surface of selected materials were investigated in one- and two-component systems. Consequently, the capacities of BCs and ACs prepared through direct/indirect physical activation, using conventional/microwave heating were determined. It was noted that microwave heating favoured surface development and thus enhanced adsorbent ability to bind ions or macromolecules. Direct biomass activation led to higher microporosity compared to indirect (two-stage) one, whilst CO_2_-consuming activation increased aromaticity and hydrophobicity of the solids. In the two-component systems, polymers could favour metal/metalloid adsorption based on complexation phenomena. However, the most efficient and environmentally safe activated carbon turned out to be the one obtained from orange peels by microwave-assisted, direct activation at 800 °C in the CO_2_ atmosphere.

**Supplementary Information:**

The online version contains supplementary material available at 10.1038/s41598-025-85409-0.

## Introduction

Bio-waste can be successfully used in various areas. In agriculture, it is applied not only as a compost, but also processed into biochars (BCs) or activated carbons (ACs) – potential conditioners improving soil quality and health. Carbon-rich materials, that can be produced from virtually any material rich in organic carbon^1^, minimize effects of toxic metals and metalloids present in the soil-water environment. Metalloids, arsenic (As(V)) and selenium (Se(IV)), as well as metals, cadmium (Cd(II)) and copper (Cu(II)), pose a serious threat to natural ecosystems. Cadmium and arsenic have no physiological functions and can damage various organs like kidneys, liver, and circulatory system^2,3^. In turn, copper and selenium are trace elements necessary for the development of plants and animals, but their excessive concentrations become dangerous. The toxic effects of copper include severe liver and kidney damage, hemolytic anemia, while excess selenium causes liver cirrhosis and pulmonary edema^4,5^. The main source of all above-mentioned hazardous elements is human activity, but their natural sources can also be indicated.

Areas contaminated with metals and metalloids are constantly increasing due to mining activities, weathering of soils and rocks, deposition of exhaust fumes, as well as use of pesticides and fertilizers^6,7^. There are 107 countries affected by arsenic contamination of groundwater. Particularly susceptible are areas with tropical climates that promote the release of arsenic from natural compounds^8^. The content of As in the Earth’s crust is approx. 1.8 ppm by weight, and thus over 90% of arsenic pollution is of natural, geogenic origin. Arsenic exists in various oxidation states in the environment, mainly as arsenite (+ 3) and arsenate (+ 5)^6^. In the sediments of the Yono River, the arsenic concentration was as high as 55.2 mg/L. At the same time, the waters of this river contained As concentrations lower than 10 µg/L^9^. In Taiwan, the As levels in groundwater ranged from 10 to 1800 µg/L, whereas in Bangladesh, from 0.5 to 4600 µg/L^8^. According to World Health Organization (WHO), a maximum acceptable concentration of arsenic in drinking water is equal to 0.01 mg/L^10^. Selenate and selenite (the soluble ones), as well as suspended particulates are the most common inorganic species of selenium in aqueous environment. Industrial processes such as burning of Se-rich fossil fuels contribute to 40% of atmospheric and aquatic Se pollution^11^. The selenium concentration in soils can reach up to 1200 mg/kg, whilst in wastewater, up to 5 g/L^11^. In ground- and surface water, the Se concentrations ranged from 0.06 to even 6000 µg/L^10,12,13^. The World Health Organization estimated the allowable daily intake of selenium at 400 µg^10,13^. Copper occurs as sulfide, copper(I) and copper(II) oxide ores, salt minerals, as well as native Cu in natural ecosystems^14,15^. Unfortunately, its application with agrochemicals and animal feed contributed to the soil contamination. What is more, the Cu production involves multiple stages, which contribute to release not only copper, but also arsenic, cadmium, and lead-containing solid particles^14^. The Cu concentrations in pig manure reached up to 2016.7 mg/kg in Beijing^15^. WHO stated that the maximum concentration of Cu in drinking water should be up to 2 mg/L^10^. Cadmium occurs as sulfides, carbonates, and phosphorites, as well as divalent ion – Cd^2+^ in natural ecosystems. Groundwater surrounding landfills in the United States contained as much as 6000 µg/L of Cd^16^, while the Cd concentrations in leachates from municipal landfills in Europe were up to 2700 µg/L^17^. According to WHO, drinking water cannot contain more than 3 µg/L of Cd^7^.

In addition to metals and metalloids, macromolecular compounds were considered as problematic contaminants. Polyacrylamides (PAMs), that are applied as stabilizers in numerous branches of industry or flocculants in wastewater treatment^18^, can be potentially toxic to aquatic life. PAM was identified as a toxicity source in municipal wastewater effluents, which affected *Ceriodaphnia dubia*^19^ and zebrafish embryos^20^. Even low concentrations of cationic polyacrylamide (CtPAM) caused mild gill lesions in juvenile rainbow trout^21^. Therefore, it is very important to effectively remove both polyacrylamides and metal/metalloid ions from contaminated aqueous solutions. Wastewater treatment is essential for protecting surface and groundwaters. There are various, commonly used methods such as adsorption, coagulation, sedimentation, filtration, membrane technologies, oxidation, ion exchange, precipitation, electrochemical treatment, electrocoagulation, or incineration^22^. However, among these techniques, adsorption is the simplest, the most effective and, at the same time, profitable. This method does not require complicated equipment, and the adsorbents used can be applied many times due to their easy regeneration. The simultaneous presence of both contaminants in the purified system may not only cause problems and difficulties, but may bring certain benefits, e.g., increased efficiency of removal of single ions and molecules. Macromolecular compounds can interact with metals and metalloids, leading to the formation of complexes that are effectively bound on the solid surface^23^. In the soil environment, polymers such as bacterial exopolysaccharides (EPS) or polyacrylamide structurants can limit mobility and bioavailability of hazardous ions^24^. EPS is a naturally occurring polymer that protects bacterial surfaces from environmental stress and supports the formation of single- or multi-species microconsortia. EPS helps *Rhizobium leguminosarum bv. trifolii* adapt to heavy metal contamination^25^. On the other hand, the PAM conditioners are environmentally stable and serve to protect soils against erosion and improve their structure^26^.

Taking these facts into account, the main aim of the study was to develop new fruit waste-derived activated carbon via pyrolysis and CO_2_-consuming, microwave-assisted activation to remove metals, metalloids, and polymers from aqueous solution. The estimation of adsorption capacity of the developed material in the systems containing two types of harmful substances (metals, metalloids, polymers) simultaneously was of high importance. The authors described textural, surface chemistry, and sorption characteristics of fruit waste (chokeberry seeds, black currant seeds, orange peels) as well as biochars and activated carbons prepared from it. The precursors and products were characterized using various analytical methods, i.e., low-temperature nitrogen (N_2_) adsorption/desorption, Fourier transform infrared spectroscopy (FTIR), X-ray photoelectron spectroscopy (XPS), potentiometric titration, elemental analysis (CHNS), scanning electron microscopy, etc. Their adsorption ability was determined towards Cu(II), Cd(II), As(V) and Se(IV) ions as well as polymers (ionic polyacrylamide, bacterial polysaccharide), and, based on the obtained results, the most promising adsorbent was selected for further experiments. The adsorption mechanisms of ions and macromolecules on the given material were investigated in detail, and experimental data were fitted to various theoretical models. To estimate strength of toxic metal or metalloid binding, desorption studies were performed.

The production of activated carbons from fruit waste using a one-step method involving microwaves and carbon dioxide is relatively new. It is well-known that it offers significant advantages over conventional methods, i.e., better textural parameters, mainly microporosity, and thus greater adsorption capacity of the prepared material. But, only few researchers used fruit waste to produce this type of carbonaceous materials as potential adsorbents of polymers, metals, or metalloids. So far, microwave- and CO_2_-assisted activation of orange peels was perform only to produce adsorbent of Cu(II)^27^ and Congo Red dye^28^. Using fruit waste in this way helps solve the problem of its management. The waste stream from processing of fruits and vegetables alone accounts for approximately 25–30% of total production^29^. This waste typically includes pomace, peels, rinds, and seeds. Orange processing is one of the sectors that generate the most waste – global orange production is estimated at roughly 60 million tonnes per year, with an annual yield of orange peel totaling 32 million tonnes^30^. The production of activated carbons from peels or seeds is thus consistent with the trends of the circular economy and could be treat as a new strategy. The only disadvantage could be gases emission during such a biochar/activated carbon production. Low levels of ethane (CH_4_), hydrogen (H_2_), and ethane (C_2_H_6_) with traces of acetaldehyde (CH_3_CHO) and nitrogen oxides (NO and NO_2_) were detected during this process, which was connected with thermal degradation of hemicellulose, cellulose and lignin^31^. Gas emissions during the production of carbonaceous materials in a microwave furnace are similar to those of conventional methods. The generated gases can be used as a source of energy or chemicals after separation and appropriate purification on filters. There are also many methods available to remove harmful gases: (1) adsorption by activated carbons, zeolites^32,33^, (2) thermal and catalytic oxidation^34^, (3) pressure swing adsorption^35^, (4) biofiltration with microorganisms^36^, (5) criogenic condensation^37^, (6) plasma treatment^38^, (7) membrane separation^39^. The method should be selected depending on the type of gaseous pollutants. In many cases, a combination of above mentioned techniques, which is suitable for the mixture of various gases, is required. The produced gases can be burnt as well, under controlled conditions, as is done during the industrial production of activated carbons. This significantly reduces the environmental footprint of the process.

## Materials and methods

### Materials

#### Biomass

Three types of biomass (B): chokeberry (*Aronia melanocarpa*) seeds (AB), orange (*Citrus sinensis*) peels (OB) and black currant (*Ribes nigrum*) seeds (RB), were used to prepare biochars (BCs) and activated carbons (ACs). Before pyrolysis/activation, they were dried at 110 °C in a laboratory dryer. Chokeberry and black currant seeds were dried without prior grinding, while orange peels were cut into 3–5 mm pieces. Chokeberry and black currant seeds were delivered by GAMA Zbigniew Olejnicki, P.P.H.U, whereas orange peels, by the Skworcu company.

#### Ions/herbicides

Cadmium(II) chloride (CdCl_2_, CAS 10108-64-2, Acros Organics) and sodium arsenate dibasic heptahydrate (Na_2_HAsO_4_·7H_2_O, CAS 10048-95-0, Sigma Aldrich), copper(II) chloride (CuCl_2_, CAS 7447-39-4, Chempur), and sodium selenite (Na_2_O_3_Se, CAS 10102-18-8, Glentham Life Sciences) were used as a source of metals (copper – Cu(II), cadmium – Cd(II)) and metalloids (arsenic – As(V), selenium – Se(IV)) ions in the study. In turn, diuron (DCMU, C_9_H_10_Cl_2_N_2_O, CAS 330-54-1, Aldrich Chemistry) and glyphosate (GLY, C_3_H_8_NO_5_P, CAS 1071-83-6, Sigma Aldrich) were used as examples of herbicides commonly applied in agriculture. The concentration of stock solutions of Cd(II), Cu(II), As(V), and Se(IV) ions was 1000 mg/L, whereas that of diuron and glyphosate, 100 mg/L. The solution of DCMU was prepared in methanol (CH_3_OH, CAS 67-56-1, Chemsolute) due to its limited solubility in water.

#### Polymers

Both natural and synthetic polymers were used in the experiments, i.e., (1) exopolysaccharide (EPS) synthesized by soil bacteria *Rhizobium leguminosarum* bv. *trifolii*, (2) cationic polyacrylamide (CtPAM), (3) anionic polyacrylamide (AnPAM). EPS was isolated courtesy of the scientists from the Institute of Biological Sciences, Maria Curie-Skłodowska University in Lublin according to the procedure described elsewhere (Szewczuk-Karpisz et al., 2022). Anionic (ID: *AN945*) and cationic (ID: *FO4350SH*) polyacrylamide were synthesized and delivered by SNF Floerger. The average molecular weight of AnPAM was 6.8, whilst that of CtPAM, 13 kDa. Both ionic polyacrylamides contained specific moieties, i.e., CtPAM – 25% of the quaternary amine groups, and AnPAM – 40% of carboxylic ones^40^. The polymer stock solutions was of concentration equal to 500 mg/L. The structure of the applied herbicides and macromolecules are presented in Table S1.

#### Others

Calcium chloride (CaCl_2_, CAS 10043-52-4, Chempur) with the concentration of 0.001 mol/dm^3^ was applied as supporting electrolyte in all examined solutions. During adsorption study, the pH value of the examined suspensions was adjusted with hydrochloric acid (HCl, CAS 7647-01-0, Chempur) and sodium hydroxide (NaOH, CAS 1310-73-2, Chempur). Standard solution of hyamine 1622 (0.004 mol/L, CAS 121-54-0, POCH) was used to determine concentration of AnPAM in the examined solutions. In turn, borate buffer (Na_2_B_4_O_7_·10H_2_O, CAS 1303 − 964, Chempur), 9-fluorenylmethylchloromethane (FMOC-Cl, CAS 28920-43-6, Glentham Life Sciences), acetonitrile (C_2_H_3_N, CAS 75-05-8, Chemsolute), and dichloromethane (CH_2_Cl_2_, CAS 75-09-2, POCh) were applied during determination of glyphosate concentration in the tested aqueous samples (for herbicide derivatization).

### Methods

#### Biochar and activated carbon preparation

To obtain biochars, the pyrolysis of dried biomass was performed at 400 °C in a conventional laboratory single-zone resistance furnace (PRW75/LM, Czylok), equipped with a 75 mm diameter quartz tubular reactor or in a muffle microwave furnace (Phoenix, CEM Corporation). The pyrolysis was carried out in an atmosphere of inert gas – technical nitrogen (Linde Gaz Polska), with a flow of 200 mL/min. About 15 g of precursors were placed in nickel boats or quartz crucibles (in case of conventional or microwave furnace, respectively), and then subjected to two-stage thermal treatment: (1) heating with a temperature gradient of 5 °C/min until reaching the final pyrolysis temperature, (2) annealing the sample at 400 °C for a period of 45 min. Then, the pyrolysis products were cooling to the room temperature under the flow of inert gas. ACs were produced in two ways: (1) by activation of the obtained biochars or (2) by direct activation of biomass, in an appropriate type of furnace. About 10 g of biochars or precursors were placed in nickel boats (conventional heating) or quartz crucibles (microwave heating), and then put in the appropriate furnace preheated to a temperature of 700–800 °C for 45 min in the carbon dioxide (Linde Gaz Polska) atmosphere, with a flow of 250 mL/min. Then, the samples were cooled in nitrogen flow. After reaching room temperature, each material was ground in a planetary ball mill (Pulverisette 6 Classic Line, Fritsch). The obtained materials were marked as: (1) BCC – the biochar obtained in conventional furnace at 400 °C, (2) BCM – the biochar obtained in microwave furnace at 400 °C, (3) ACC – the activated carbon obtained in a conventional furnace at 800 °C from biochar prepared in an identical furnace, (4) ACM – the activated carbon obtained in a microwave furnace at 800 °C from biochar prepared in an identical furnace, (5) FC700 – the activated carbon obtained in a conventional furnace at 700 °C directly from biomass, (6) FC800 – the activated carbon obtained in a conventional furnace at 800 °C directly from biomass, (7) FM700 – the activated carbon obtained in a microwave furnace at 700 °C directly from biomass, (8) FM800 – the activated carbon obtained in a microwave furnace at 800 °C directly from biomass. If the material was prepared using chokeberry seeds, the ‘A’ prefix was assigned to it, if from orange peels, ‘O’ was added, and if from black currant seeds, ‘R’ was used (see ***Abbreviations***).

Carbon compounds exhibit strong microwave absorption, thus microwave (MW) heating is the method that offers rapid, selective, consistent localized and uniform, volumetric heating of activated carbons^41^. Well-optimized microwave heating can reduce production costs of the AC production. Overall, the effects of MW power and duration varies depending on precursor, modificators, activators. Deng et al.^42^ reported that increasing of MW radiation power during one-step activation of cotton stalks with K_2_CO_3_ enhanced the yield of obtained activated carbon, but after reaching optimal conditions yield decreased due to burning and gasification. Another important factor is duration of the MW heating. The longer the MW heating time, the more energy is transferred to the precursor and significantly more active sites and pores are created. However, as with the MW radiation power, this only works under optimal conditions. With too long a time, the efficiency decreases, the pores are burned, the channels are ablated and shrink, which causes a decrease in porosity and, consequently, a decrease in the adsorption capacity of the activated carbon^42,43^. It was reported that the yield of jatropha shell-derived CO_2_-activated carbon prepared by microwave heating was twice that of conventional CO₂-activation (36.6 and 18.02 wt%), suggesting potential economic benefits for the microwave heating method. In fact, the average pore size obtained by microwave activation in the CO₂ atmosphere was slightly smaller than that of conventional activation, but contributed to a slight increase in surface area by microwave heating. Furthermore, the CO₂ activation under microwave heating significantly lowered the activation temperature, CO₂ flow rate, and activation time compared to traditional CO₂ activation, making microwave-assisted activation more cost-effective than conventional heating methods^44^.

In the performed experiments, thermochemical treatment was carried out using microwaves with a power of 1400 W and a frequency of 2.45 GHz. The pyrolysis and activation conditions were selected based on our previous studies on the synthesis of activated carbons from sawdust and brown coal^45–47^. During the production of activated carbons, a lower temperature than the one proposed in the literature was used, i.e., 800 °C was applied instead of 850 °C, which is recommended for activation with carbon dioxide. In this way, a slight reduction in environmental impact of the process was reached.

#### Characteristics of biomass, biochars, and activated carbons

Low-temperature nitrogen (N_2_) adsorption/desorption method was applied to determine textural parameters of biomass, biochars, and activated carbons (Quadrasorb SI, Quantachrome Instruments). Specific surface area (S_BET_) was calculated using Brunauer-Emmet-Teller (BET) equation:

1 $$\:\frac{1}{x\left[\left(\frac{{p}_{0}}{p}\right)-1\right]}=\frac{1}{{x}_{m}C}+\frac{C-1}{{x}_{m}C}\left(\frac{p}{{p}_{0}}\right)$$

where: *x* – the weight of nitrogen adsorbed at a given relative pressure (p/p_0_); *x*_*m*_ – the monolayer capacity, which is the volume of gas adsorbed at standard temperature and pressure (STP); and *C* – the constant.

The pore size distribution was estimated based on the method developed by Barett, Joyner and Halenda (BJH), where:

2 $$V_{{pn}} = \left( {\frac{{r_{{pn}} }}{{r_{{Kn}} + {\varDelta}\:t_{n} /2}}} \right)^{2} \left( {{\varDelta}\:V_{n} - {\varDelta}\:t_{n} \sum\limits_{{j = 1}}^{{n - 1}} {Ac_{j} } } \right)$$

where: *r*_*p*_ – the pore radius, *V*_*p*_ – the pore volume, *r*_*K*_ – the inner capillary radius, *∆t* – the thickness of adsorbed layer of nitrogen, *Ac* – the area exposed by the pore from which the physically adsorbed gas is desorbed.

Porosity parameters were estimated using the adsorption branch of the isotherm. Micropore volume (V_mic_) was calculated using the t-plot method, and total pore volume (V_t_), under relative pressure conditions p/p_0_ = 0.99. Before the measurements, the samples were outgassed at 200 °C for 20 h.

Morphology of the tested biomass, biochars, and activated carbons was observed using scanning electron microscopy (SEM) (Phenom ProX, PiK Instruments). Images were taken at accelerating voltage of 10 kV with BSD detector. Elemental analysis of the solids before and after adsorption was performed using energy dispersive X-ray spectrometer (EDS) working with SEM.

Determination of surface functional groups of biomass, biochars, and activated carbon was performed using Fourier transform infrared spectroscopy (Tensor27, Bruker Germany), from 128 scans in 4 cm^− 1^ intervals, in the range of 4000 –400 cm^− 1^. Each spectrum was corrected with a linear baseline using OMNIC (v.8.2, Thermo Scientific). The same apparatus was applied to observe changes in surface chemistry of the tested solids after adsorption of metals or metalloids.

To determine the point of zero charge (pH_pzc_) and surface charge density (σ_0_) of the investigated solid particles, potentiometric titration of their suspensions was performed using automatic burette (Titrino 702 SM, Methrom). The examined systems were titrated by 0.1 M NaOH in the pH range of 3.5–10. Surface charge density (σ_0_) was calculated using Janusz^48^ method, based on the equation:

3 $$\:{\sigma\:}_{0}=\frac{\varDelta\:V\cdot\:c\cdot\:{F}_{c}}{m\cdot\:{S}_{w}}$$

where: *ΔV* [mL] – the difference in the base volume added to the suspension and the supporting electrolyte that leads to the specific pH value, *c* [mol/L] – the base concentration, *F* – the Faraday constant, *m* [g] – the solid mass in the suspension, *S*_*BET*_ [m^2^/g] – the solid surface area.

At first, supporting electrolyte (0.001 M CaCl_2_) was titrated. Then, the suspensions of biomass/biochar/activated carbons were analyzed. The biomass suspensions were prepared by adding 1 g of the solid to 20 mL of supporting electrolyte. For biochars, 0.3 g were used, whereas for activated carbons, 0.015–0.03 g, depending on the surface charge area of AC.

The elemental composition of the biochars and activated carbons was measured using a CHNS/O analyzer (2400 Series II CHNS/O Elemental Analyzer (Perkin Elemer) and X-ray photoelectron spectroscopy (XPS) (UHV surface analysis system (SPECS)). XPS apparatus was also applied to determine the form of metals and metalloids adsorbed on the solids. The results of CHNS analyses, i.e., the contents of carbon, hydrogen, oxygen, were used to calculate the H/C, O/C and (O + N)/C molar ratios for the adsorbents. The ash content in the materials was established according to the DNS 1171:2002 standard. In turn, the amount of acidic and basic groups on the solid surface was determined by the Boehm back titration method^49^ using 0.1 M NaOH and HCl volumetric standards as the titrants and methyl orange as the indicator.

The pH of water suspensions of each material was determined by adding a portion of 0.5 g of the solid to 25 mL of distilled water and stirring for 24 h to reach equilibrium. After the time passes, the pH of the suspensions was measured using CP-401 pH-meter (Elmetron) equipped with EPS-1 glass electrode.

#### Adsorption study

Adsorption study was performed for metals, metalloids, herbicides, and polymers in both single and mixed (i.e., with two adsorbates) systems. Their adsorbed amount (Γ, mg/g) was determined based on the difference in their concentration in the solution before and after the adsorption process, using the following formula:

4 $$\:\varGamma\:=\frac{{C}_{ads}\cdot\:V}{m}$$

where: C_ads_ – the adsorbed amount of metal/metalloid ions, herbicide or polymers molecules (C_ads_ = C_0_-C_eq_) [mg/L], C_0_ – the initial adsorbate concentration [mg/L], C_eq_ – the equilibrium adsorbate concentration in the solution [mg/L], V – the system volume [L], m – the solid weight [g].

The adsorption efficiency was calculated as follows:

5 $$\:E=\:\frac{{C}_{ads}}{{C}_{0}}\cdot\:100\%$$

For Cu(II), Cd(II), polymers, and herbicides, the solid weight of 0.02 g was used (2 g/L). In turn, for As(V) and Se(IV), 0.04 g of solid was applied (4 g/L). The solid samples were added to 10 mL of the solution containing selected adsorbate and supporting electrolyte (0.001 mol/L CaCl_2_). After preparing the suspension, the pH value was adjusted to 6, and the adsorption was conducted for 24 h, under continuous shaking conditions. The pH value was monitored throughout the adsorption process, and any fluctuations were corrected to maintain a value of 6. After the process completion, the concentration of metals and metalloids was determined using atomic absorption spectrometer (ContrAA 800, Analytik Jena) working in the graphite cuvette technique. The concentration of AnPAM after adsorption was determined using hyamine 1622^50^. CtPAM and EPS concentration was measured with total organic carbon (TOC) analyzer (Multi N/C 2000, HT 1300, Analytik Jena). The concentration of diuron was determined using high performance liquid chromatography (HPLC, Dionex Ultimate 3000, Thermo Scientific), whereas the one of glyphosate, using the method developed by Waiman et al.^51^ and Specord 200 PLUS spectrophotometer (Analytik Jena).

The initial concentration of metals/metalloids, used to estimate adsorption isotherms, ranged from 10 to 250 mg/L, whereas the adsorption kinetics were assessed for their concentration of 100 mg/L. The adsorbed amount of polymers and herbicides was determined for their initial concentration of 100 mg/L and 10 mg/L, respectively. The mixed adsorption tests were conducted in following combinations: metal + metal, metalloid + metalloid, metal + herbicide, metalloid + herbicide. In the mixed systems, the concentration of metals/metalloids was 10, 100, or 250 mg/L, that of polymers, 100 mg/L, and that of herbicides, 10 or 20 mg/L.

Moreover, to reflect real-world concentrations, the adsorption test were performed for initial concentrations of Cu(II), Cd(II), As(V), and Se(IV) in the range of 20–1000 µg/L as well as the solid weight equal to 0.001–0.02 g.

#### Adsorption data modeling

For the adsorption of metals/metalloids, the equilibrium data were fitted to Langmuir, Freundlich, Langmuir-Freundlich, Temkin, Redlich-Peterson, and Dubinin-Radushkevich models. The kinetics data were fitted to the pseudo I-order (PFO), pseudo II-order (PSO), intra-particle diffusion (IPD), and Elovich equations (Table S2). The Microsoft Excel Solver was used for data modelling. Due to the fact that the adsorption of macromolecules is completely different than that of ions and small molecules (macromolecules form specific ‘loops’ and ‘tails’ on the solid surface and one polymer chain can interact with several active sites^40^, the isotherms for polymers were not measured. Their adsorbed amounts were presented only in the form of histograms.

#### Statistical analysis

All measurements were made in triplicate. The standard deviation was calculated from the obtained data.

## Results and discussion

### Characterization of biomass, biochars, and activated carbons

According to the IUPAC classification, nitrogen (N_2_) adsorption/desorption isotherms obtained for the tested materials, presented in Fig. S1, were close to type IV with H3 or H4 hysteresis loops. The H3 hysteresis, visible mainly for biomass and biochars, is usually attributed to wedge-shaped pores formed by the loose stacking of flaky particles. In turn, the H4 type, observed for all activated carbons, corresponds with slit-shaped pores resulting from internal parallel pore structure^52^. Among all precursors, black currant seeds had the largest specific surface area (Table 1). Biomass pyrolysis in a conventional furnace did not promote development of solid surface, and thus prepared biochars were characterized by low specific surface area. Microwave-assisted pyrolysis had an opposite effect and resulted in increased specific surface area of produced BC. In most cases, the obtained biochars did not contain micropores, and only the CO_2_ activation improved specific surface area and made content of micropores in the solids higher (Fig. S2). This activation was based on the following Boudouard reaction:

**Table 1:** Physicochemical parameters of biomass, biochars, and activated carbons.

	S_BET_ [m^2^/g]	V_t_ [cm^3^/g]	D [nm]	S_micro_ [m^2^/g]	V_micro_ [cm^3^/g]	S_m_/S_BET_	pH	pHpzc	Ash [% wt.]	H: C	O: C	(O + *N*): C	Acidic groups [mmol/g]	Basic groups [mmol/g]	Total groups content [mmol/g]
AB	1	0.0015	6.3432	–	–	–	4.512	5	3.12	0.13	0.66	0.71	1.004	0.224	1.228
ABCC	1	0.0015	6.365	–	–	–	6.805	6.4	10.05	0.07	0.19	0.26	0.201	0.250	0.451
ABCM	6	0.0099	6.1754	–	–	–	7.648	7.7	13.34	0.04	0.18	0.25	0.699	0.971	1.670
AACC	88	0.0651	2.9484	26	0.012	0.29	8.461	7.8	13.32	0.01	0.06	0.10	0.100	1.674	1.774
AACM	250	0.1381	2.2042	211	0.104	0.84	10.208	8.4	18.00	0.01	0.06	0.11	0.398	2.226	2.624
AFC700	123	0.0866	2.811	49	0.023	0.4	7.564	8.1	13.25	0.02	0.31	0.36	0.145	0.609	0.754
AFM700	190	0.111	2.3388	151	0.074	0.79	10.112	8.6	15.57	0.02	0.37	0.44	0.450	1.635	2.085
AFC800	88	0.06	2.7318	38	0.018	0.43	9.392	9.0	14.54	0.01	0.33	0.38	0.329	0.471	0.800
AFM800	266	0.1518	2.2858	220	0.109	0.83	10.176	9.6	16.72	0.01	0.37	0.42	0.457	1.892	2.349
OB	8	0.0092	4.718	–	–	–	4.516	4.5	1.38	0.14	1.14	1.16	1.904	0.299	2.203
OBCC	3	0.0053	7.76	–	–	–	9.283	8.4	4.47	0.06	0.35	0.38	0.529	0.444	0.973
OBCM	10	0.0087	3.6366	–	–	–	7.746	8.7	6.73	0.05	0.32	0.35	0.484	1.248	1.732
OACC	73	0.0537	2.936	31	0.015	0.42	9.823	9.3	7.74	0.01	0.20	0.23	0.152	1.164	1.316
OACM	132	0.0807	2.434	88	0.043	0.66	10.448	10	10.84	0.01	0.25	0.28	0.000	2.071	2.071
OFC700	26	0.0192	2.9656	6	0.001	0.23	7.127	8.4	8.36	0.01	0.21	0.24	0.000	0.828	0.828
OFM700	236	0.1481	2.516	50	0.073	0.21	10.112	9.7	8.72	0.01	0.26	0.29	0.000	1.727	1.727
OFC800	263	0.1637	2.489	191	0.093	0.72	9.908	9.3	9.03	0.01	0.28	0.32	0.000	2.393	2.393
OFM800	266	0.1552	2.3328	202	0.1	0.76	10.251	10.1	10.82	0.01	0.25	0.29	0.000	2.243	2.243
RB	9	0.0083	3.662	–	–	–	4.672	5.3	3.24	0.16	0.60	0.67	0.897	0.174	1.071
RBCC	4	0.0057	5.776	–	–	–	7.397	7.7	8.04	0.09	0.14	0.20	0.100	0.224	0.323
RBCM	52	0.037	2.854	22	0.01	0.42	7.534	7.7	12.29	0.04	0.25	0.35	0.930	0.817	1.747
RACC	40	0.0327	3.304	8	0.002	0.2	8.227	8.9	14.97	0.02	0.07	0.13	0.100	0.499	0.599
RACM	249	0.483	2.378	208	0.109	0.83	9.295	9.3	19.84	0.01	0.09	0.16	0.477	1.965	2.442
RFC700	41	0.0352	3.428	12	0.011	0.29	7.167	6.6	14.22	0.02	0.30	0.37	0.025	0.396	0.421
RFM700	196	0.133	2.718	126	0.065	0.64	7.695	8.6	16.99	0.02	0.46	0.54	0.496	1.343	1.839
RFC800	88	0.063	2.836	50	0.025	0.56	8.570	8.4	16.58	0.01	0.33	0.39	0.099	0.671	0.770
RFM800	219	0.144	2.614	143	0.075	0.65	7.963	9.6	18.05	0.02	0.46	0.54	0.495	1.372	1.866

6 $${{\text{C}}_{\text{b}}}+{\text{C}}{{\text{O}}_{\text{2}}} \leftrightarrow {\text{2CO}}$$

where: C_b_ – the carbon in biomass or biochar structure.

The molecules of CO_2_ underwent dissociative chemisorption on the surface and formed the C(O) and CO oxides on it. Then, C(O) was desorbed, resulting in the formation of pore structure. CO was a gaseous product that could also be adsorbed on active sites and retarded gasification. The described effect was stronger when microwave heating was applied. During conventional heating only the surface layers of the material were exposed to high temperature and activating agent. Thanks to microwave heating, the both factors also affected deeper layers, which contributed to a significant improvement in textural parameters^53^. Activated carbons obtained from chokeberry seeds and orange peels through direct activation at 800 °C were characterized by the largest S_BET_ parameter and the highest micropore volume among all tested solids. They had the highest total pore volume and the smallest average pore diameter.

**Fig. 1:**
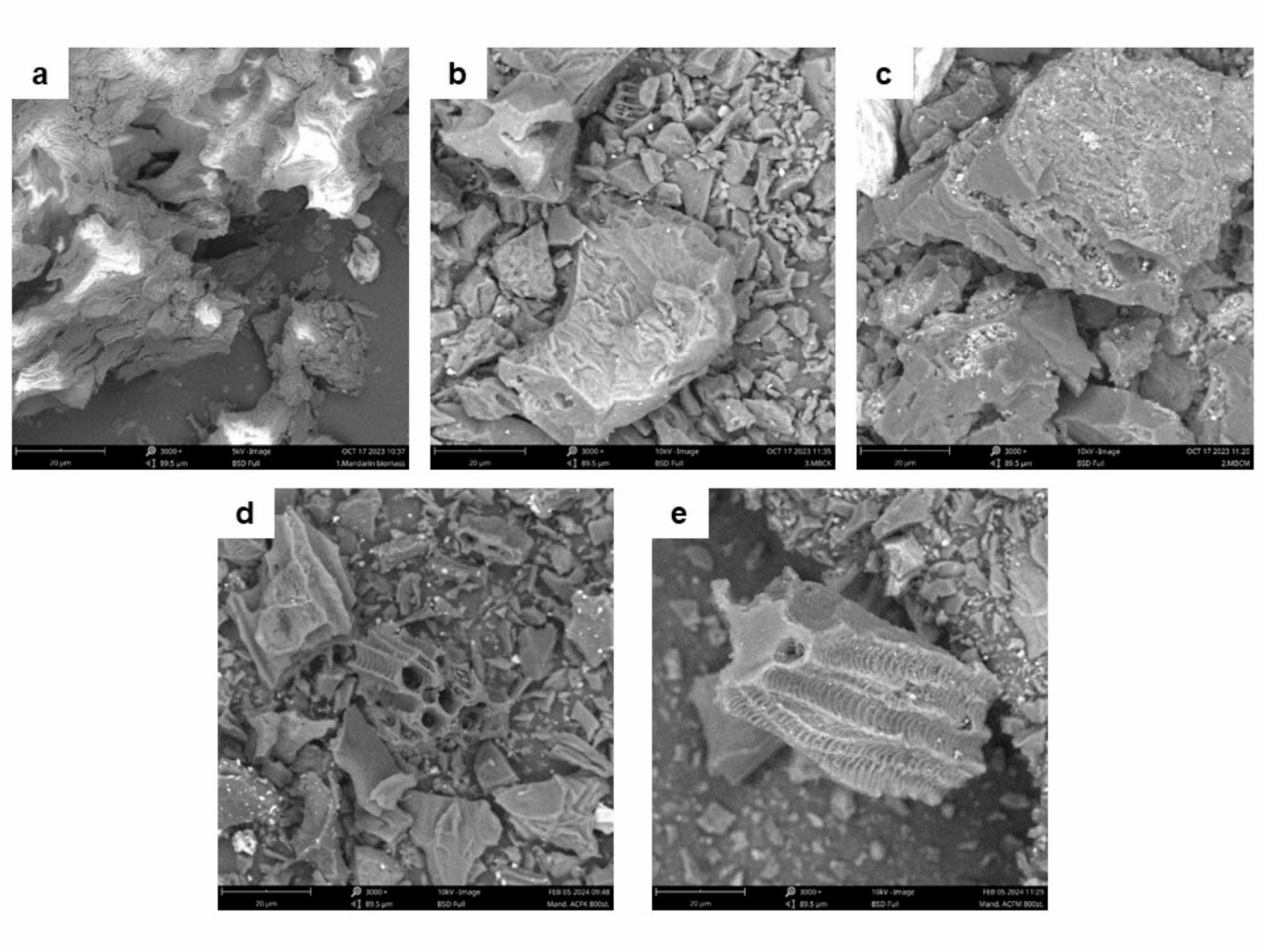
SEM images at 3000× magnification of: orange peels (**a**), biochars obtained from orange peels in a conventional (**b**) and microwave (**c**) furnace at 400 °C, and activated carbons obtained in a conventional (**d**) and microwave (**e**) furnace at 800 °C directly from orange peels.

Scanning election microscopy was used to observe morphology of biomass, biochars, and activated carbons (Fig. 1). All prepared BCs and ACs had a heterogeneous structure rich in cracks, crevices, and channels. Such structures are typical for materials obtained by biomass pyrolysis/activation at high temperatures^54^. Comparing all tested materials, the morphology of activated carbons is the most complex due to the aggregation of mineral compounds^55^.

The acidic/basic groups content in biomass changed after pyrolysis and activation. In most cases, the solids obtained from orange peels had the largest number of functional groups (Table 1). Activation with carbon dioxide increased content of basic groups. For several activated carbons obtained from orange peels, the complete disappearance of acidic groups was visible. Biochars produced at high temperatures (600–700 °C) exhibited highly hydrophobic nature and were characterized by lower contents of H- and O-containing functional groups. This phenomenon was associated with dehydration and deoxygenation of biomass. During heating, chemical bonds in the precursor structure are being broken and rearranged, which resulted in formation of new functional moieties^56^. For example, carbonyl groups crack to CO around 400 °C, whereas carboxyl groups start to decompose into CO_2_ and H_2_O at 200 °C due to lower thermal stability^57^. In most cases, the water extracts of the products of orange peels pyrolysis/activation had the highest pH values among all tested materials.

The results of potentiometric titration indicated that surface charge density and pH_pzc_ of biomass, biochars, and activated carbons differed significantly (Table 1, Fig. S3). The pH_pzc_ value of biomass was in the range of 4.5–5.3. This meant that at pH 6, at which the adsorption study was performed, the biomass surface was negatively charged. All obtained biochars and activated carbons were positively charged during adsorption experiments since their pH_pzc_ values were higher than 8.

The study on elemental composition indicated that the H: C, O: C, and (N + O): C ratios decreased after heat treatment (Table 1). Biomass pyrolysis and activation increased aromaticity and hydrophobicity of the materials as it was indicated by lower values of the H: C and (O + N): C ratios, respectively. The decrease in the O: C ratio was equivalent to lower content of polar functional groups^58,59^. In other words, all activated carbons were more hydrophobic and characterized by higher aromaticity than biochars and biomass, which made them promising adsorbents also for organic, non-polar pollutants. The H: C parameter was also employed to assess the level of biochars carbonization. This parameter is closely associated with the long-term stability of biochar in the environment. According to the European BC Certificate, when selected BC is appropriate for environmental applications, its O: C ratio should be less than 0.4, and H: C, less than 0.7^59^. This criterions were fulfilled for almost all investigated carbon-rich materials. Activated carbons prepared from black currant seeds (RFM700 and RFM800) characterized by the O: C ratio equal to 0.46, were the only exceptions. Spokas^60^ suggested that lower O: C molar ratios were equivalent to longer half-life (t_1/2_) of biochar under laboratory conditions. He identified 3 approximate ranges for the t_1/2_ parameter of BC depending on its O: C ratio. Thus, when O: C is lower than 0.2, BC has t_1/2_ > 1000 years. For the O: C ration in the range of 0.2–0.6, t_1/2_ is between 100 and 1000 years. In turn, when O: C is higher than 0.6, t_1/2_ is lower than 100 years. According to this classification, the produced biochars and activated carbons can be described as those of high stability.

Based on the FTIR spectra (Fig. S4), it was stated that physical activation of biomass, regardless of the heating method, resulted in relatively minor changes in its surface chemistry. The FTIR spectra of biomass, biochars, and activated carbons were similar, and only intensity of specific bands was the difference between them. The FTIR spectrum of orange peels was composed of the following bands at: 3900 –3500 cm^− 1^ (corresponding with the vibrations of free -OH bonds in alcohols, phenols, or other compounds containing hydroxyl groups that are not dissociated^61^), 2278 cm^− 1^ (attributed to C ≡ N stretching vibration), 1700 –1500 cm^− 1^ (stretching vibration of C = O bond in non-ionic carboxyl groups (–COOH, –COOCH_3_), carboxylic acids, or their esters), 1600 –1455 cm^− 1^ (asymmetric and symmetric stretching vibrations of ionic carboxylic groups (–COO^−^)), 1042 cm^− 1^ (the C–O stretching vibrations associated with sugars or esters^62^). These bands were also visible for chokeberry and black currant seeds. Their FTIR spectra contained bands at: 3277 cm^− 1^ (corresponding with –OH stretching vibrations), 2922 –2853 cm^− 1^ (the C–H stretching vibrations), 1634 –1611 cm^− 1^ (the C = O stretching vibration). In the case of biochar and activated carbons, the band at 1042 cm^− 1^ could be attributed to the C–O and C–O–C stretching vibrations associated with phenol, ester, and alcohol groups from cellulose degradation.

The XPS results (Fig. S5, Table S3) confirmed the presence of C = C, C-H, C-O, C-N, COO^−^ groups in orange peels and biochars/activated carbons prepared from them. It was also shown that the materials obtained in a microwave furnace contained higher amount of nitrogen compared to those obtained in a conventional one. Such biochars and activated carbons were characterized by higher content of oxygen, which formed mainly C = O, C-O-C, C = N-O groups. The solids obtained using a conventional heating were rich in the C-OH moieties.

### The Life Cycle Assessment for carbon-rich materials

The environmental impacts of the production, use, and disposal of activated carbon are evaluated using Life Cycle Assessment (LCA). The main environmental impacts of activated carbons are: energy consumption, emissions, resource extraction, and transportation. Notably, due to its higher energy efficiency and lower production and emission of harmful substances such as CO_2_, CH_4_, SO_x_, NO_x_, wastewater, and other waste products, microwave-assisted pyrolysis is considered as a more environmentally friendly method for producing activated carbon than conventional one^63^.

Overall, activated carbon produced from biowaste and agricultural byproducts offers a cost-effective alternative to commercial options, lowering production expenses while also helping to solve waste disposal challenges. In addition to the economic advantages of waste-based activated carbon, assessing its environmental impact is essential. Biomass-based activated carbon typically has a smaller environmental footprint than coal-based activated carbon, especially regarding global warming, acidification, and eutrophication potential^64^. The CO_2_ activation, in contrast to chemical activation with, for example, phosphoric acid (H_3_PO_4_), potentially eliminates the problem of eutrophication, terrestrial ecotoxicity, freshwater aquatic ecotoxicity, human toxicity, and acidification potential^65^.

Yang et al.^66^ provided a study on heating the activated carbon to a temperature of 150 °C. It required 3 min under MW heating, compared to 9 min with electric heating, with the energy consumption ratio between MW and electric heating being 1:22.5. Producing tannery-based sludge activated carbon with MV irradiation in the lab costs approximately 28.63 € per kilogram, with an energy consumption of 0.4 kWh. In comparison, conventional treatment of the same material costs 62.39 € per kilogram and requires 3.54 kWh of energy^67^. It is due the fact, when the MW heating occurs, the quartz vessel absorbs minimal energy, allowing nearly all the microwave energy to be directed toward heating the activated carbon, reducing unnecessary energy consumption. In contrast, conventional heating requires raising the temperature of the entire apparatus before the activated carbon is gradually heated via conduction, resulting in low efficiency and high energy use^67^. The LCA study conducted by Foong et al.^68^ found a 2.5-fold decrease in energy consumption for the microwave heating as well as a reduction of up to 62% in global warming potential. Thus, microwave heating is undoubtedly a method that allows for reducing energy demand and time, but also achieving greater efficiency and better physical and chemical characteristics of the final product, compared to conventional heating. It is also worth mentioning that the MW heating is also used in various fields, like organic chemistry. For example, microwave-assisted C-H activation enables the efficient synthesis of complex molecules, which would otherwise require multiple steps and longer reaction times^69^.

The LCA of orange peels-derived activated carbon, developed in this study, can be considered as similar to that of AC produced from coconut shells or banana peels^70^.

### Selection of optimal precursor for production of carbon-rich materials

The most promising material for further research and applications was selected based on the results of adsorption tests (Fig. S6). The highest adsorption capacities towards selected metal/metalloid ions were noted for orange peels- and black currant seeds-derived materials. For glyphosate, the adsorbents prepared from black currant seeds had better adsorption properties than those obtained from orange peels. However, black currant seeds-derived biochars proved unsuitable for environmental applications – their O: C ratio was too high (as it was mentioned in the previous section). Thus, only the solids obtained from orange peels (OBCC, OBCM, OFC800, OFM800) were selected for further experiments, aiming at the determination of mechanisms of polymers/metals/metalloids adsorption. The most detailed studies were carried out on the adsorbent of the highest efficiency – activated carbon obtained in a microwave furnace at 800 °C directly from orange peels (OFM800). The production of carbon-rich materials from orange peels is economically justified. Orange peels are the most problematic of all biomasses studied due to the huge quantities generated each year.

For initial metal/metalloid concentration equal to 100 mg/L, the adsorption capacity of OFM800 was 3.7, 2.6, 28.7, and 23.4 mg/g for As(V), Se(IV), Cu(II), and Cd(II), respectively. On the other hand, for initial herbicide concentration equal to 10 mg/L, its adsorption capacity towards diuron was 36.57, while that towards glyphosate, 1.26 mg/g. Activated carbon obtained through direct activation of orange peels in a microwave furnace at 800 °C had well-developed surface, i.e., high specific surface area, and was rich in micropores. Because the atoms present within meso- and micropores better interacted with ions than atoms present in macropores, the adsorbed amounts on the microporous solids are usually higher. The adsorption of selected metals and metalloids on orange peels, as well as orange peels-derived biochars and orange peels-derived activated carbons prepared in a conventional furnace or using two-step activation procedure, was clearly lower due to their worse textural parameters or inappropriate surface chemistry.

### Metals/metalloids adsorption mechanisms on the orange peels-derived materials

Adsorption kinetics and isotherms of metal/metalloid ions were determined at pH 6. Then, As(V) ions occurred as H_2_AsO_4_^−^ (85%) and HAsO_4_^2−^ (15%)^71^, Se(IV) ions, as HSeO_3_^−^ (100%)^72^, Cd(II) ions, as Cd^2+^ (100%)^73^, and Cu(II) ions, as Cu^2+^ (almost 100%)^74^.

#### Contact time effect

The experimental kinetics of the Cd(II), Cu(II), As(V), and Se(IV) adsorption on orange peels and orange peels-derived materials with fitting to the Elovich model are presented in Fig. S7. The calculated kinetics parameters are summarized in Table S4. Based on the obtained results it was stated that the investigated adsorption of metal/metalloid ions had two stages, which was especially visible for activated carbons. This suggested strong interactions between active sites of carbon-rich materials and the adsorbates. During the first stage, there was a rapid increase in the adsorbed amount of all ions until thermodynamic equilibrium was achieved (i.e., plateau – the second stage). This state was reached after 60–120 min for Cu(II) and As(V) on all carbonaceous materials as well as for the Se(IV) adsorption on activated carbons. For the Cd(II) adsorption on biochars, the equilibrium was reached later, that is, after 120–240 min. The adsorbed amount of all ions remained unchanged after 24 h, therefore exactly this time was selected for equilibrium tests.

Among all theoretical kinetics models, the Elovich equation best described experimental data. The correlation coefficients were high (R^2^ ≥ 0.994) for the metals/metaloids adsorption on all studied materials (Table S4), and the calculated theoretical q_e_ values were close to the experimental ones. Regarding adsorption on the OFM800 material, experimental and theoretical q_e_ values for As(V) were 4.01 mg/g and 4.06 mg/g, for Se(IV), 2.59 and 2.84 mg/g, for Cd(II), 28.69 and 31.50 mg/g, while for Cu, 23.46 and 25.92 mg/g, respectively. Generally, the Elovich model is suitable for heterogenous adsorbents and allows to predict the mass and surface diffusion as well as the activation and deactivation energy of the system^75,76^. It indicates that chemisorptions, based on the sharing or exchange of electrons between the adsorbent and ions, occurs in the examined systems^77^.

Experimental kinetics data also correlated with the intra-particle diffusion model (R^2^ ≥ 0.997). In this equation, the C parameter informs about thickness of the boundary layer (the higher C value is, the greater thickness of the boundary layer is observed). When the C parameter is higher than 0, the intra-particle diffusion (ion diffusion in the material pores, seen as a plateau) is not the only process controlling the adsorption rate. Then, the external diffusion (ion diffusion towards the external surface, seen as sudden increase at the beginning of the process) also takes place^77^. Among all tested systems, the C parameter was the highest for the Cu adsorption, especially on the activated carbon surface (4.157–11.733), which was a confirmation of two-stage character of this process.

During the adsorption of metal and metalloid ions, chemical interactions between adsorbate and adsorbents were dominant. Complexation, ion exchange, and precipitation could occur in the examined system. Metal complexation takes place when coordination bonds are created between metal ions and biochar/activated carbon functional groups, e.g., carboxyl, phosphoryl and amino ones, according to the reaction^78^:

7 $$\:{Me}^{2+}+\:R-{A}_{i}^{-}\:\leftrightarrow\:R-{A}_{i}{\left(Me\right)}^{+}$$

where: R–A_i_(Me)^+^ is the metal-ligand organic complex.

In turn, when metal ions interact with functional groups like PO_4_^3−^ or CO_3_^2−^, they can precipitate, which is expressed as:

8 $$\:{CO}_{3}^{2-}+{Me}^{2+}\to\:{MeCO}_{3}\downarrow\:$$

9 $$\:{PO}_{4}^{3-}+\:{Me}^{2+}\:\to\:{Me}_{3}{\left({PO}_{4}\right)}_{2}\downarrow\:$$

Ion exchange takes place when ions from the solution pass to the surface and different types of ions are released from the solid at the same time. This process can be described as:

10 $$\:{R-COOMe}_{exch}+\:Me\:\leftrightarrow\:\:R-COOMe+{Me}_{exch}$$

where: Me_exch_ – the exchangeable metal ion, Me – the adsorbed metal ion.

Alkali metal ions (K^+^, Na^+^, Ca^2+^, and Mg^2+^) present on the biochar or activated carbon surface can be exchange with the positively charged metals, e.g., Cu(II), Cd(II), Pb(II)^79,80^. The metalloids adsorption is also based on the formation of hydrogen bonds occurring between O-containing groups of biochar/activated carbon and As(V)/Se(IV) oxyanions:

11 $$\:BC/AC+Metalloid\to\:BC/AC-O\cdots\:Metalloid\:$$

In addition, in the systems containing positively charged solids prepared from orange peels and metalloid oxyanions, electrostatic interactions can favour As(V) and Se(VI) binding.

#### Effect of initial metal/metalloid concentration

**Fig. 2:**
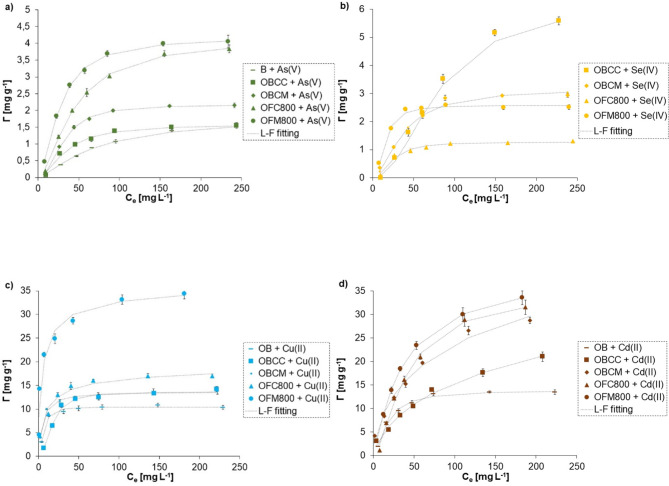
Experimental adsorption isotherms of As (**a**), Se (**b**), Cu (**c**), and Cd (**d**) ions on orange peels (OB) and orange peels-derived materials (OBCC and OBCM – biochars obtained from orange peels in a conventional or microwave furnace at 400 °C; OFC800, OFM800 – activated carbons obtained in a conventional or microwave furnace at 800 °C directly from orange peels) at pH 6, fitted to the Langmuir-Freundlich model.

For isotherm modelling, three-parameter (Langmuir-Freundlich (L-F), Redlich-Peterson (R-P), Dubinin-Radushkevich (D-R)), and two-parameter models (Langmuir, Freundlich, Temkin) were applied. Figure 2 shows experimental isotherms and their fitting to the L-F model, whereas Table S5 presents all calculated isotherm parameters. It was noted that the L-F, R-P, and Temkin models best described the experimental data.

Considering the Langmuir-Freundlich model, the R^2^ coefficient was higher than 0.997 for all tested systems. This model gives information about energy of adsorption (the K_LF_ constant), the number of active sides on the adsorbent (the A_m_ parameter), as well as the solid heterogenity (the m parameter). The highest affinity to the adsorbent was noted for Cu and OFM800 (K_LF_ = 0.318 L mg^− 1^). In contrast, Se(IV) had almost no affinity for the tested solids (KLP was lower than 0.035 L mg^− 1^). The affinity of all ions was clearly higher for the carbon-rich materials produced in a microwave furnace. This was associated with a greater number of adsorbent active sites. The Redlich-Peterson model also fitted the experimental data very well (R^2^ ≥ 0.991). This model is used to the systems, where adsorption process is more complicated and involves homogeneous and heterogeneous adsorption types^59,81^.

The Temkin model assumes that the adsorption heat (the b_T_ parameter) decreases linearly as the coverage of the adsorbent surface increases^82^. When b_T_ constant is less than 1.0 kcal/mol, the adsorption is physical. When b_T_ is in the range of 1–20 kcal/mol, there is ion exchange, whereas when it exceeds 20 kcal/mol, the process is classified as chemisorption^77^. The Temkin model described experimental isotherms with the R^2^ coefficient equal or higher than 0.993. For the As(V) adsorption on orange peels and biochar prepared in a conventional furnace (BCC) as well as the Se(IV) adsorption on activated carbons prepared in conventional and microwave furnace at 800 °C (OFC800 and OFM800), the b_T_ constants of 1.247, 1.240, 1.602, and 1.042 kcal/mol, respectively, were obtained, which confirmed the ion exchange mechanism. In the rest systems, the performed calculations indicated physical adsorption.

Analyzing isotherms, the adsorbed amounts of Cd(II) and Cu(II) ions differed significantly – the Cu(II) adsorption was clearly higher, which was dictated by their various radii and electronegativity. Van der Waals atomic radius of Cu(II) is 140 pm, while that of Cd(II), 158 pm (National Center for Biotechnology Information). Due to the smaller size of Cu(II) ions (compared to Cd ones), they can penetrate pores easier and in larger quantities. During adsorption, negatively charged of As(V) and (Se) oxyanions should be attracted by positive surface of adsorbents and be bound to great extent. However, the adsorption capacity of the adsorbents towards metalloids was significantly lower than that towards metals. This was probably associated with the sizes of As(V) and Se(IV) ions, which are much larger than those of Cd(II) and Cu(II) ones. The van der Waals atomic radii of As(V) and Se(IV) are clearly larger (185 and 190 pm, respectively).

The adsorbed amounts on the materials obtained in a microwave furnace were higher compared to those noticed on the analogous samples prepared under conventional heating. Moreover, the adsorption capacity of activated carbons produced through one-step activation was greater than those from indirect, two-step one. This confirmed that microwave-assisted one-step activation of fruit waste allowed to produce adsorbents of high efficiency. Adsorption capacities of OFM800 and other, described in the literature, activated carbons were compared in Table S6. The capacity of the material obtained from orange peels through direct microwave-assisted, CO_2_-consuming activation was similar to those prepared using multiple-step techniques with chemical reagents. This additionally encourages to perform direct physical activation of fruit waste, which is more eco-friendly, safer, and more economical than the chemical one.

#### Effect of solution pH value

The solution pH value affects adsorbent surface charge, as well as alters adsorbate ionization and species. During the study, for As(V) concentration 100 mg/L, the highest removal efficiency was observed at pH 5 (Γ = 3.94 mg/g) (Fig. S8). For pH 6 and 7, a slight decrease in adsorption capacity was observed (it was equal to 3.69 and 3.01 mg/g, respectively). A similar effect was visible for Se(IV), that is, its adsorbed amount was slightly reduced at higher pH values (2.78, 2.59, and 2.43 mg/g for pH 5, 6, and 7, respectively). At higher pH values, surface charge density of OFM800 was less positive and thus electrostatic attraction between oxyanions and the solid particles were weakened. On the contrary, for Cd(II) and Cu(II), the adsorption capacity of OFM800 increased at higher pH values. When the pH value was higher, the solid surface had a less positive charge. As a result, the electrostatic repulsion between cations and positively charged solid was slightly reduced, and their contact became easier. The adsorbed amounts of Cu(II) on OFM800 were 19.94, 23.40, and 29.22 mg/g, whereas those of Cd(II) were 22.22, 28.66, and 33.76 mg/g at pH 5, 6, and 7, respectively.

#### XPS, EDS and FTIR after adsorption studies with metals and metaloids

**Fig. 3:**
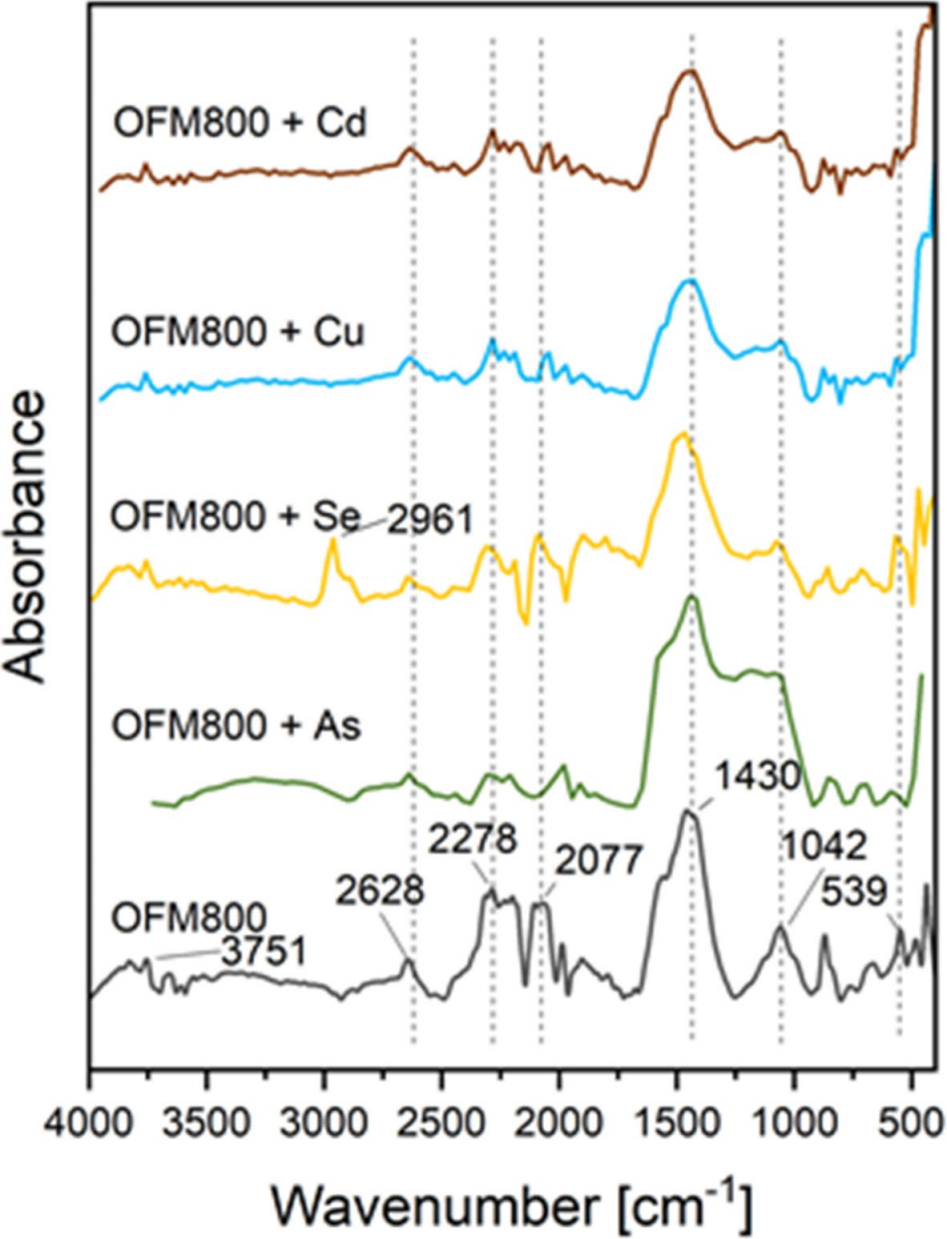
FTIR spectra of the activated carbon obtained in a microwave furnace at 800 °C directly from orange peels (OFM800) with adsorbed metals (Cd/Cu) and metalloids (As/Se).

**Fig. 4:**
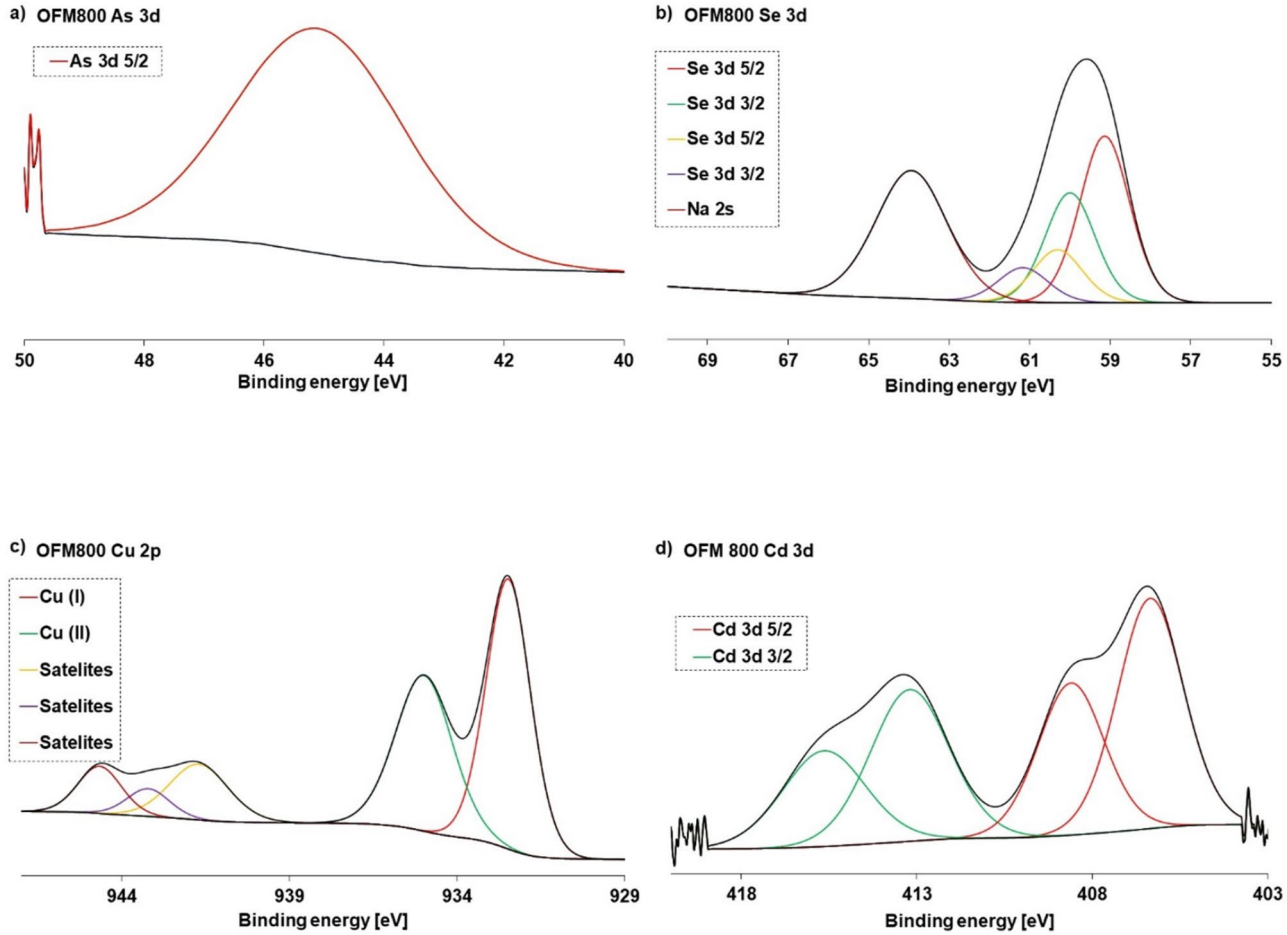
XPS spectra of the activated carbon obtained in a microwave furnace at 800 °C directly from orange peels (OFM800) with adsorbed As (**a**), Se (**b**), Cu (**c**), and Cd (**d**) ions.

The EDS analyses (Figs. S9, S10) confirmed that Cu(II), Cd(II), As(V), and Se(IV) were adsorbed on the OFM800 surface. Additional peaks corresponding to metal/metalloid ions were visible in the spectra. The changes in the FTIR spectra (Fig. 3) after ion adsorption were not significant. The XPS results (Fig. 4) indicated that As(V) was adsorbed as arsenic oxide compounds (45 eV)^83^, whereas Se(IV), as NaSeO_3_ (59 eV) and HSeO_3_ (60–61 eV)^84^. The peaks registered for Cd(II) corresponded to its + 2 oxidation state (413–416 eV, 406–409 eV), namely Cd(OH)^+^ or Cd(OH)_2_^85^. In turn, Cu(II) ions formed Cu_2_O (932 eV) and CuCl_2_ (935 eV) on the solid surface^86^.

#### Adsorption capacity of the OFM800 material towards real-world concentrations

**Fig. 5:**
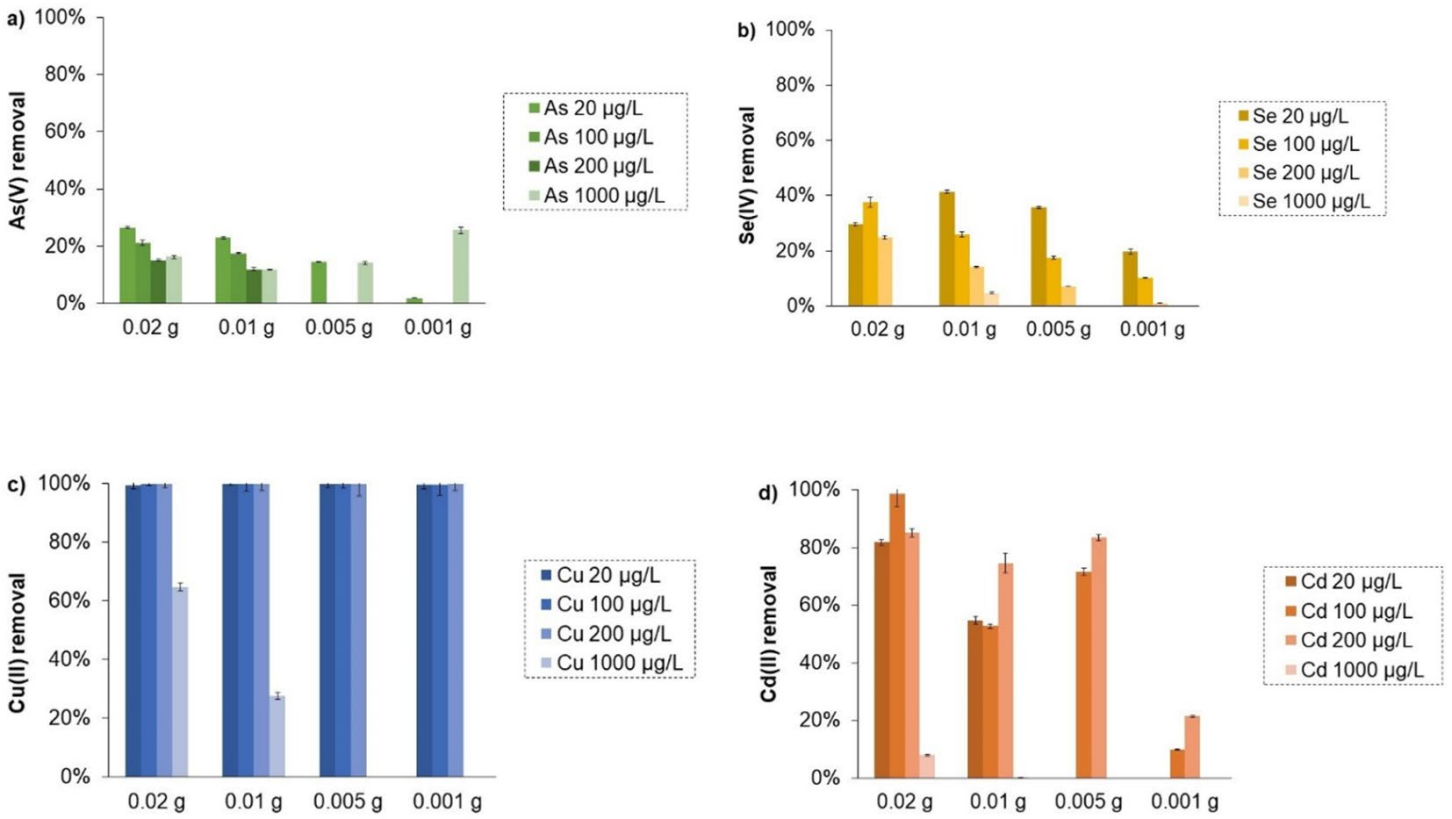
Percentage removal of As (**a**), Se (**b**), Cu (**c**), and Cd (**d**) ions from the solutions containing real-world concentrations (20–1000 µg/L) of pollutants using the activated carbon obtained in a microwave furnace at 800 °C directly from orange peels (OFM800) at different weights.

To reflect real pollution of natural ecosystems, the adsorption measurements were performed for very low concentrations of metals/metalloids, in the range of 20–1000 µg/L. Furthermore, to investigate dependency of the AC weight on adsorption efficiency, the appropriate tests were carried out for the solid weight in the range of 0.001–0.02 g. The obtained results are presented in Fig. 5.

The conducted studies showed that Cu(II) ions had great affinity to the activated carbon obtained in a microwave furnace by direct activation of orange peels at 800 °C. Thus, the selected material was the best adsorbent of exactly these divalent cations. For the initial Cu(II) concentrations of 20–200 µg/L, it adsorbed even 100% of ions, regardless of the applied solid weight. The OFM800 efficiency towards Cd(II) ions was clearly lower, but also satisfactory. It was equal to 80–100%, but for the highest adsorbent weight (0.02 g). The removal of metalloids from aqueous solutions was also of higher performance when their initial concentrations were at a trace level (µg/L). The adsorption efficiency towards Se(IV) was as high as 40% for real-world concentrations, whereas that, measured for initial concentration equal to 100 mg/L, equaled 10.4%. In turn, the As(V) removal was 27.2%, when its initial concentration was 20 µg/L, and 14.8%, for initial As(V) concentration equal to 100 mg/L. This indicated that orange peels-activated carbon obtained in a microwave furnace at 800 °C by direct biomass activation was a good adsorbent of trace amounts of Cu(II), Cd(II), and Se(IV) detected in natural ecosystems.

### Polymer adsorption mechanisms on the orange peels-derived materials

**Fig. 6:**
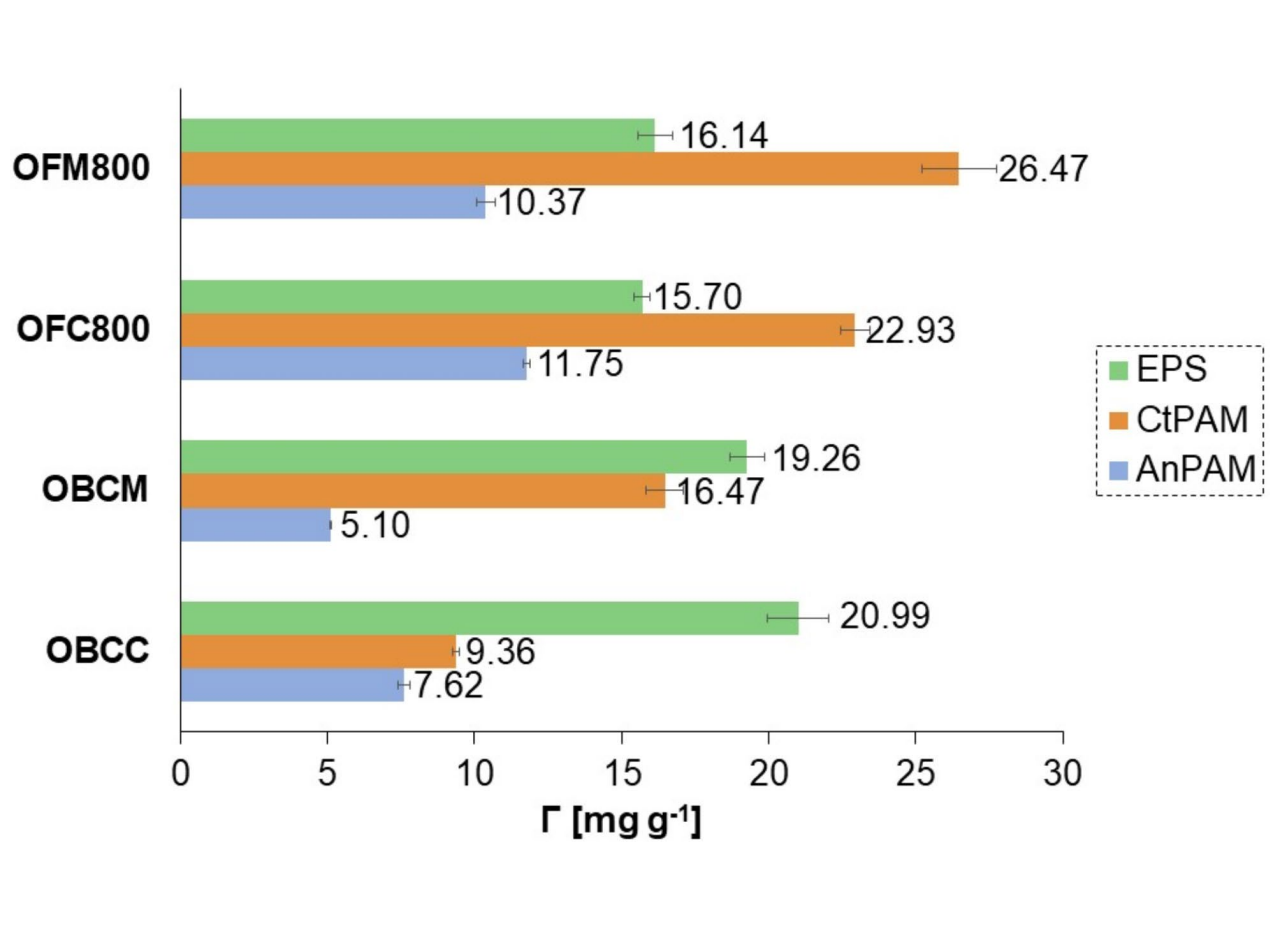
Adsorbed amounts of bacterial exopolysaccharide (EPS), cationic polyacrylamide (CtPAM), and anionic polyacrylamide (AnPAM) on the carbon-rich materials prepared from orange peels at pH 6 (the initial concentration of polymers was 100 mg/L).

**Fig. 7:**
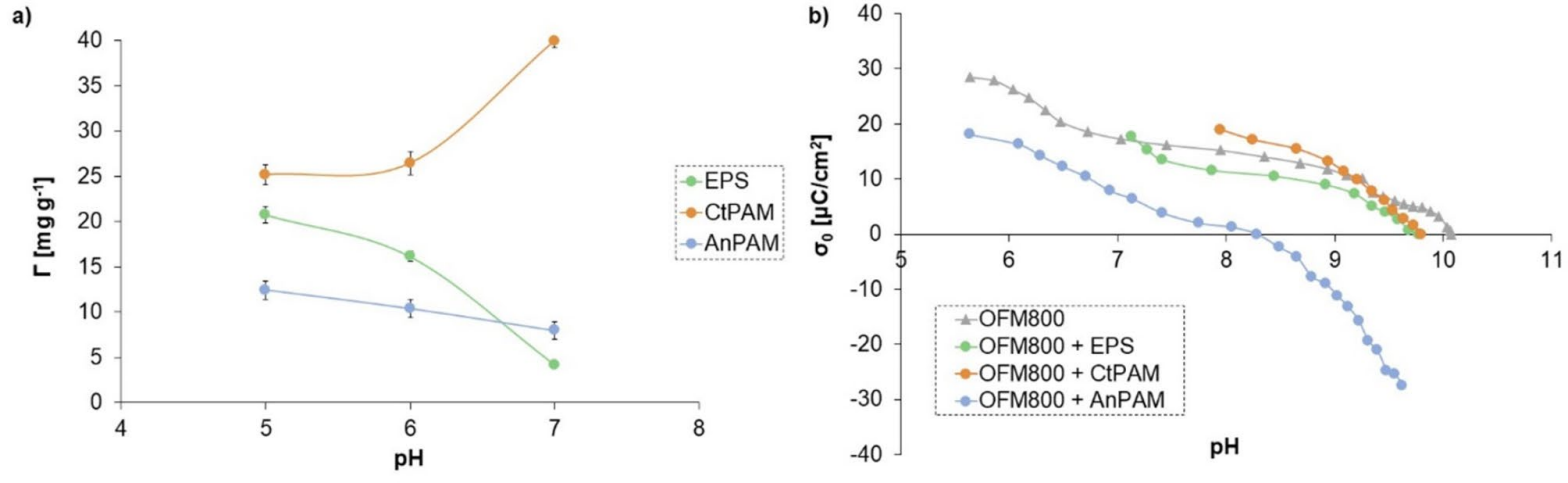
Adsorbed amounts of the bacterial exopolysaccharide (EPS), cationic polyacrylamide (CtPAM), and anionic polyacrylamide (AnPAM) on the activated carbon obtained in a microwave furnace at 800 °C directly from orange peels (OFM800) as a function of pH value (the initial concentration of polymers was 100 mg/L) (**a**), and surface charge density of the OFM800 material with and without polymers (**b**).

The measured amounts of polymers adsorbed on orange peels-derived materials are presented in Fig. 6. The adsorption of ionic macromolecular compounds depended on the solution pH value (Fig. 7a). The EPS and AnPAM adsorption decreased slightly with increasing pH value. For the initial EPS concentration of 100 mg/L, its adsorbed amount was 20.75 mg/g at pH 5, 16.14 mg/g at pH 6, and 4.15 mg/g at pH 7. Carboxylic groups present in the EPS chains underwent gradual dissociation as the solution pH value increased. According to Szewczuk-Karpisz et al.^87^, the pK_a_ parameter of EPS was 5.1, and this meant that at pH 5.1, 50% of carboxylic groups present in the chains of this polymer were dissociated. At pH 7, 98.7% of the groups were dissociated, which was equivalent to more expanded conformation of the macromolecules. Such polymer chains occupied a much larger area of the solid during adsorption, and consequently, the amount of adsorbed EPS was reduced at higher pH value. The EPS and AnPAM adsorption was favoured by electrostatic attraction occurring between the negatively charged macromolecules and the positively charged surface of the solid.

For CtPAM, the tendency was completely different, i.e., its adsorption increased with increasing pH. This was connected with different conformation of polymer chains. AnPAM formed adsorption layer of lower thickness with ‘loops’ and ‘tails’ of short length. In turn, due to the electrostatic repulsion between positively charged CtPAM macromolecules and adsorbent particles, this polymer formed long ‘loops’ and ‘tails’, which limited its contact with the surface. As a result, more chains could fit on a unit area of the solid and the amount of adsorbed polymer was greater. Under the conditions of electrostatic repulsion between AC and macromolecules, the adsorption of polymer chains on the solid surface was based on the formation of hydrogen bonds.

#### Polymer impact on solid surface charge

The polymer modification of OFM800 clearly influenced its surface charge density as well as the pH_pzc_ value (Fig. 7b). This effect was strongly dependent on the type of polymer and the content of ionizable groups in the macromolecules. Typically, dissociated carboxylic groups (-COO^−^) of the polymer fragments located near the surface contribute to the reduction in absolute values of negative σ_0_ parameter. Conversely, the -COO^−^ moieties found in ‘loops’ and ‘tails’ of the adsorbed polymer chains lead to increase in the absolute values of negative σ_0_ parameter^40^. In the analyzed systems, the latter phenomenon prevailed. In the AnPAM presence, a significant increase in the absolute values of negative surface charge was observed, and pH_pzc_ decreased from 10.1 to 8.3. The positive groups still prevailed on the OFM800 surface – the σ_0_ parameter equaled 20 µC/cm^2^ at pH 5, 16.3 µC/cm^2^, pH 6, and 6.3 µC/cm^2^ at pH 7. The EPS adsorption contributed to a slight reduction in the pH_pzc_ value to 9.8 as well as in the absolute values of negative surface charge. This was also induced by the dissociated -COO^−^ groups present in the ‘loops’ and ‘tails’ of the adsorbed polymer chains.

The quaternary amine groups of CtPAM also influenced the OFM800 surface charge. Generally, when positive moieties are situated in segments of the adsorbed polymer (very close to the solid surface), they contribute to an increase in the absolute values of negative surface charge. Conversely, their placement in polymer fragments located in the by-surface layer, resulted in a reduction in the absolute values of negative surface charge^40^. In the case of the examined systems, the influence of CtPAM was not clear. Probably, the the number of positively charged groups in the adsorbed segments and those located within the ‘loops’ and ‘tails’ was very similar. CtPAM caused only a slight reduction in pH_pzc_ to 9.8.

### Adsorption in the mixed systems

#### Mixed systems of metal/metalloid ions and herbicide

**Fig. 8:**
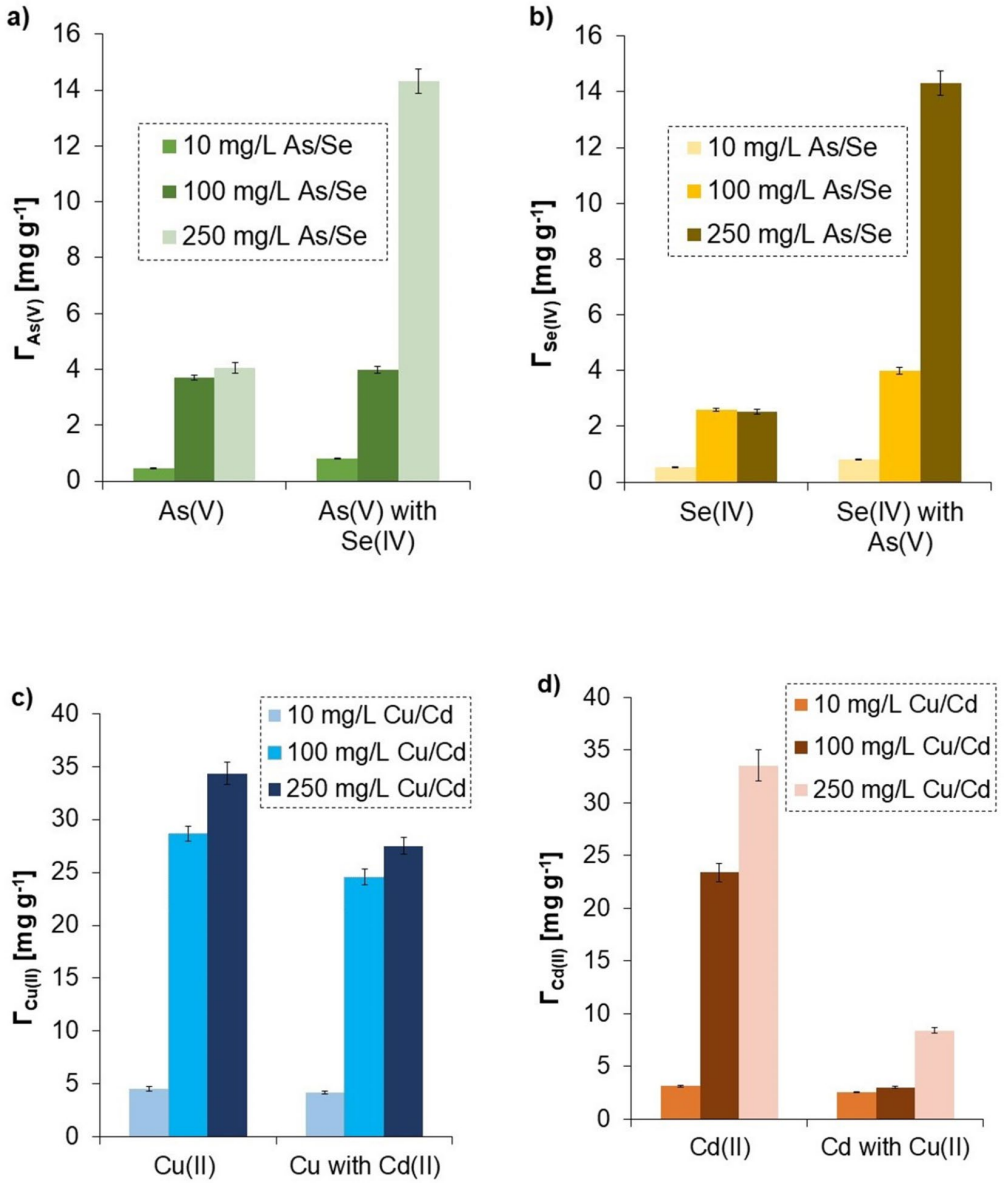
Adsorption capacity of the activated carbon obtained in a microwave furnace at 800 °C directly from orange peels (OFM800) towards metals and metalloids in the one- and two-adsorbate systems at pH 6: the adsorbed amount of As with or without Se (**a**), Se with or without As (**b**), Cu with and without Cd (**c**), Cd with and without Cu (**d**) (the initial concentration of metals/metalloids was 10, 100, or 250 mg/L).

**Fig. 9:**
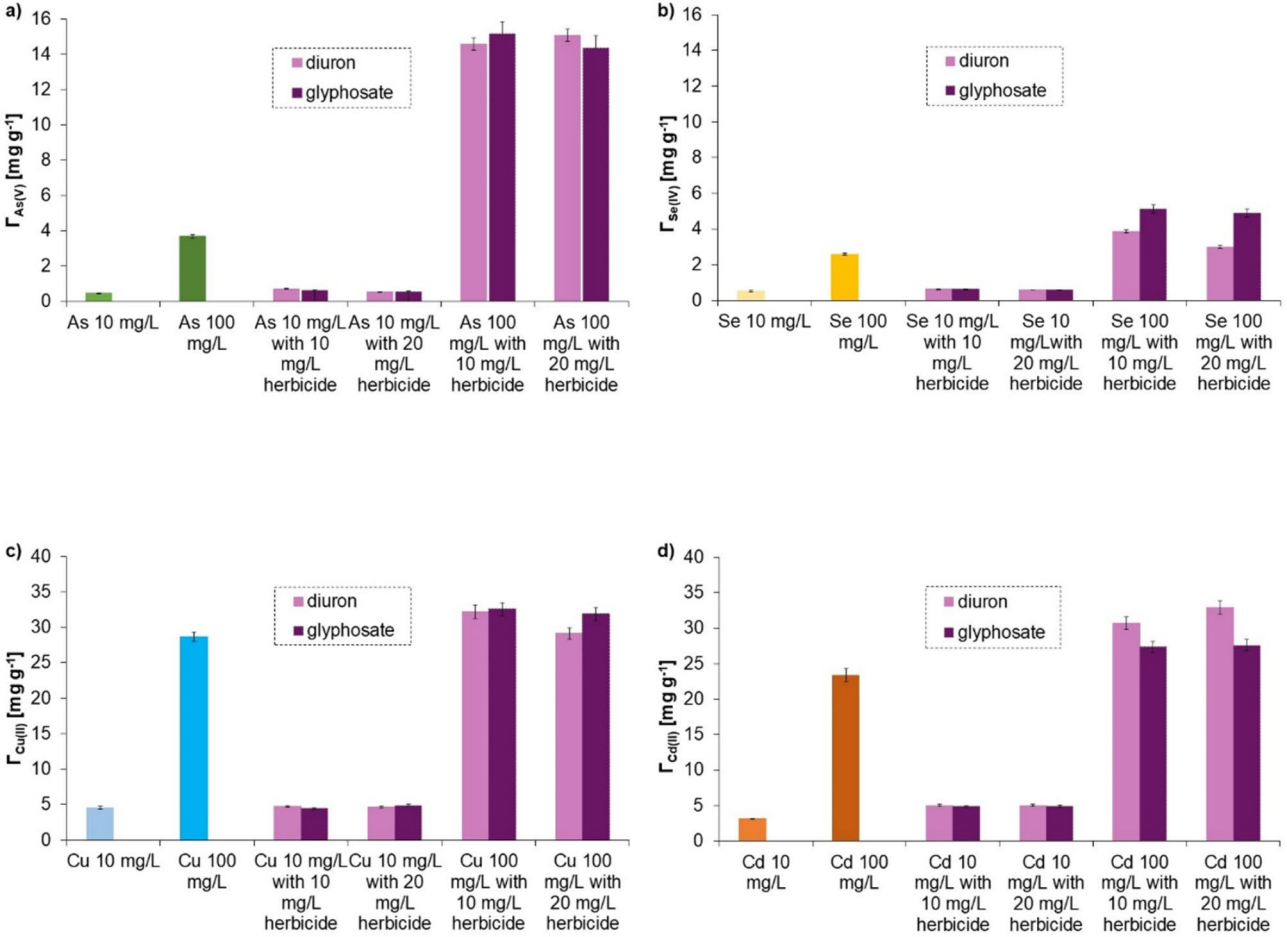
Adsorption capacity of the activated carbon obtained in a microwave furnace at 800 °C directly from orange peels (OFM800) towards metals and metalloids in the one- and two-adsorbate systems at pH 6: the adsorbed amounts of metalloids (As/Se) with or without herbicides (**a**, **b**), and the adsorbed amounts of metals (Cu/Cd) with and without herbicides (**c**, **d**) (the initial concentration of metals/metalloids was 10 or 100 mg/L, whereas that of herbicides, 10 or 20 mg/L).

In the mixed systems containing two metal/metalloid ions (Fig. 8) or one metal/metalloid ion and one herbicide simultaneously (Fig. 9), the adsorption was different than that observed in the solutions with one adsorbate. It was noted that As(V), Se(IV), Cd(II), and Cu(II) ions were adsorbed in larger amounts after addition of diuron and glyphosate. Similarly, when As(V) and Se(IV) were present together in the system, their adsorption was enhanced. In both cases, the formation of complexes between metal and metalloid ions or two metalloid ions based on hydrogen bonds took place^88^. The simultaneous presence of metal ions, Cd(II) and Cu(II), reduced their adsorption on the solid surface, which was dictated by the competition between both cations for active sites.

#### Mixed systems of metal/metalloid ions and macromolecular compounds

**Fig. 10:**
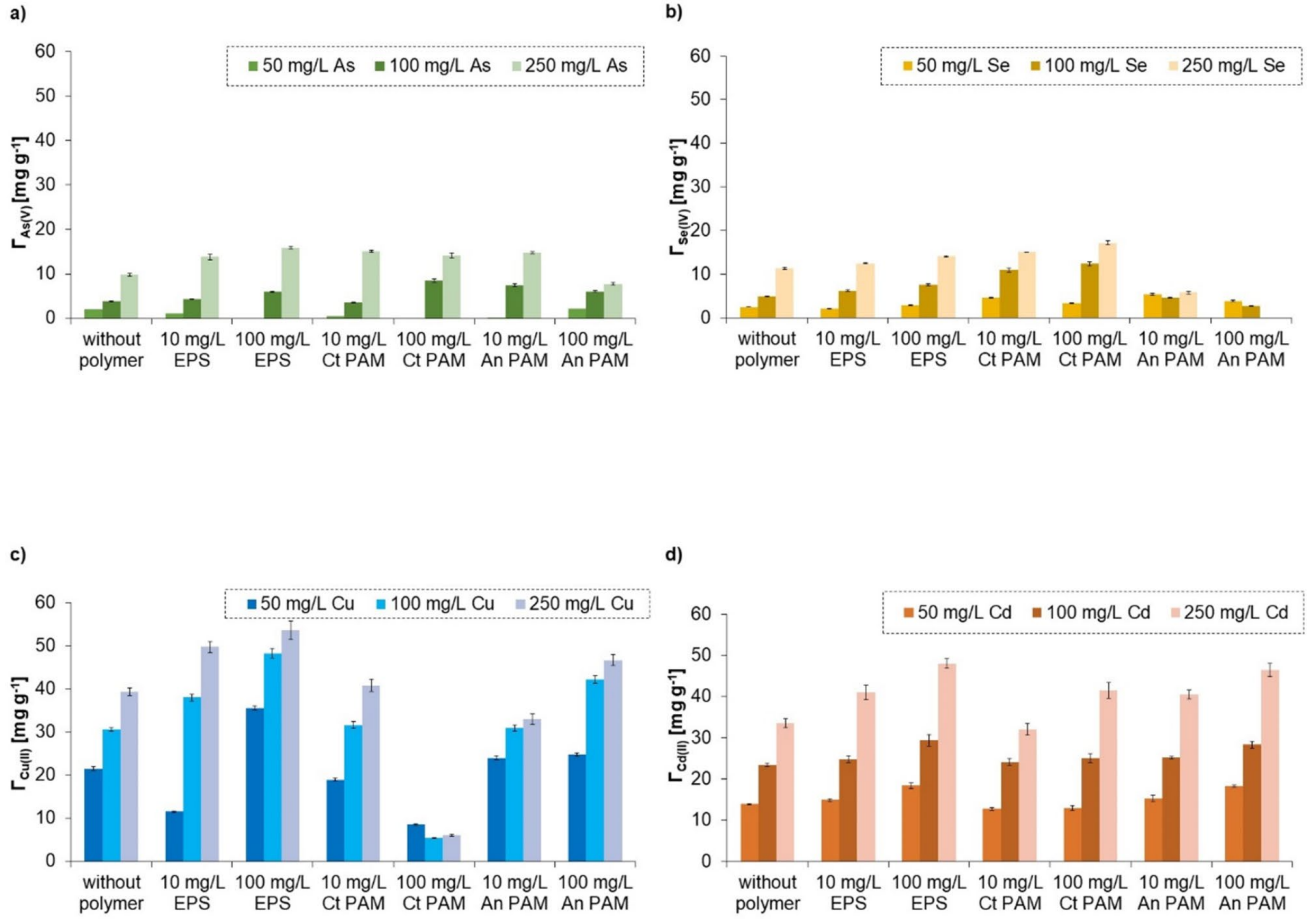
Adsorbed amounts of metals and metalloids (for their initial concentrations of 50, 100, or 250 mg/L) on the activated carbon obtained in a microwave furnace at 800 °C directly from orange peels (OFM800), with and without polymers (added at the concentration of 10 or 100 mg/L).

Modification of activated carbon with polymers also influenced its adsorption capacity towards metals and metalloids (Fig. 10). This was mainly connected with complexation occurring between macromolecular compounds and ions (Table 2). Anionic polymers, i.e., EPS and AnPAM, contributed to higher adsorption of most metals and metalloids on the activated carbon surface. The only one exception was the Se(IV) adsorption in the presence of AnPAM. The adsorbed amounts of Cu(II) and Cd(II) ions were higher even by 1.6 times in the EPS presence. Together with anionic polymers, additional negatively charged groups (e.g., dissociated carboxylic ones, -COO^−^), that attracted metal cations electrostatically, were introduced to the system. The complexation between negative polymer fragments and cations occurred according to the Eqs^89,90^:

**Table 2:** Desorption degree of metals and metalloids from the OFM800 material, with and without polymers as well as complexation degree of metals and metalloids by selected polymer macromolecules.

Desorption degree [%]	As			Se			Cu			Cd		
Cycle number	I	II	III	I	II	III	I	II	III	I	II	III
H_2_O	4.36	0.00	0.00	4.61	0.28	0.00	23.20	21.64	21.12	9.94	2.97	3.08
10 mg/L EPS	7.97	0.86	0.43	4.15	0.22	0.00	8.45	7.48	6.44	5.47	1.33	2.22
100 mg/L EPS	11.12	0.76	0.00	3.56	0.18	0.01	12.18	5.19	0.95	4.34	1.74	0.07
10 mg/L Ct PAM	9.28	1.01	0.00	5.11	0.26	0.01	13.92	17.14	17.51	7.74	5.93	5.22
100 mg/L Ct PAM	11.47	0.75	0.40	3.67	0.27	0.00	44.61	62.20	86.17	7.95	6.64	6.34
10 mg/L An PAM	11.10	1.23	0.05	1.19	0.27	0.04	5.96	4.43	1.16	6.37	1.46	0.16
100 mg/L An PAM	10.15	1.18	0.57	0.72	0.33	0.01	12.71	0.06	0.41	4.23	1.25	0.34

Complexation degree [%]	As			Se			Cu			Cd		
Initial concentration of metal/metalloid	50	100	250	50	100	250	50	100	250	50	100	250
10 mg/L EPS	0.00	0.00	3.83	0.00	0.00	4.84	5.71	8.29	13.14	24.60	25.30	24.40
100 mg/L EPS	0.00	0.00	5.30	0.00	0.00	3.32	2.29	0.29	9.37	26.00	26.00	24.40
10 mg/L Ct PAM	0.00	0.00	3.42	0.00	7.28	3.78	0.00	1.43	11.71	22.80	22.80	22.40
100 mg/L Ct PAM	0.00	10.96	9.31	0.00	7.58	3.50	0.00	0.00	1.60	20.00	24.40	23.60
10 mg/L An PAM	0.00	5.59	2.78	0.00	4.88	2.48	12.00	12.57	12.23	30.00	26.00	24.80
100 mg/L An PAM	0.00	5.36	4.02	0.00	4.22	0.00	32.29	15.57	14.23	40.80	37.40	29.20

12 $$\:\left[{Me}_{t}\right]=\left[{Me}^{2+}\right]+\:\left[{MeA}^{+}\right]+\:\left[{MeA}_{2}\right]$$

13 $$\:\left[{A}_{t}\right]=\left[{A}^{-}\right]+\:\left[HA\right]+\:\left[{MeA}^{+}\right]+\:2\left[{MeA}_{2}\right]$$

where: *[Me*_*t*_*]* and *[A*_*t*_*]* – the total concentrations of metal ions and carboxylic groups, respectively; and *[MeA*^*+*^*]*, *[MeA*_*2*_*]* and *[A*^*−*^*]* – the concentrations of monocarboxylic and dicarboxylic complexes with metal ions and non-complexed carboxylic groups, respectively.

The above complexation was based on the coordination bonds formed in accordance with the theory of hard and soft acids (electron acceptors) and bases (electron donors). The O atoms of anionic macromolecules present in the hydroxyl and carboxyl groups were the main electron donors^89^. The complexes of AnPAM/EPS and metal ions can also be created through ion exchange. The As(V) and Se(IV) oxyanions interacted with anionic polymers and crated complexes via hydrogen bonds. The formed ion-polymer complexes were adsorbed on the activated carbon surface and, as a result, the adsorbed amount of metals and metalloids increased. The formed complexes were of two types: intramolecular or intermolecular. The first ones were created when one divalent cation interacts with two functional groups of one polymer chain, whilst the second, when one divalent cation reacts with two functional groups from two different macromolecules^87^.

Cationic macromolecular compound (CtPAM) has fragments with quaternary amine moieties (-N(CH_3_)_3_^+^) being a source of positive charge as well as neutral amide groups. This cationic polymer also affected adsorption of metals and metalloids on the orange peels-derived activated carbon based on the complexation phenomenon. It made the As(V) and Se(IV) adsorption higher even by 2.2–2.6 times because hydrogen bonds were formed between oxyanions and the polymer fragments. On the other hand, the CtPAM addition significantly reduced adsorption of Cu(II) (by almost 80%). This was a result of strong electrostatic repulsion between positively charged quaternary amine groups of CtPAM and Cu(II) cations. Surprisingly, cationic polymer did not support the Cd(II) binding on the tested solid. Probably, due to differences in metals electronegativity (Cu = 1.90 and Cd = 1.70 in Pauling’s scale), they formed different types of complexes with CtPAM^87^. Neutral amide groups of cationic polyacrylamide have free electron pairs located on the N atom, that may participate in the formation of a covalent bond between CtPAM and cations^91^.

### Desorption of metal/metalloids from the orange peels-derived materials

Desorption study allowed to determine the strength of metal/metalloids adsorption on the activated carbon surface, with and without polymers (Table 2). It was observed that the Cd(II) and Cu(II) desorption from OFM800 was the greatest among all ions. This was probably caused by the electrostatic repulsion occurring between Cd(II)/Cu(II) cations and positively charged OFM800. In the first cycle, desorption degree was in the range 5.96–44.61% for Cu(II) and 4.23–9.44% for Cd(II). The As(V) and Se(IV) oxyanions, that were electrostatically attracted to the surface, the desorption degree was much lower, i.e., in the range of 0.72–4.36%.

The immobilization of metals/metalloids was enhanced by polymers. A clear reduction in desorption was observed for Se(IV) with AnPAM as well as Cu(II)/Cd(II) with EPS/AnPAM. Then, ion-polymer complexation as well as polymer adsorption on the solid surface limited the leaching of metals and metalloids and thus reduced their bioavailability.

## Conclusions

The authors developed fruit waste-derived activated carbons through CO_2_-consuming, microwave-assisted method using shorter time and lower temperature than those recommended for this type of activation. The use of microwaves and one-stage activation allowed for more effective surface development while reducing energy input. As a result, reduced environmental footprint of the conducted process was achieved.

The activated carbon obtained by direct CO_2_-activation of orange peels in a microwave furnace (OFM800) was the most efficient towards metals and metalloids. What is more, it showed satisfactory adsorptive abilities relative to macromolecular compounds as well as was suitable for environmental applications. For initial metal concentration of 100 mg/L, the selected material adsorbed 57.32% of Cu(II) ions and 46.8% of Cd(II) ones. Surprisingly, these values increased in the mixed systems with diuron (10 mg/L) to 64.42% and 61.5%, respectively. The polymer addition clearly increased adsorption efficiency of OFM800. The most desirable phenomena were observed in the solutions with anionic polymers and metal cations. Then, the Cu(II) removal rate was equal to even 96.52% in the EPS presence (100 mg/L). In the systems containing real-world concentrations of metals/metalloids (20–200 µg/L), the OFM800 material removed as much as 100% of Cu(II) ions. Furthermore, it showed relatively high capacity towards Cd(II) and Se(II) ions. Taking into account the above facts, it can be stated that the produced material is characterized by a good ability to bind toxic compounds in the systems with complex composition and can be considered as that of great importance to the environment.

## Electronic supplementary material

Below is the link to the electronic supplementary material.

Supplementary Material 1

## Data Availability

Data will be made available on request to the corresponding author.

## Abbreviations

ABCC: The biochar obtained from chokeberry seeds in a conventional furnace at 400 °C
ABCM: The biochar obtained from chokeberry seeds in a microwave furnace at 400 °C
AACC: The activated carbon obtained in a conventional furnace at 800 °C from ABCC
AACM: The activated carbon obtained in a microwave furnace at 800 °C from ABCM
AFC700: The activated carbon obtained in a conventional furnace at 700 °C directly from chokeberry seeds
AFC800: The activated carbon obtained in a conventional furnace at 800 °C directly from chokeberry seeds
AFM700: The activated carbon obtained in a microwave furnace at 700 °C directly from chokeberry seeds
AFM800: The activated carbon obtained in a microwave furnace at 800 °C directly from chokeberry seeds
OBCC: The biochar obtained from orange peels in a conventional furnace at 400 °C
OBCM: The biochar obtained from orange peels in a microwave furnace at 400 °C
OACC: The activated carbon obtained in a conventional furnace at 800 °C from OBCC
OACM: The activated carbon obtained in a microwave furnace at 800 °C from OBCM
OFC700: The activated carbon obtained in a conventional furnace at 700 °C directly from orange peels
OFC800: The activated carbon obtained in a conventional furnace at 800 °C directly from orange peels
OFM700: The activated carbon obtained in a microwave furnace at 700 °C directly from orange peels
OFM800: The activated carbon obtained in a microwave furnace at 800 °C directly from orange peels
RBCC: The biochar obtained from black currant seeds in a conventional furnace at 400 °C
RBCM: The biochar obtained from black currant seeds in a microwave furnace at 400 °C
RACC: The activated carbon obtained from black currant seeds in a conventional furnace at 800 °C from BCC
RACM: The activated carbon obtained from black currant seeds in a microwave furnace at 800 °C from BCM
RFC700: The activated carbon obtained in a conventional furnace at 700 °C directly from black currant seeds
RFC800: The activated carbon obtained in a conventional furnace at 800 °C directly from black currant seeds
RFM700: The activated carbon obtained in a microwave furnace at 700 °C directly from black currant seeds
RFM800: The activated carbon obtained in a microwave furnace at 800 °C directly from black currant seeds
