# Supplementary material for: New fruit waste-derived activated carbons of high adsorption performance towards metal, metalloid, and polymer species in multicomponent systems
Source: Sci Rep. 2025 Jan 7;15:1082. doi: 10.1038/s41598-025-85409-0 (PMC11706987; doi:10.1038/s41598-025-85409-0)
Supplement: Supplementary file 1 — Supplementary Material 1 [file 41598_2025_85409_MOESM1_ESM.docx]

SUPPLEMENTARY INFORMATION

**New fruit waste-derived activated carbons of high adsorption performance towards metal, metalloid, and polymer species in multicomponent systems**

**Sylwia Kukowska^1^, Piotr Nowicki^2^, Katarzyna Szewczuk-Karpisz^1*^**

^1^ Institute of Agrophysics, Polish Academy of Sciences, Doświadczalna 4, 20-290 Lublin, Poland;

^2^ Department of Applied Chemistry, Faculty of Chemistry, Adam Mickiewicz University in Poznań, Uniwersytetu Poznańskiego 8, 61-614 Poznań, Poland.

*k.szewczuk-karpisz@ipan.lublin.pl

Tab. S1. Structures of herbicides and macromolecules used in the experiments

| Name | Structure |
| --- | --- |
| *Rhizobium leguminosarum* bv.  *trifolii* exopolysaccharide (EPS) monomer | 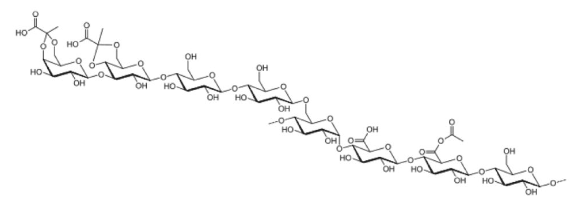  [1] |
| Cationic polyacrylamide (CtPAM) monomer | 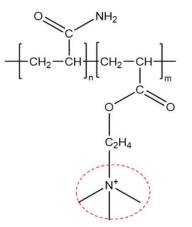 [2] |
| Anionic polyacrylamide (AnPAM) monomer | 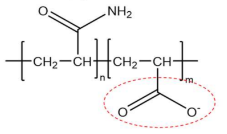 [2] |
| Diuron, DCMU, N-(3,4-dichlorophenyl)-N,N-dimethyl-urea | 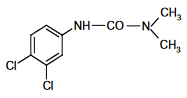 [3] |
| Glyphosate, GLY, N-(phosphonomethyl) glycine | 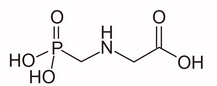 [4] |

Tab. S2. Theoretical models applied in the experimental data modelling

| **Equation** | **Formula** | **References** |
| --- | --- | --- |
| Pseudo I-order | $q_{t}=\frac{k_{2}\cdot q_{e}^{2}\cdot t}{{1+k}_{2}\cdot q_{e}\cdot t}$ (3) | [5] |
| Pseudo II-order | $q_{t}=q_{e}\cdot{(1-e^{-k_{1}\cdot t)}t}^{\frac{1}{2}}$ (4) | [6] |
| IPD | $q_{t}=k_{D}t^{\frac{1}{2}}+C$ (5) | [7] |
| Elovich | $q_{t}=\frac{1}{\beta}\ln(1+\alpha\beta t)$ (6) | [7] |
| Langmuir | $q_{e}=\frac{Q_{m}K_{L} C_{e}}{1+K_{L}C_{e}}$ (7) | [8] |
| Freundlich | $q_{e}=K_{F}\cdot C_{e}^{1/n}$ (8) | [8] |
| Langmuir-Freundlich | $q_{e}=\frac{A_{m}\cdot K_{LF}\cdot C_{e}^{m}}{1+K_{LF\cdot}{C_{e}}^{m}}$ (9) | [9] |
| Redlich-Peterson | $q_{e}=\frac{K_{R}\cdot C_{e}}{1+a_{R}{{\cdot C}_{e}}^{\beta_{R}}}$ (10) | [10] |
| Temkin | $q_{e}=\frac{RT}{b} lnAC_{e}$ (11) | [11] |
| Dubinin-Radushkevich | $q_{e}=q_{D}\cdot e^{(-\beta_{D}{[RTln(1+\frac{1}{C_{e}}]}^{2})}$ (12) | [10] |

*q_t_* (mg/g) – the amount adsorbed at time *t* (min); *q_e_* (mg/g) – the amount adsorbed at equilibrium; *k_1_* (1/min) and *k_2_* (g/mg·min) – the reaction rate constants; *C_e_* (mg/L) – the equilibrium concentration of adsorbate in the solution; *C* (mg/g) – the constant; *k_D_* (g/mg·min^1/2^) – the IPD rate constant; *α* (mg/g·min) – the primary rate of adsorption; *β* – the desorption parameter; *Q_m_* (mg/g) – the maximum adsorbed amount in the monomolecular layer; *K_L_* (L/mg) – the Langmuir constant; *K_F_* (mg/g (mg/L)^1/n^) – the Freundlich parameter; *K_LF_,* (L/mg) – related to the affinity of the adsorbate for active sites; *A_m_* (mg/g) – the amount of the available surface sites; *m* – the parameter determining the shape of the energy distribution function; *K_R_* (L/mg) – the Redlich–Peterson adsorption capacity constant; *a_R_* [(L/mg)^β^] – the Redlich–Peterson isotherm constant; *β_R_* – the exponent between 0 and 1; *A* – the Temkin model constant; *b* (kcal/mol) – the adsorption heat; *R* (8.314 J/K·mol) – the universal gas constant; T (K) – the absolute temperature; *q_D_* (mg/g) – the theoretical isotherm saturation capacity; *β_D_* – the Dubinin-Raduskevich isotherm constant.

Tab. S3. Content of individual forms of carbon, nitrogen, oxygen, calcium, and potassium in orange peels (OB) and carbon-rich materials prepared from them

| Sample | OB | OBCC | OBCM | OFCC800 | OFCM800 |
| --- | --- | --- | --- | --- | --- |
| **C_total_ [at. %]** | **77.96** | **86.41** | **81.70** | **84.41** | **76.96** |
| C=C / C-H | 43.30 | 68.24 | 57.83 | 67.10 | 52.73 |
| C-O / C-N | 20.27 | 11.49 | 17.34 | 22.43 | 15.69 |
| C=O | 11.37 | 4.05 | 3.61 | 5.83 | 4.72 |
| COO^-^ | 3.02 | 2.63 | 2.91 | 4.64 | 3.82 |
| **N_total_ [at. %]** | **1.41** | **0.50** | **2.39** | **1.44** | **2.13** |
| N-5 (pyrrolic / pyridonic) | 1.41 | 0.50 | 2.39 | 1.44 | 2.13 |
| **O_total_ [at. %]** | **20.63** | **13.09** | **15.90** | **12.72** | **17.9** |
| C=O | 5.98 | 4.14 | 11.75 | 1.79 | 12.26 |
| C-OH | - | 8.95 | - | 10.93 | - |
| C-O-C / C=N-O | 14.65 | - | 4.15 | - | 5.64 |
| COO^-^ | - | - | - | - | - |
| **Ca_total_ [at. %]** | **-** | **-** | **-** | **1.43** | **1.86** |
| Ca 2p 3/2 | - | - | - | 0.95 | 1.24 |
| Ca 2p 1/2 | - | - | - | 0.47 | 0.62 |
| **K_total_ [at. %]** | **-** | **-** | **-** | **0.01** | **1.14** |
| K 2p 3/2 | - | - | - | 0.01 | 0.76 |
| K 2p 1/2 | - | - | - | - | 0.38 |

Tab. S4. Equilibrium parameters for Cd(II), Cu(II), As(V), and Se(IV) adsorption on orange peels as well as carbon-rich materials obtained using them

|  |  | **As(V)** | | | | | **Se(IV)** | | | | | **Cd(II)** | | | | | **Cu(II)** | | | | |
| --- | --- | --- | --- | --- | --- | --- | --- | --- | --- | --- | --- | --- | --- | --- | --- | --- | --- | --- | --- | --- | --- |
| Model | parameter | OB | OBCC | OBCM | OFC800 | OFM800 | OB | OBCC | OBCM | OFC800 | OFM800 | OB | OBCC | OBCM | OFC800 | OFM800 | OB | OBCC | OBCM | OFC800 | OFM800 |
| **Langmuir (linear)** | Q_m_ [mg/g] | 2.892 | 2.950 | 4.210 | 7.220 | 5.033 | - | 25.875 | 3.880 | 1.872 | 2.720 | 15.270 | 26.243 | 33.543 | 91.519 | 42.071 | 10.756 | 16.187 | 14.076 | 17.685 | 35.245 |
|  | K_L_ [L/mg] | 0.005 | 0.006 | 0.006 | 0.006 | 0.022 | - | 0.001 | 0.017 | 0.013 | 0.074 | 0.045 | 0.016 | 0.028 | 0.004 | 0.022 | 0.226 | 0.036 | 0.183 | 0.132 | 0.165 |
|  | R^2^ | 0.802 | 0.378 | 0.320 | 0.538 | 0.956 | - | 0.184 | 0.954 | 0.702 | 0.985 | 0.984 | 0.956 | 0.969 | 0.135 | 0.999 | 0.998 | 0.971 | 0.999 | 0.999 | 0.999 |
| **Freundlich (linear)** | n | 1.187 | 1.154 | 1.133 | 1.161 | 1.649 | - | 1.042 | 1.550 | 1.367 | 2.422 | 2.039 | 1.968 | 2.345 | 1.051 | 1.631 | 3.764 | 1.891 | 4.026 | 3.604 | 3.260 |
|  | K_F_ [mg/g (mg/L)^1/n^] | 1.023 | 0.823 | 0.564 | 0.346 | 0.286 | - | 0.306 | 0.419 | 0.910 | 0.410 | 0.078 | 0.067 | 0.044 | 0.035 | 0.034 | 0.094 | 0.076 | 0.074 | 0.060 | 0.028 |
|  | R^2^ | 0.939 | 0.767 | 0.748 | 0.853 | 0.818 | - | 0.928 | 0.890 | 0.708 | 0.678 | 0.777 | 0.985 | 0.987 | 0.829 | 0.948 | 0.685 | 0.743 | 0.800 | 0.930 | 0.770 |
| **Langmuir-Freundlich** | A_m_ [mg/g] | 1.798 | 1.588 | 2.171 | 4.104 | 4.177 | - | 6.219 | 3.154 | 1.276 | 2.579 | 13.923 | 46.504 | 58.393 | 34.614 | 39.431 | 10.430 | 13.666 | 13.698 | 19.670 | 36.857 |
|  | K_LF_ [L/mg] | 0.003 | 0.002 | 0.000 | 0.002 | 0.004 | - | 0.000 | 0.002 | 0.001 | 0.002 | 0.012 | 0.019 | 0.035 | 0.003 | 0.018 | 0.032 | 0.002 | 0.183 | 0.199 | 0.318 |
|  | m | 1.353 | 1.775 | 2.222 | 1.627 | 1.680 | - | 2.096 | 1.779 | 2.107 | 2.255 | 1.558 | 0.705 | 0.643 | 1.533 | 1.102 | 1.912 | 2.130 | 1.098 | 0.684 | 0.699 |
|  | R^2^ | 0.999 | 0.998 | 0.996 | 0.997 | 0.999 | - | 0.923 | 0.998 | 0.998 | 0.999 | 0.999 | 0.999 | 0.999 | 0.997 | 0.999 | 0.999 | 0.999 | 0.999 | 0.994 | 0.998 |
| **Redlich-Peterson** | K_R_ [L/mg] | 0.021 | 0.120 | 0.721 | 0.074 | 0.140 | - | 0.049 | 0.074 | 0.047 | 0.181 | 0.785 | 0.855 | 5.220 | 0.622 | 0.960 | 46.910 | 0.756 | 2.907 | 3.849 | 14.590 |
|  | n | 0.899 | 0.678 | 0.612 | 1.000 | 1.000 | - | 1.000 | 1.000 | 1.000 | 1.000 | 1.000 | 0.645 | 0.616 | 1.000 | 1.000 | 0.843 | 1.000 | 1.000 | 0.908 | 0.909 |
|  | a_R_ [(L/mg)^β^] | 0.016 | 0.385 | 2.283 | 0.014 | 0.028 | - | 0.004 | 0.019 | 0.030 | 0.062 | 0.051 | 0.236 | 1.250 | 0.014 | 0.023 | 9.780 | 0.047 | 0.209 | 0.348 | 0.668 |
|  | R^2^ | 0.996 | 0.998 | 0.989 | 0.993 | 0.996 | - | 0.989 | 0.998 | 0.997 | 0.999 | 0.997 | 0.999 | 0.998 | 0.991 | 0.995 | 0.996 | 0.998 | 0.999 | 0.999 | 0.998 |
| **Temkin** | A | 0.101 | 0.154 | 0.163 | 0.128 | 0.243 | - | 0.068 | 0.175 | 0.231 | 0.801 | 0.483 | 0.278 | 0.669 | 0.135 | 0.294 | 5.047 | 0.435 | 7.104 | 4.132 | 6.104 |
|  | b [kcal/mol] | 1.247 | 1.240 | 0.875 | 0.488 | 0.523 | - | 0.295 | 0.669 | 1.602 | 1.042 | 0.178 | 0.125 | 0.108 | 0.058 | 0.070 | 0.347 | 0.170 | 0.287 | 0.217 | 0.114 |
|  | R^2^ | 0.995 | 0.995 | 0.998 | 0.989 | 0.996 | - | 0.999 | 0.997 | 0.997 | 0.999 | 0.997 | 0.999 | 0.998 | 0.985 | 0.994 | 0.997 | 0.998 | 0.996 | 0.993 | 0.996 |
| **Dubinin-Radushkevich** | K_DR_ | 0.000 | 0.000 | 0.000 | 0.000 | 0.000 | - | 0.000 | 0.000 | 0.000 | 0.000 | 0.000 | 0.000 | 0.000 | 0.000 | 0.000 | 0.000 | 0.000 | 0.000 | 0.000 | 0.000 |
|  | q_D_ | 0.974 | 1.315 | 1.904 | 2.856 | 3.418 | - | 4.568 | 2.312 | 1.202 | 2.497 | 11.971 | 11.967 | 17.139 | 21.835 | 20.740 | 10.232 | 12.294 | 12.520 | 14.247 | 29.005 |
|  | R^2^ | 0.834 | 0.981 | 0.982 | 0.930 | 0.944 | - | 0.919 | 0.857 | 0.995 | 0.986 | 0.960 | 0.597 | 0.620 | 0.939 | 0.774 | 0.993 | 0.957 | 0.963 | 0.811 | 0.947 |

Tab. S5. Kinetics parameters for Cd(II), Cu(II), As(V), and Se(IV) adsorption on orange peels as well as carbon-rich materials obtained using them

|  |  | **As(V)** | | | | | **Se(IV)** | | | | | **Cd(II)** | | | | | **Cu(II)** | | | | |
| --- | --- | --- | --- | --- | --- | --- | --- | --- | --- | --- | --- | --- | --- | --- | --- | --- | --- | --- | --- | --- | --- |
| Model | parameter | OB | OBCC | OBCM | OFC800 | OFM800 | OB | OBCC | OBCM | OFC800 | OFM800 | OB | OBCC | OBCM | OFC800 | OFM800 | OB | OBCC | OBCM | OFC800 | OFM800 |
| **PFO (linear)** | q_e_ [mg/g] | 0.860 | 1.474 | 1.759 | 2.394 | 1.782 | - | 3.384 | 2.424 | 0.595 | 0.952 | 10.355 | 18.812 | 33.334 | 23.388 | 21.877 | 3.367 | 5.174 | 6.167 | 7.600 | 11.176 |
|  | k_1_ [1/min] | 0.013 | 0.026 | 0.047 | 0.033 | 0.017 | - | 0.045 | 0.052 | 0.043 | 0.044 | 0.043 | 0.037 | 0.052 | 0.041 | 0.039 | 0.033 | 0.035 | 0.040 | 0.027 | 0.066 |
|  | R^2^ | 0.951 | 0.997 | 0.948 | 0.906 | 0.901 | - | 0.979 | 0.964 | 0.922 | 0.949 | 0.951 | 0.965 | 0.907 | 0.995 | 0.997 | 0.818 | 0.858 | 0.987 | 0.936 | 0.838 |
| **PSO (linear)** | q_e_ [mg/g] | 7.573 | 5.170 | 2.945 | 5.046 | 4.073 | - | 3.774 | 3.220 | 1.257 | 2.629 | 20.207 | 20.207 | 20.207 | 24.581 | 26.110 | 10.553 | 12.866 | 13.264 | 16.872 | 29.172 |
|  | k_2_ [g/mg·min] | 0.000 | 0.000 | 0.002 | 0.001 | 0.016 | - | 0.009 | 0.007 | 0.065 | 0.058 | 0.000 | 0.000 | 0.000 | 0.001 | 0.001 | 0.014 | 0.006 | 0.008 | 0.005 | 0.005 |
|  | R^2^ | 0.005 | 0.068 | 0.507 | 0.459 | 0.998 | - | 0.999 | 0.973 | 0.999 | 0.999 | 0.176 | 0.176 | 0.176 | 0.991 | 0.993 | 0.999 | 0.998 | 0.999 | 0.999 | 0.999 |
| **IPD** | k_D_ [g/mg·min^1/2^] | 0.045 | 0.072 | 0.106 | 0.150 | 0.134 | - | 0.145 | 0.135 | 0.050 | 0.125 | 0.379 | 0.746 | 1.027 | 1.102 | 1.174 | 0.365 | 0.522 | 0.452 | 0.610 | 1.071 |
|  | C [mg/g] | 0.000 | 0.000 | 0.094 | 0.210 | 1.250 | - | 0.520 | 0.320 | 0.354 | 1.140 | 0.000 | 0.000 | 0.000 | 1.356 | 2.460 | 4.130 | 4.039 | 5.503 | 5.530 | 11.125 |
|  | R^2^ | 0.998 | 0.999 | 0.996 | 0.987 | 0.987 | - | 0.978 | 0.990 | 0.996 | 0.976 | 0.997 | 0.997 | 0.997 | 0.999 | 0.999 | 0.998 | 0.999 | 0.999 | 0.998 | 0.999 |
| **Elovich** | α [mg/g· min^]^ | 0.013 | 0.021 | 0.077 | 0.137 | 3.736 | - | 0.250 | 0.162 | 0.490 | 5.738 | 0.095 | 0.180 | 0.241 | 0.641 | 1.113 | 14.640 | 4.257 | 100.686 | 10.069 | 49.614 |
|  | β | 3.266 | 2.074 | 1.861 | 1.222 | 2.000 | - | 1.315 | 1.440 | 5.150 | 3.153 | 0.353 | 0.175 | 0.125 | 0.166 | 0.176 | 0.760 | 0.483 | 0.790 | 0.421 | 0.286 |
|  | R^2^ | 0.995 | 0.994 | 0.999 | 0.998 | 0.998 | - | 0.999 | 0.999 | 0.999 | 0.999 | 0.996 | 0.996 | 0.997 | 0.999 | 0.999 | 0.999 | 0.999 | 0.999 | 0.994 | 0.997 |

Tab. S6. Comparison of adsorption capacity towards As(V), Se(VI), Cu(II), and Cd(II) of the OFM800 material and other activated carbons

| Ions | Adsorbent | S_BET_ [m^2^/g] | Adsorption capacity [mg/g] | pH | Comment | References |
| --- | --- | --- | --- | --- | --- | --- |
| As(V) | CO_2_-activated carbon obtained directly from orange peels at 800℃ | 266 | 4.18 | 6 | calculated from Langmuir-Freundlich isotherm | this study |
|  | AC from banana peduncle, activated with H_3_PO_4_ | 750 | 13.33 | 6 | calculated from Langmuir isotherm | [12] |
|  | AC from banana peduncle, activated with H_3_PO_4_ and oxidized with KMnO_4_ and then impregnated with FeSO_4_·7H_2_O | 370 | 9.066 | 6 | calculated from Langmuir isotherm | [12] |
|  | AC from apricot stone, activated with H_3_PO_4_ and treated with NaOH and HCl | 1574 | 0.034 | 3 | experimental | [13] |
|  | AC from apricot stone, activated with H_3_PO_4_ and treated with NaOH and HCl, then modified with Fe(III) | 1231 | 2.023 | 3 | experimental | [13] |
|  | AC from apricot stone, activated with H_3_PO_4_ and treated with NaOH and HCl, then modified with Fe(II) | 987 | 3.009 | 3 | experimental | [13] |
|  | Ac from peach stones, activated with steam at 800℃ | no data | 0.23 | 4 | experimental | [14] |
| Se(IV) | CO_2_-activated carbon obtained directly from orange peels at 800℃ | 266 | 2.58 | 6 | calculated from Langmuir-Freundlich isotherm | this study |
|  | commercial AC Norit RB3 impregnated with Fe(II) and sodium acetate | 1063 | 0.028 | 7 | calculated from Langmuir isotherm | [15] |
|  | commercial lignite coal AC Darco S51 activated by steam | 650 | 0.23 | 7 | calculated from Langmuir isotherm | [16] |
|  | commercial lignite coal AC Darco S51 activated by steam, impregnated with Cu(II) | 531 | 0.68 | 7 | calculated from Langmuir isotherm | [16] |
|  | waste wood AC, activated with phosphoric acid and functionalized with iron salts | no data | 11.41 | 7.5 | experimental | [17] |
|  | iron-coated granular AC coated with Fe(II) | 436 | 2.58 | 5 | calculated from Langmuir isotherm | [18] |
| Cu(II) | CO_2_-activated carbon obtained directly from orange peels at 800℃ | 266 | 36.86 | 6 | calculated from Langmuir-Freundlich isotherm | [19] |
|  | AC from *Cieba pentandra hulls* activated with steam | 521 | 20.8 | 6 | calculated from Langmuir isotherm | [20] |
|  | Orange peels prapared at 500℃ activated with H_2_O_2_ | 96 | 38.9 | 5 | calculated from Langmuir isotherm | [21] |
|  | AC from orange peels modified by Titanium dioxide nanoparticles | 6 | 13.34 | 5 | experimental | [22] |
|  | AC from date seeds modified by Titanium dioxide nanoparticles | 81 | 13.96 | 5 | experimental | [22] |
|  | AC from banana leaves, activated with phosphoric acid | no data | 66.2 | 5 | calculated from Langmuir isotherm | [23] |
| Cd(II) | CO_2_-activated carbon obtained directly from orange peels at 800℃ | 266 | 39.43 | 6 | calculated from Langmuir-Freundlich isotherm | this study |
|  | AC from *Cieba pentandra hulls* activated with steam | 521 | 19.5 | 6 | calculated from Langmuir isotherm | [19] |
|  | AC from *Mauritia flexuosa* activated chemically through phosphoric acid at 600℃ | 906 | 26.33 | 5 | calculated from Langmuir isotherm | [24] |
|  | AC from *Olea europaea* L. fruit stones activated chemically through phosphoric acid at 600℃ | 1169 | 24.83 | 5 | calculated from Langmuir isotherm | [24] |
|  | AC from plum stones activated with phosphoric acid | 829 | 112.74 | 6 | calculated from Langmuir isotherm | [25] |
|  | Orange peels prapared at 500℃ activated with H_2_O_2_ | 96 | 26.88 | 5 | calculated from Langmuir isotherm | [26] |
| EPS | CO_2_-activated carbon obtained directly from orange peels at 800℃ | 266 | 16.14 | 6 | experimental for initial conc. 100 mg/L | this study |
|  |  |  | 20.75 | 5 |  |  |
|  | sunflower husk biochar, prepared at 500℃ | 7 | 2.42 | 5 | experimental for initial conc. 50 mg/L | [27] |
| CtPAM | CO_2_-activated carbon obtained directly from orange peels at 800℃ | 266 | 26.47 | 6 | experimental for initial conc. 100 mg/L | this study |
|  |  |  | 39.95 | 7 |  |  |
|  | AC from hay, activated with orthophosphoric acid in microwave furnance at 550℃ | 271 | 32.5 | 6 | experimental for initial conc. 100 mg/L | [28] |
| AnPAM | CO_2_-activated carbon obtained directly from orange peels at 800℃ | 266 | 10.37 | 6 | experimental for initial conc. 100 mg/L | this study |
|  |  |  | 12.41 | 5 |  |  |
|  | AC from hay, activated with orthophosphoric acid in microwave furnance at 550℃ | 271 | 11.6 | 6 | experimental for initial conc. 100 mg/L | [28] |


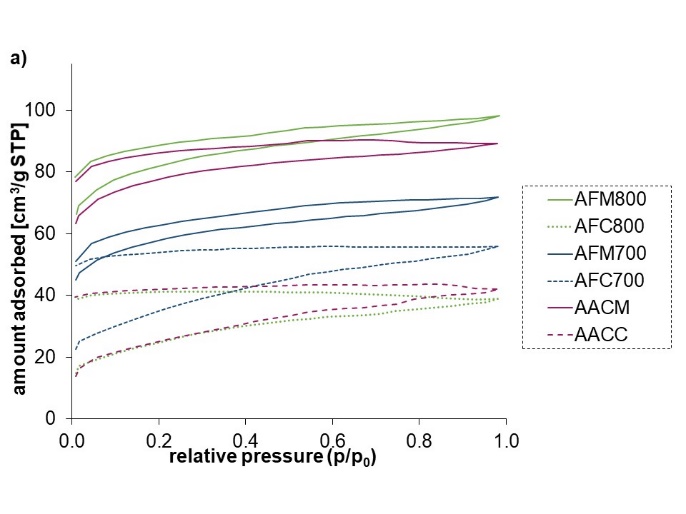

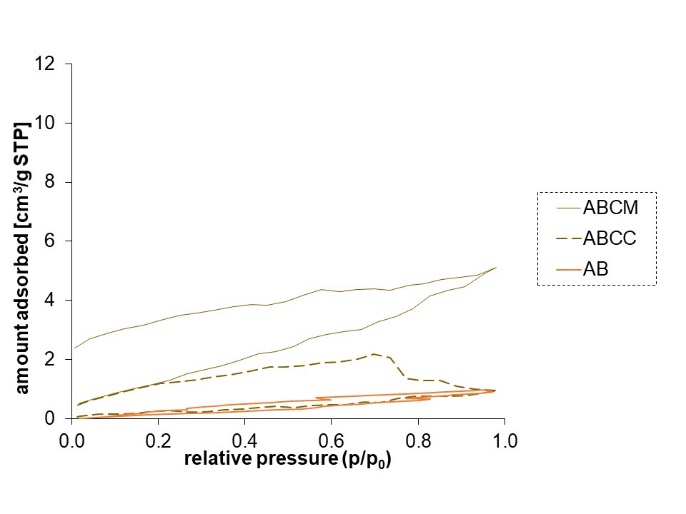

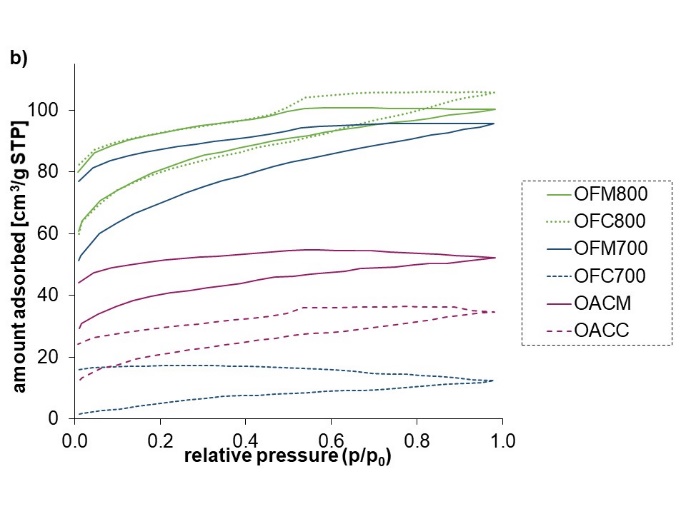

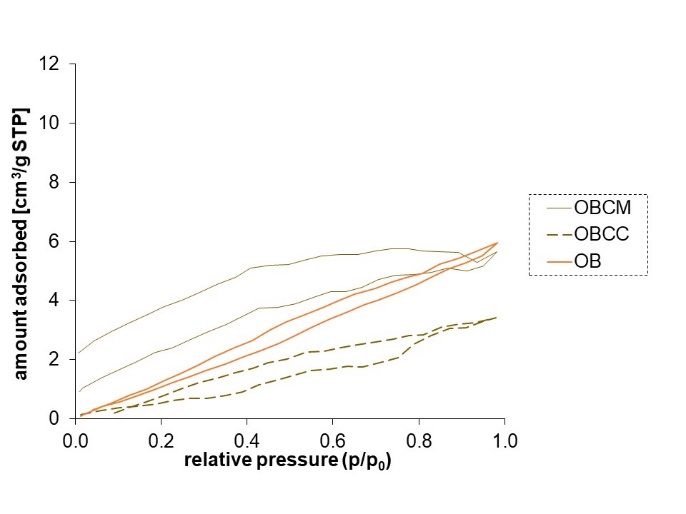

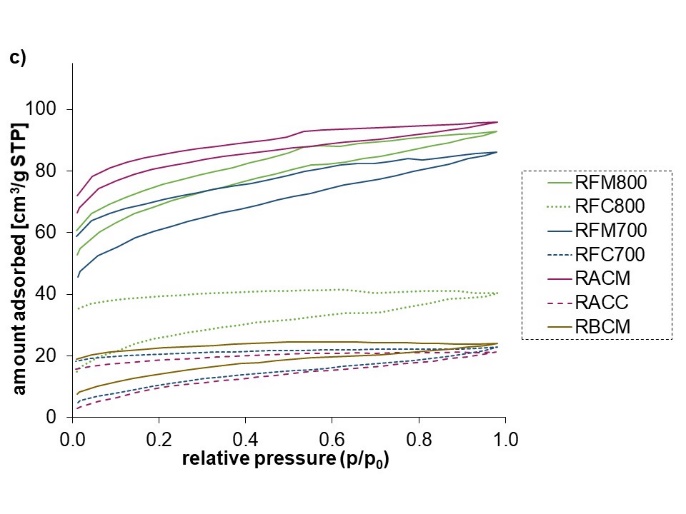

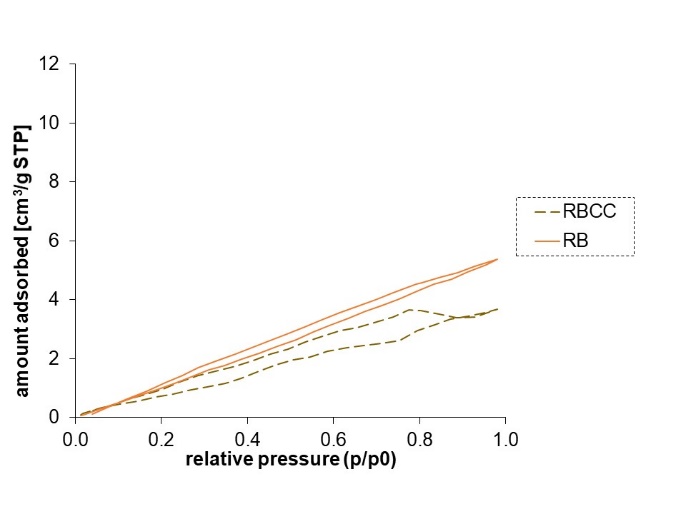


Fig. S1. Nitrogen adsorption/desorption isotherms for chokeberry seeds (a), orange peels (b), and black currant seeds (c) and biochars/activated carbons produced from them


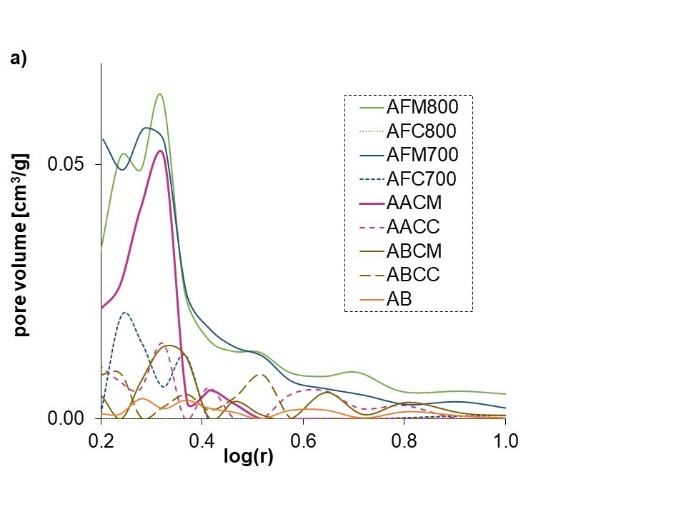

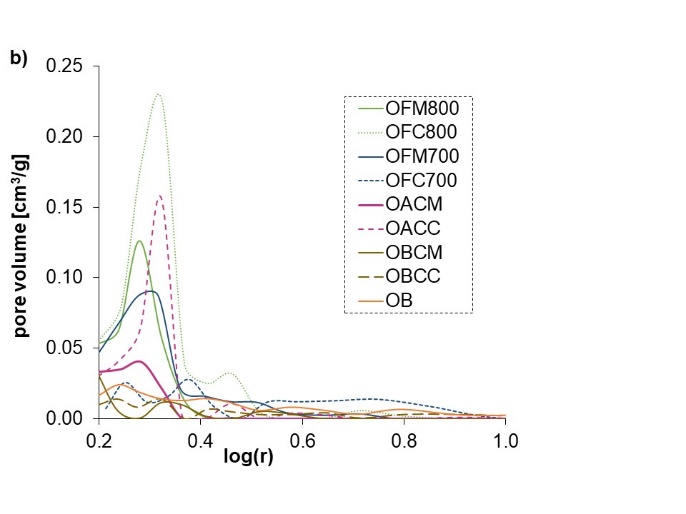

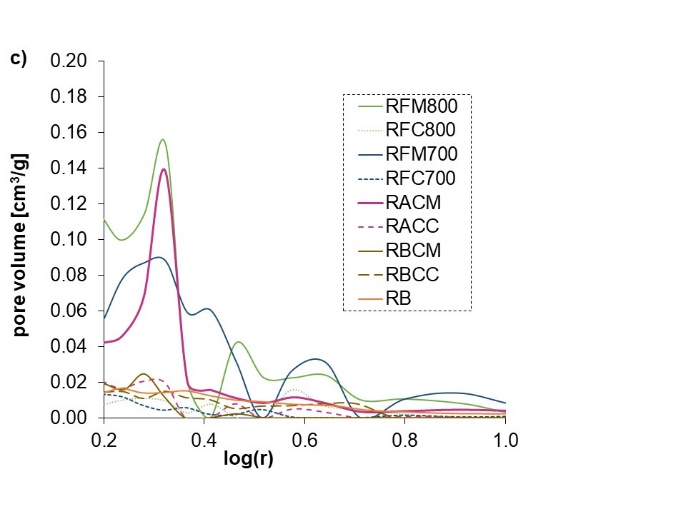


Fig. S2. Pore size distribution for chokeberry seeds (a), orange peels (b), and black currant seeds (c) and biochars/activated carbons produced from them


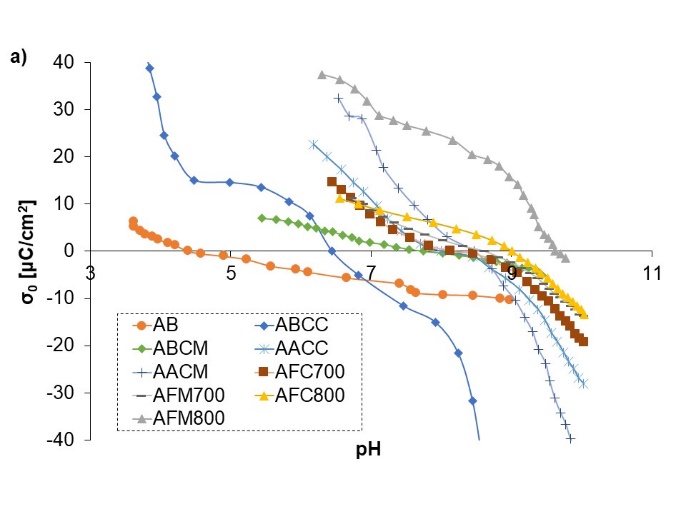

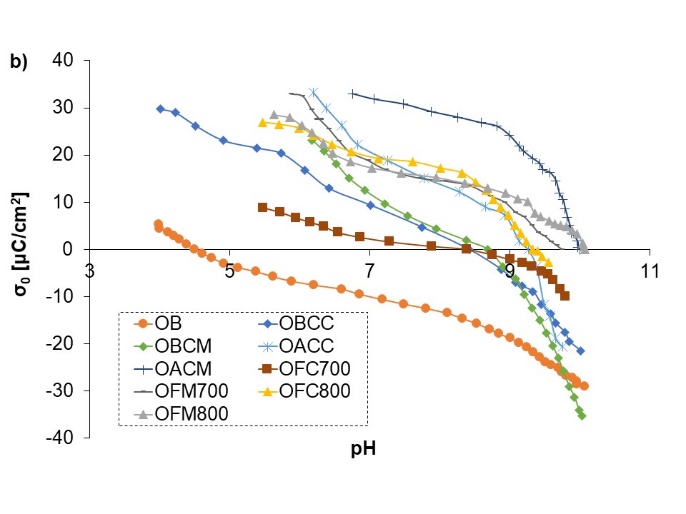


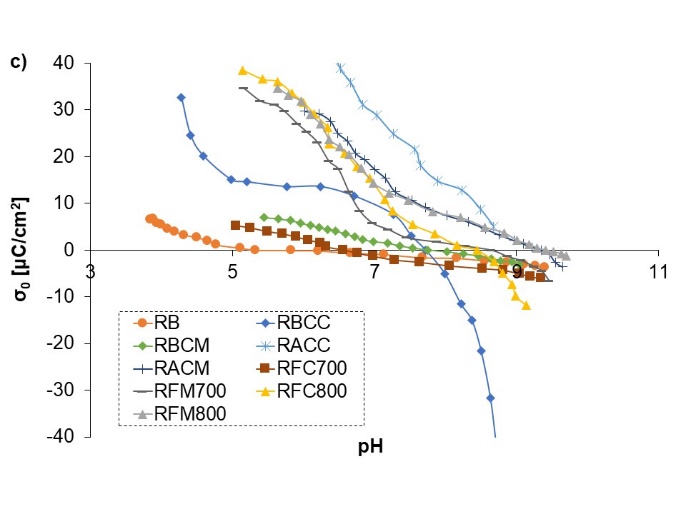


Fig. S3. Surface charge density as a function of pH value for chokeberry seeds (a), orange peels (b), black currant seeds (c), and biochars/activated carbons prepared from them


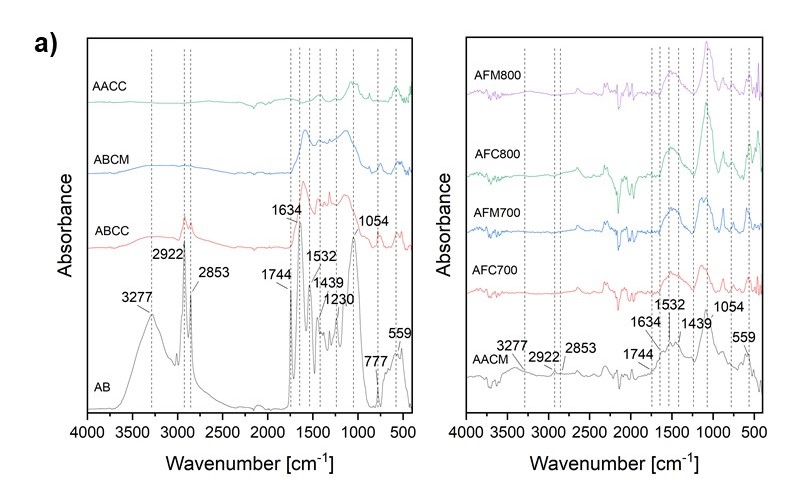

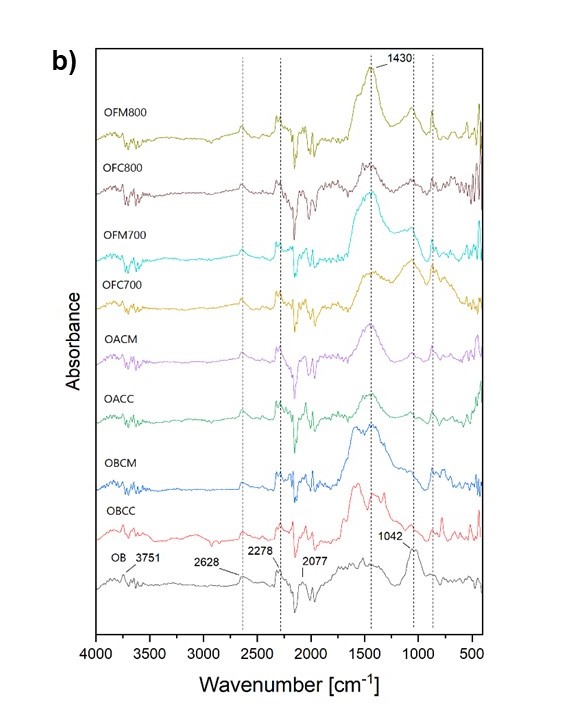

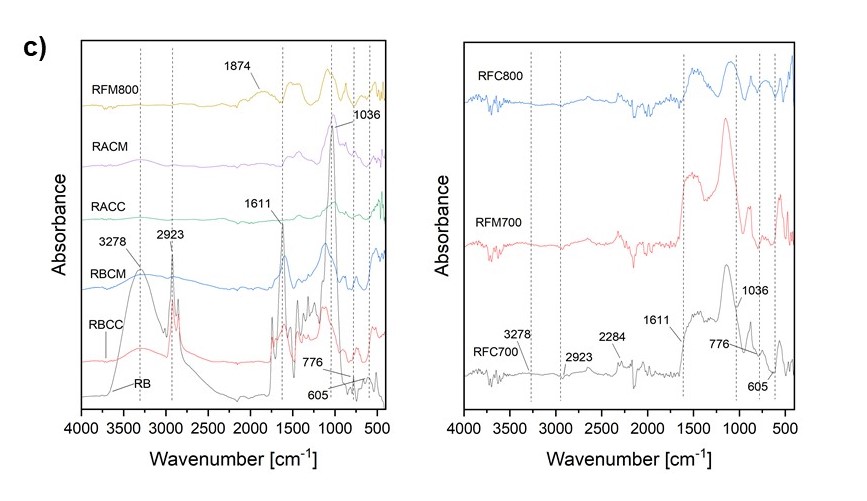


Fig. S4. FTIR spectra of chokeberry seeds (a), orange peels (b), black currant seeds (c), as well as biochars/activated carbons produced from them


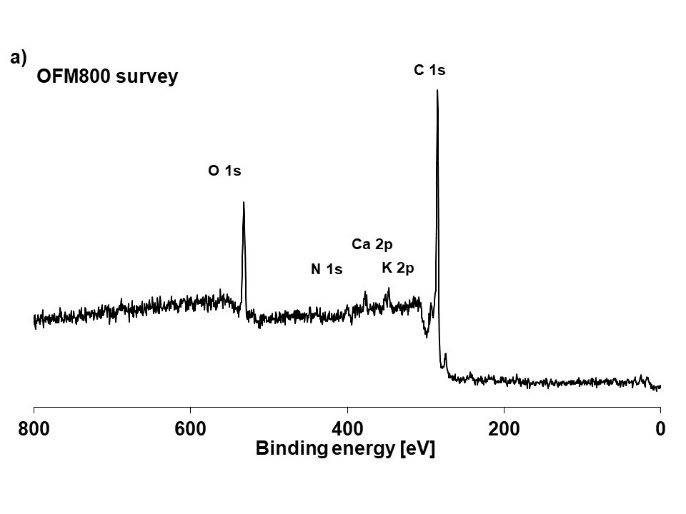

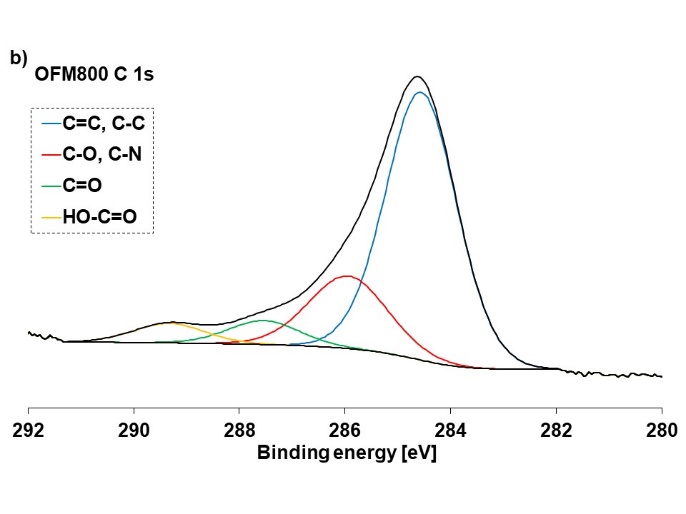

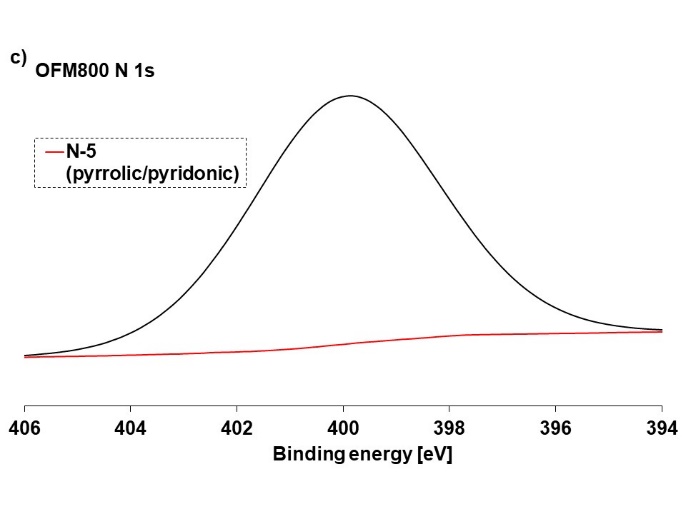

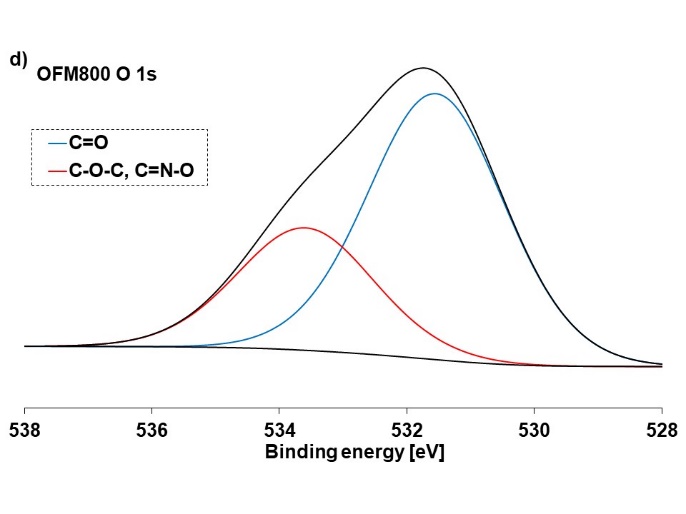


Fig. S5. XPS spectra of the OFM800 material: (a) survey, (b) C1s, (c) N1s, (d) O1s


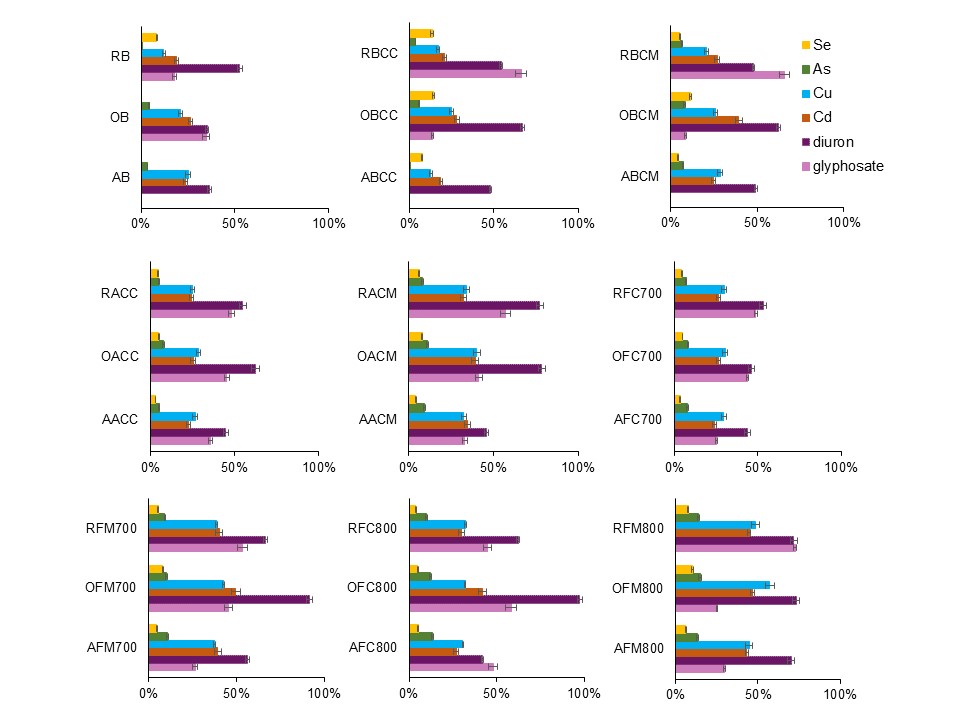


Fig. S6. Adsorbed amounts of metals, metalloids, and herbicides on chokeberry seeds (a), orange peels (b), black currant seeds (c), as well as biochars/activated carbons produced from them (the initial Cd/Cu/As/Se concentration was 100 mg/L, whereas the DCMU/GLY one, 10 mg/L)


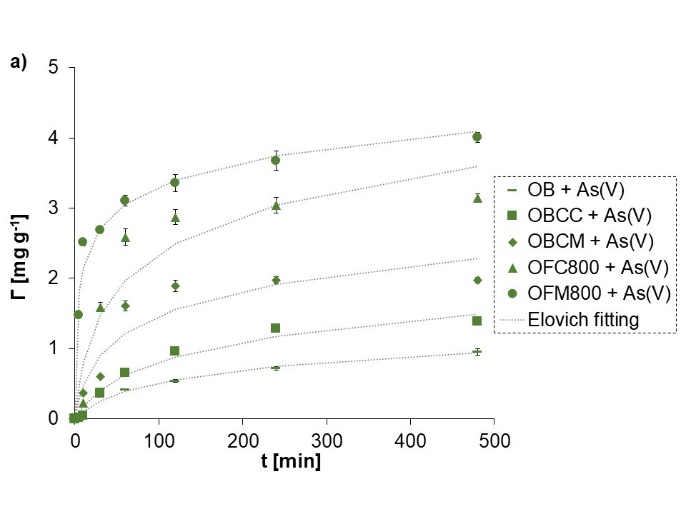

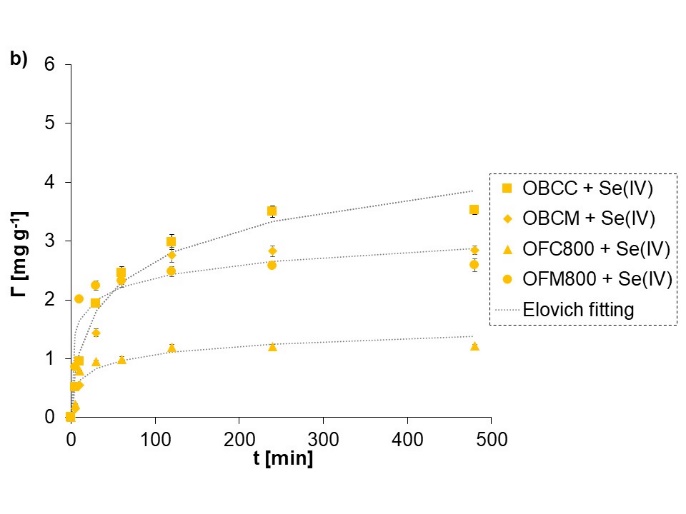

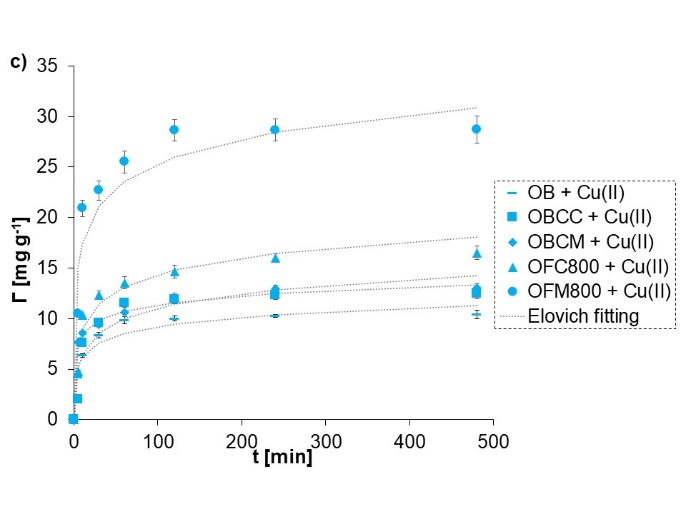

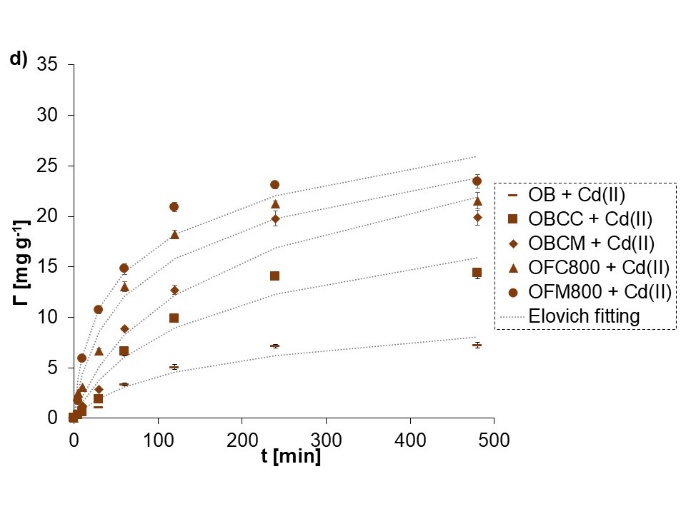


Fig. S7. Experimental adsorption kinetics of metal and metalloids: As(V) (a), Se(IV) (b), Cu(II) (c), and Cd(II) (d) on orange peels and orange peels-derived materials fitted to the Elovich model


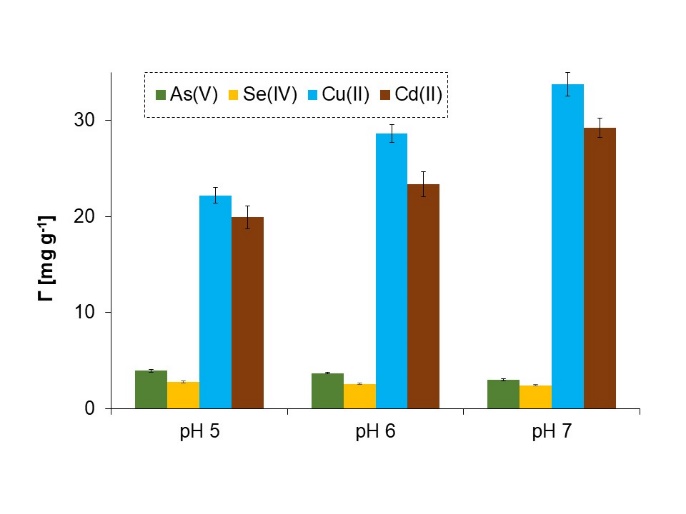


Fig. S8. Adsorbed amounts of metals and metalloids on the OFM800 material at various pH values


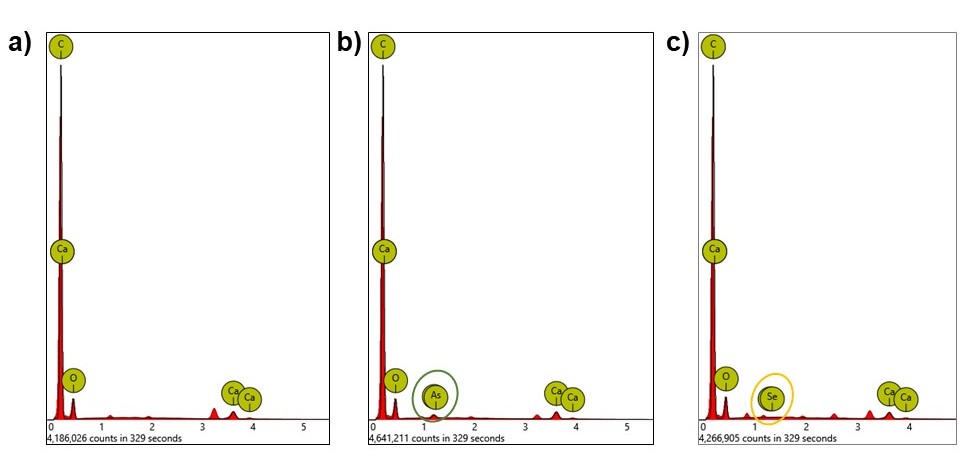


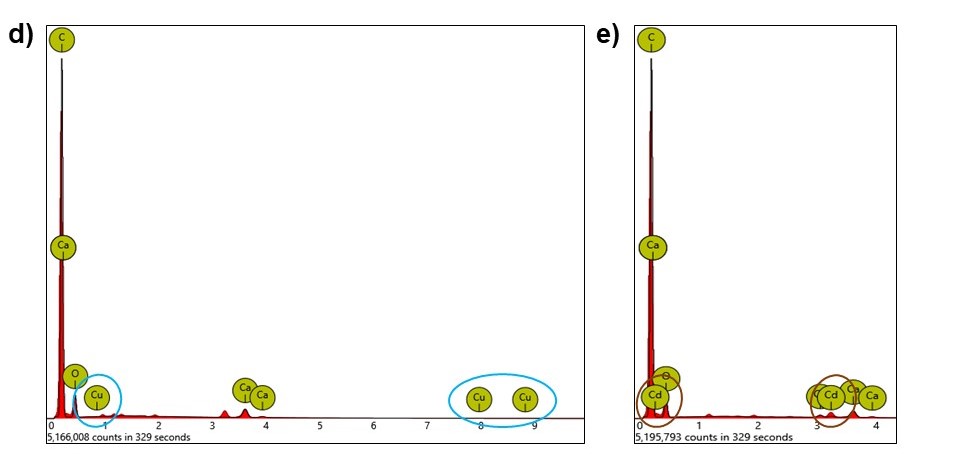


Fig. S9. EDS of the OFM800 material before (a) and after the adsorption of metalloids: As(V) (b), Se(IV) (c), and metals: Cu(II) (d), Cd(II) (e)

a)
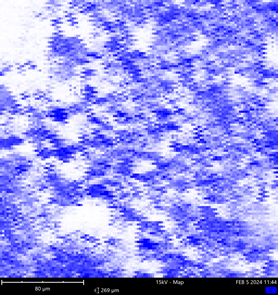

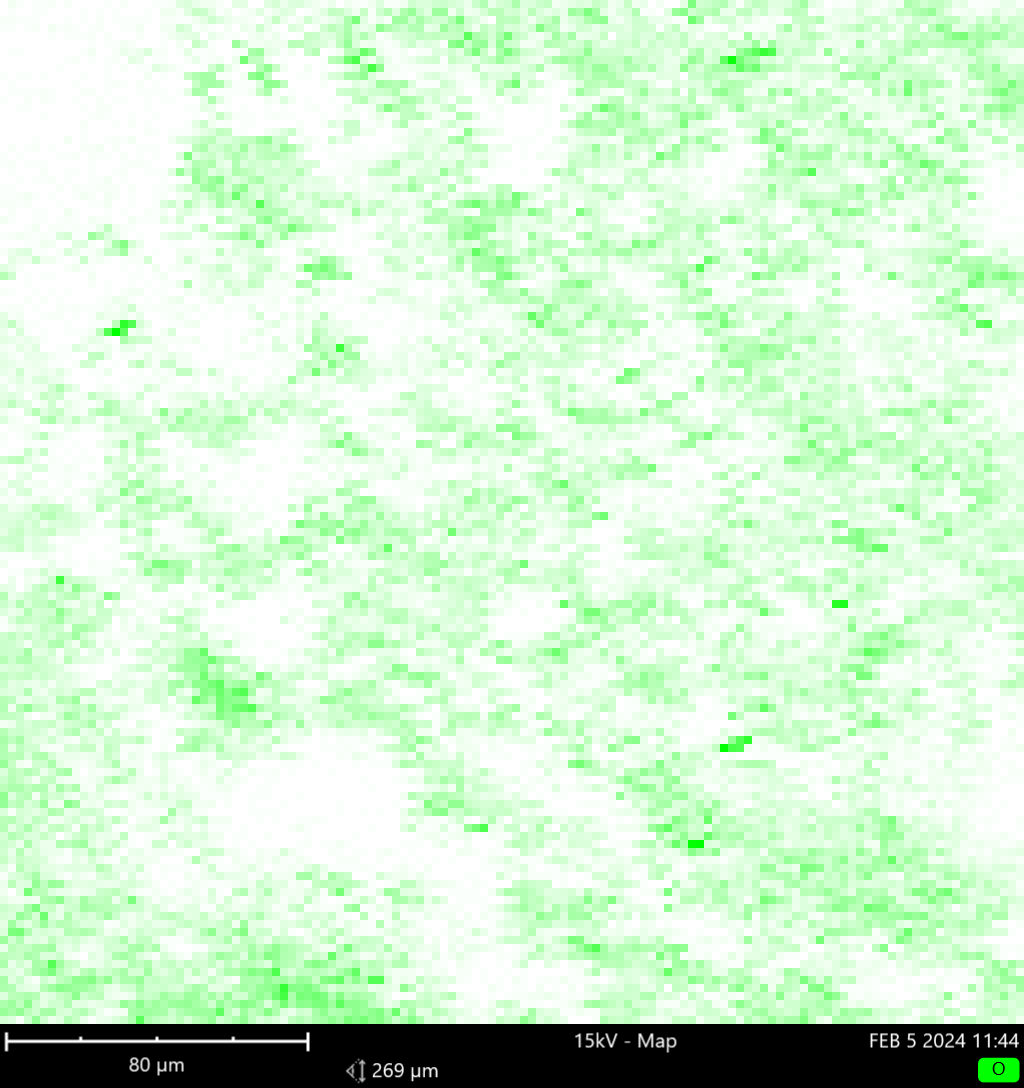

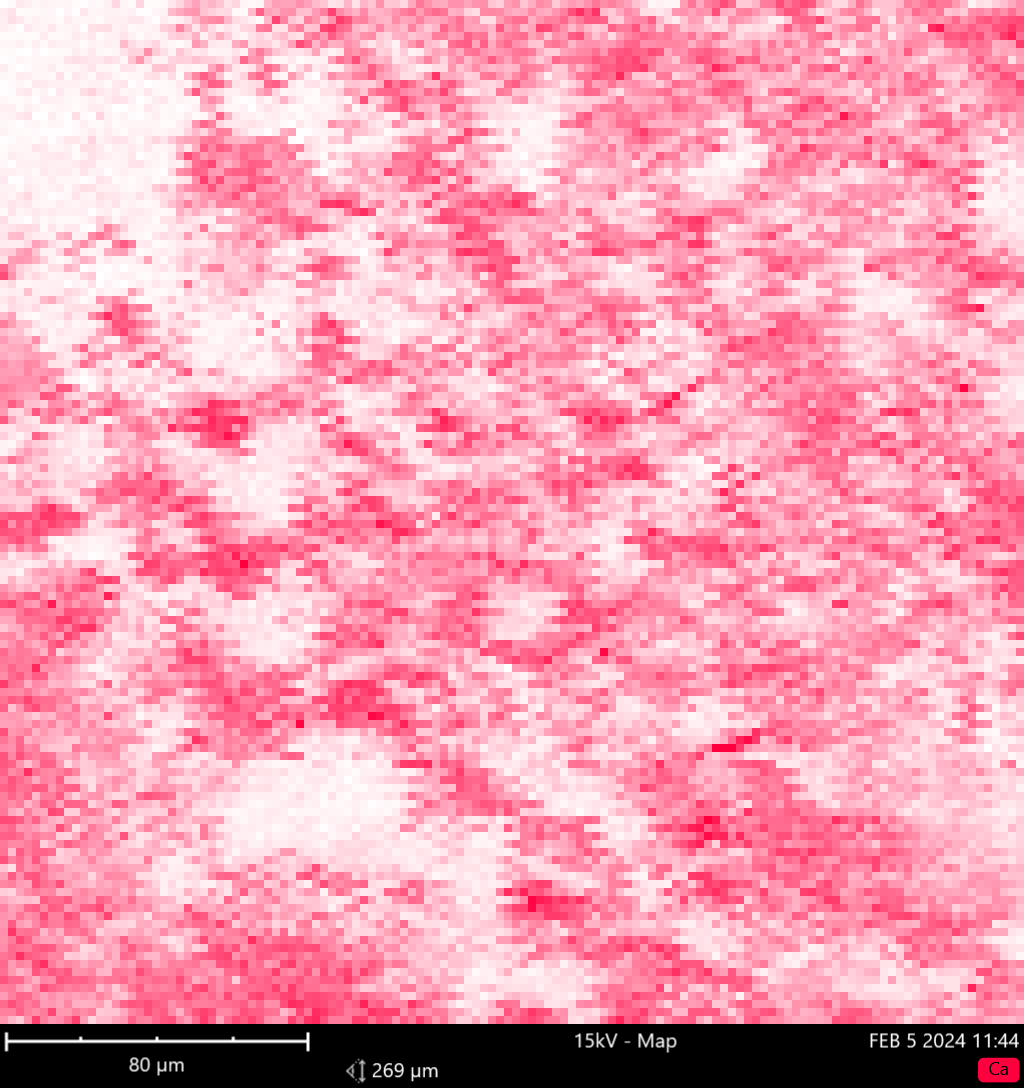


**Ca**

**O**

**C**

b)
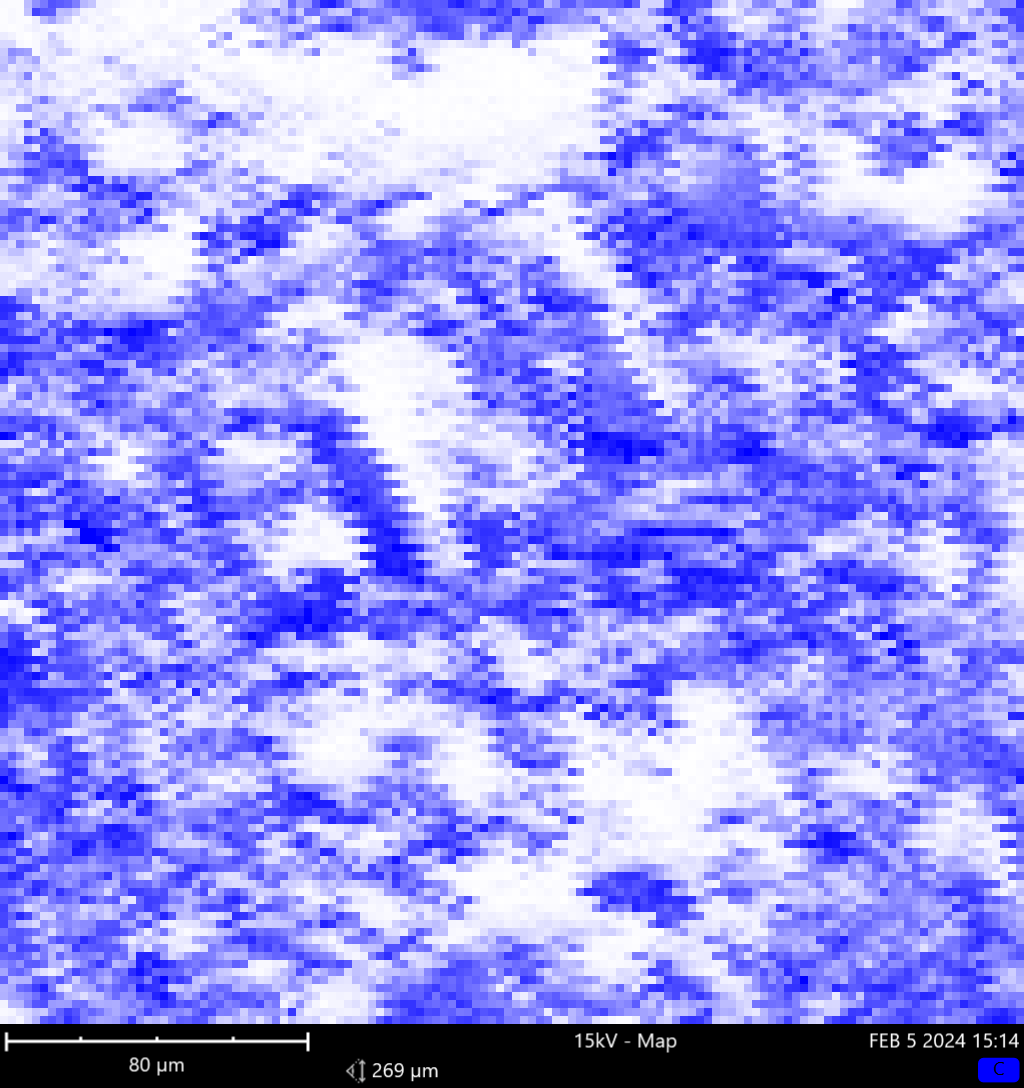

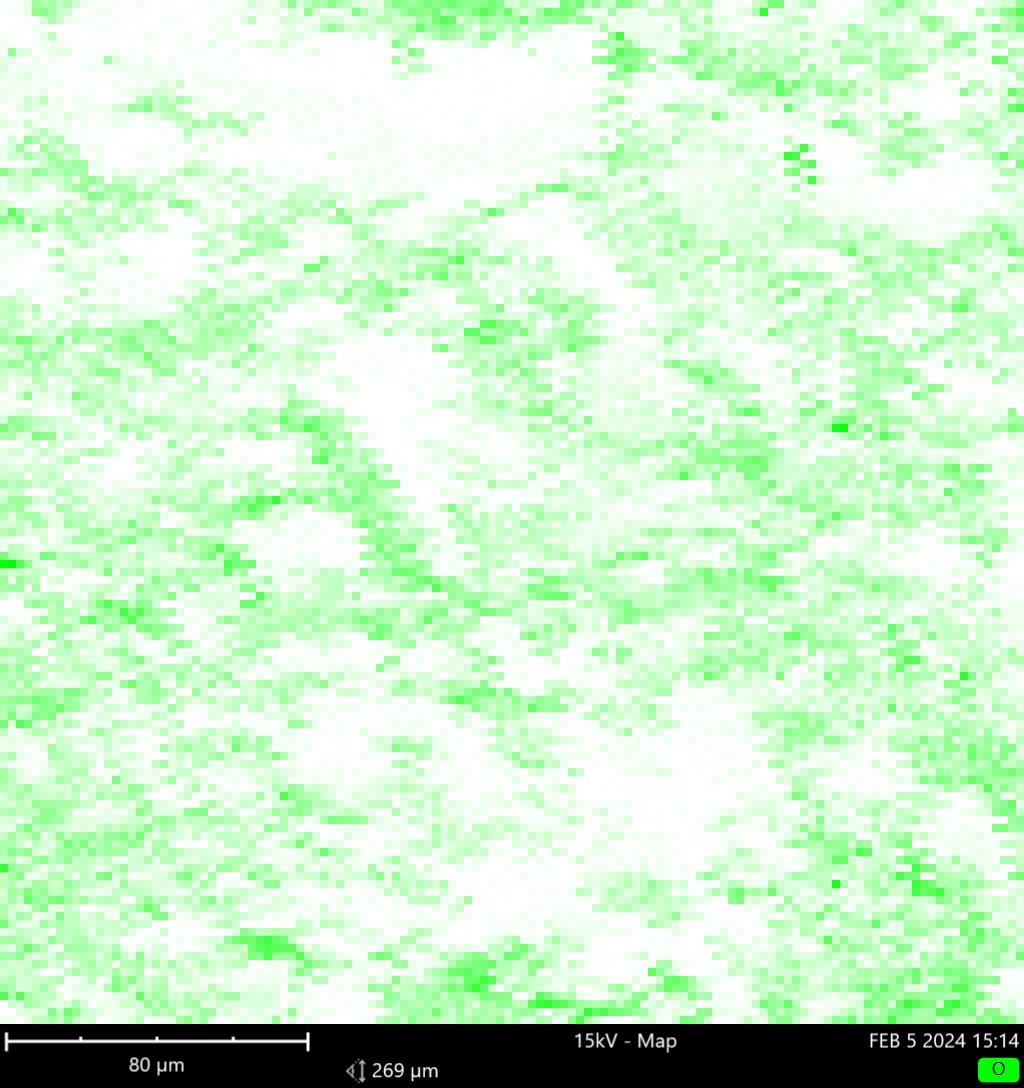

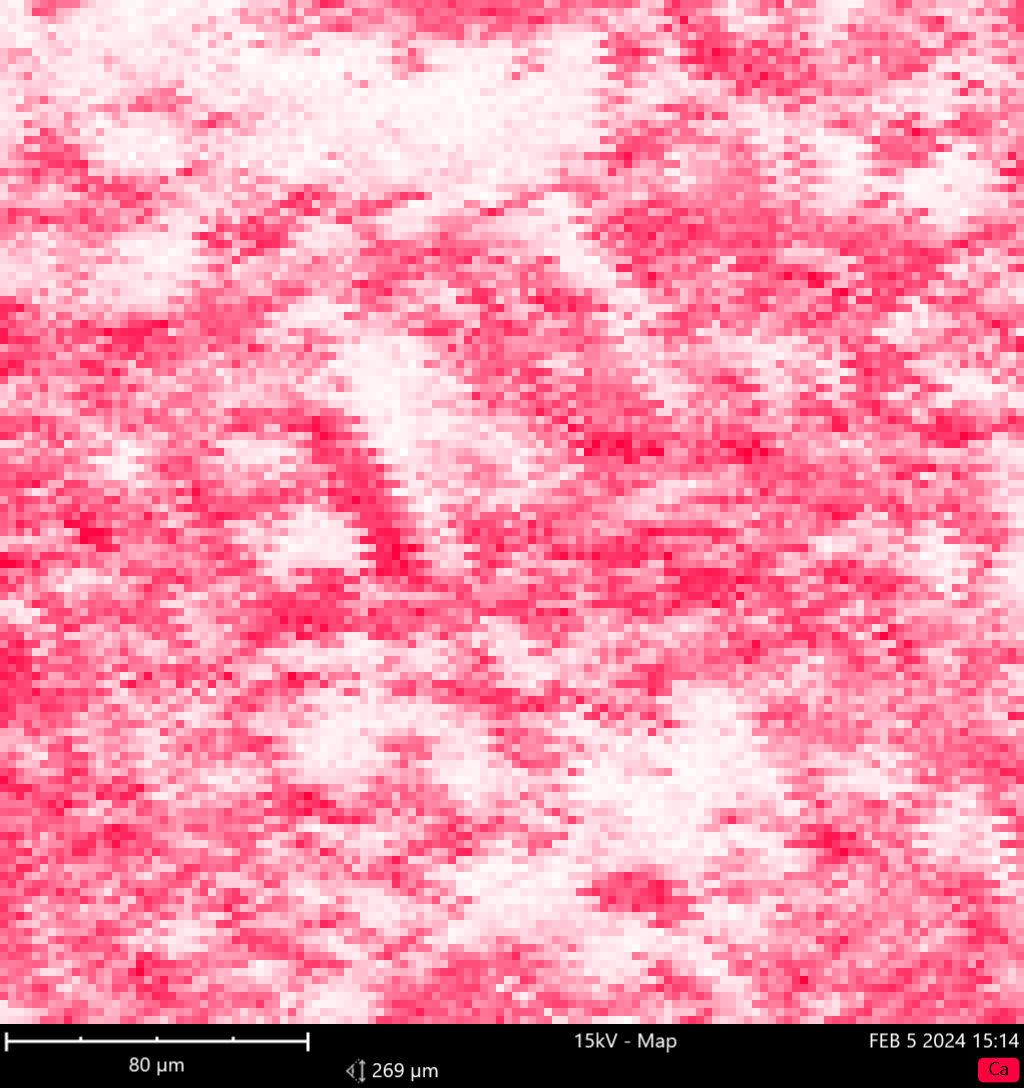

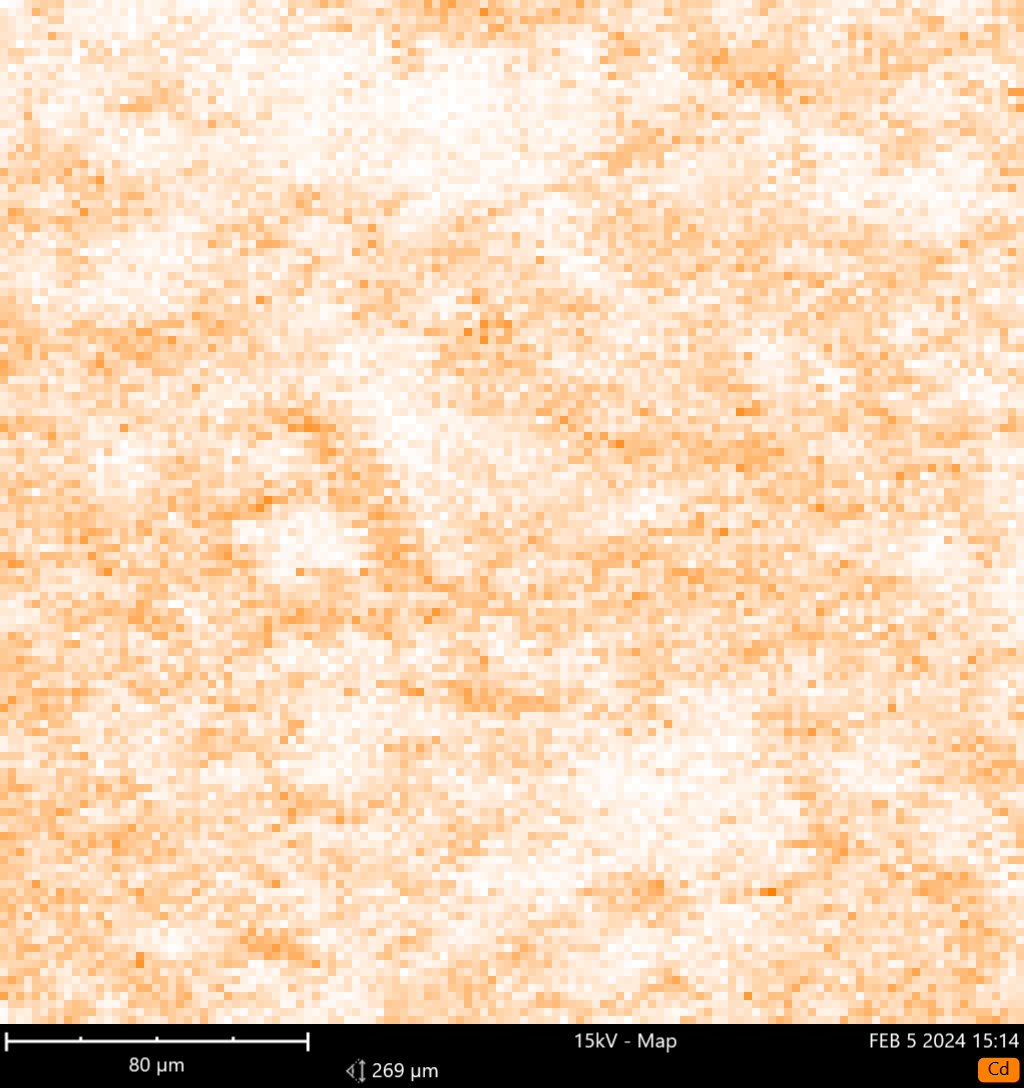


**Cd**

**Ca**

**O**

**C**

c)
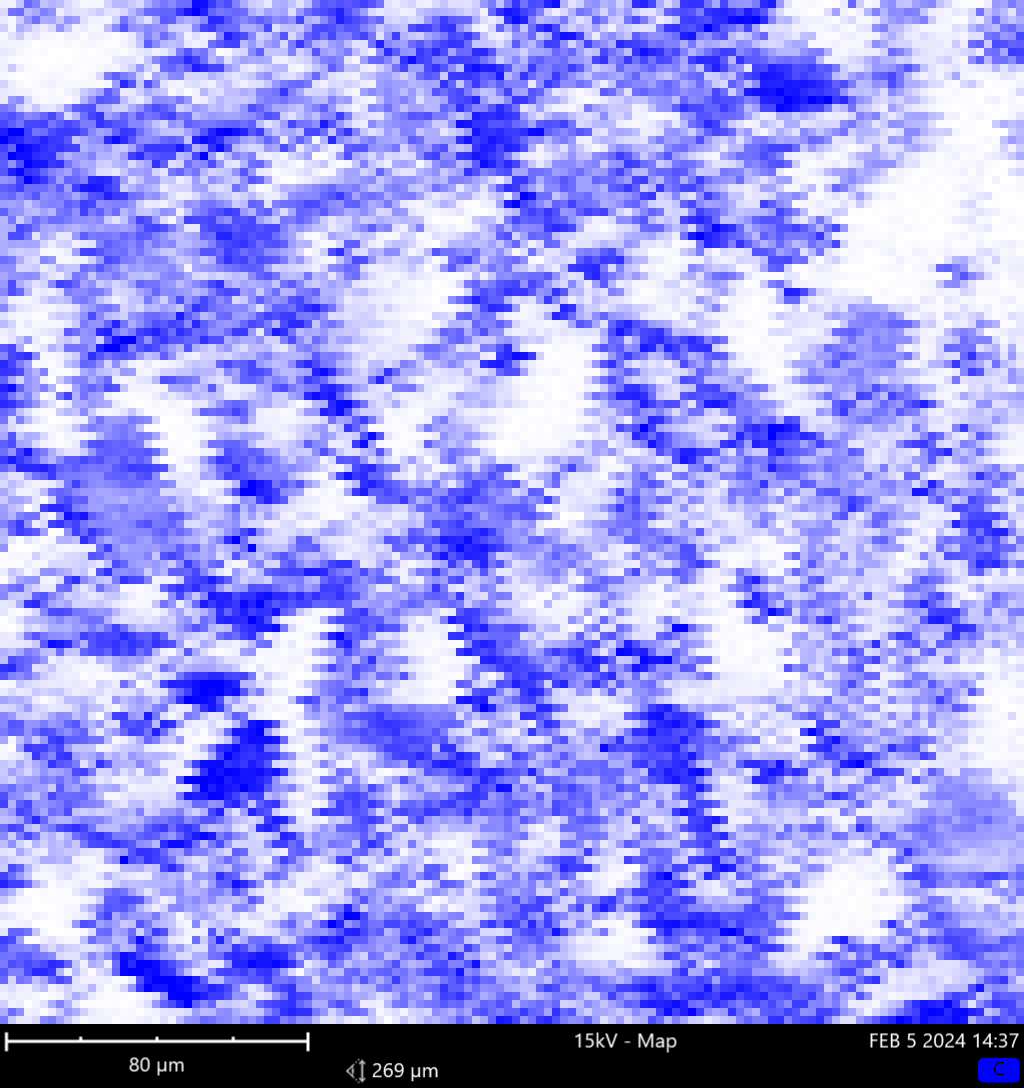

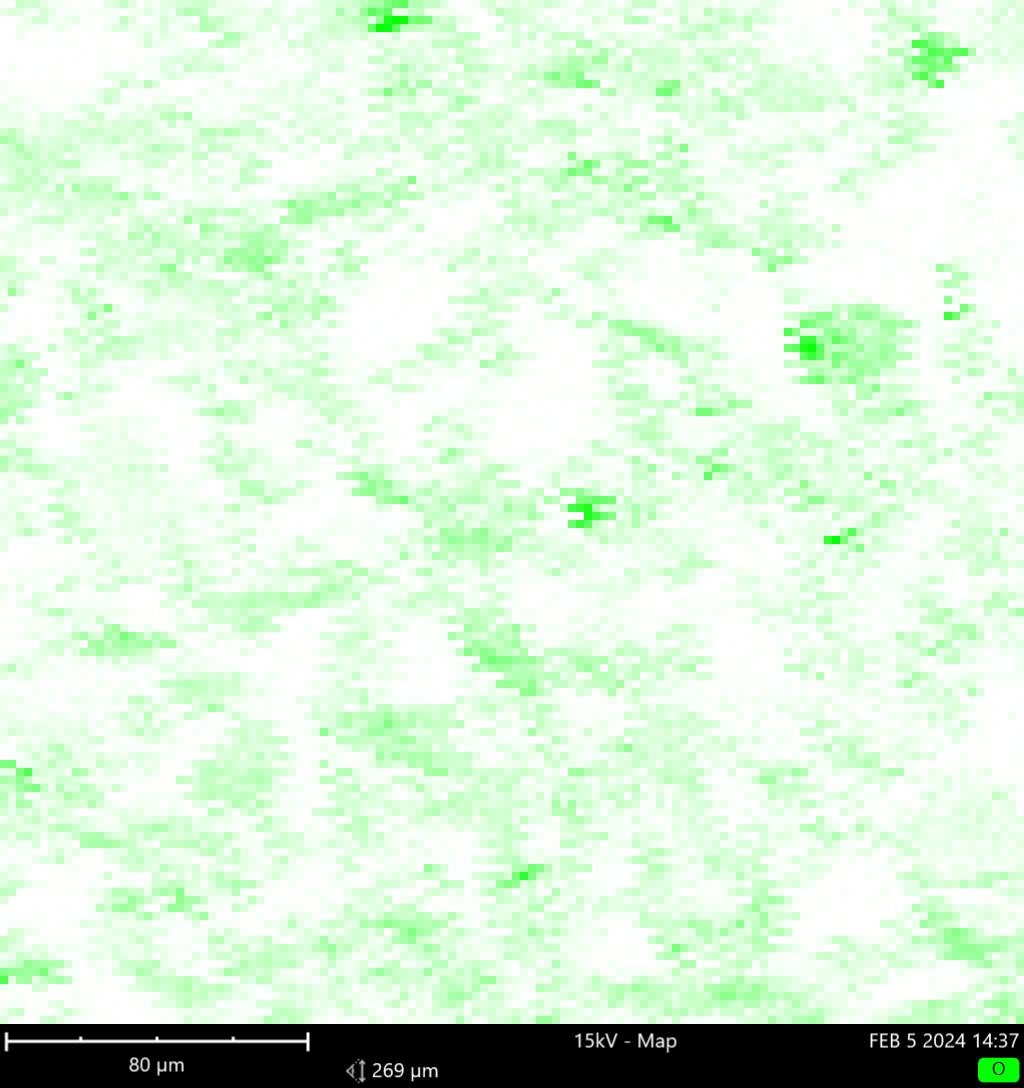

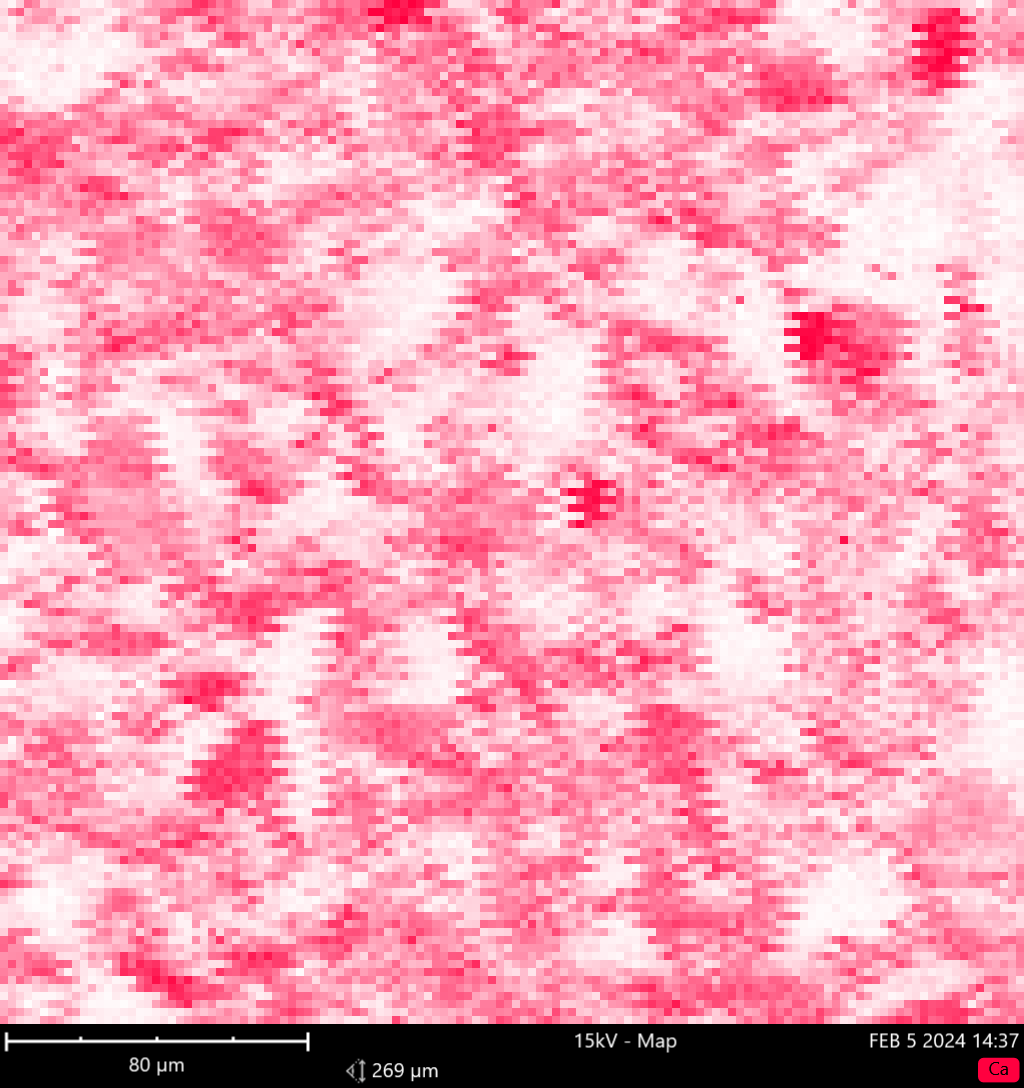

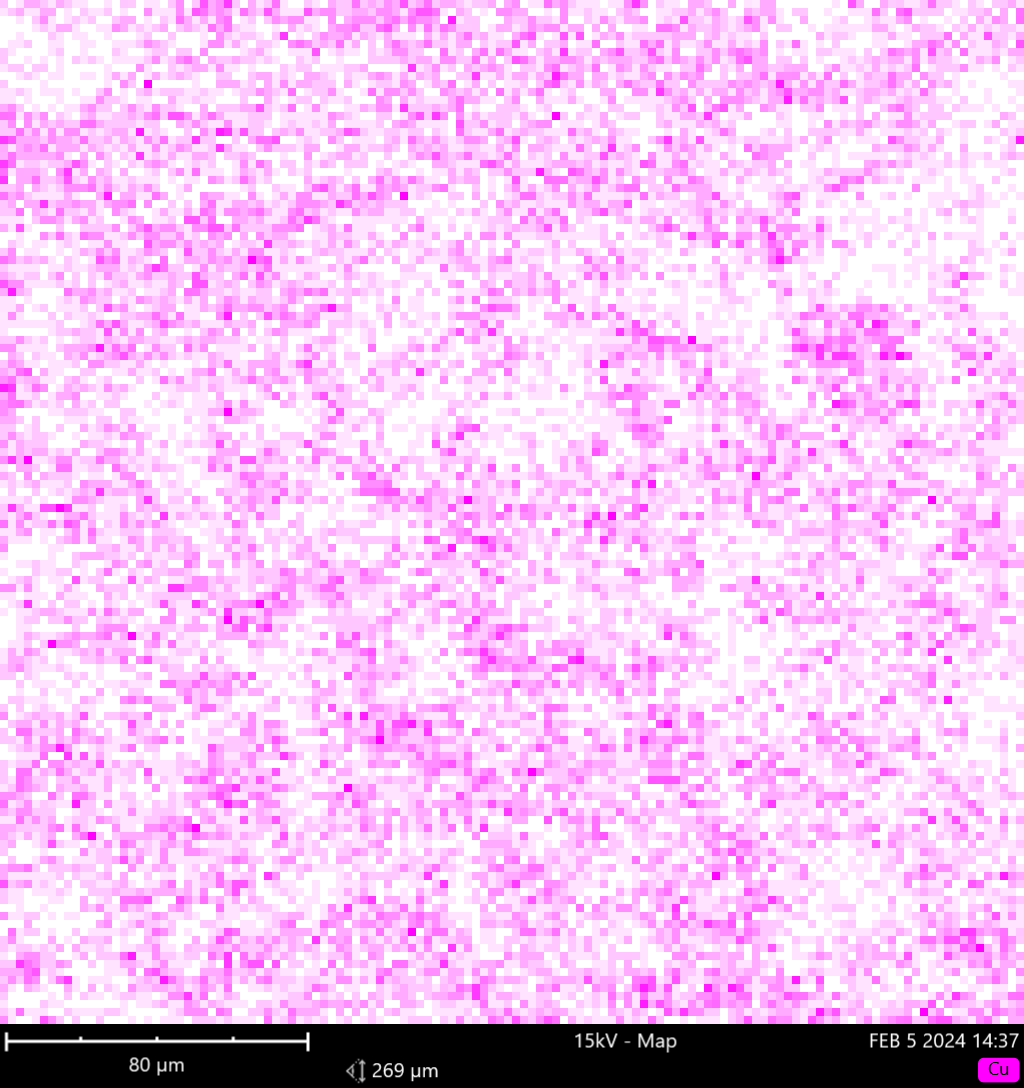


**Cu**

**Ca**

**O**

**C**

d)
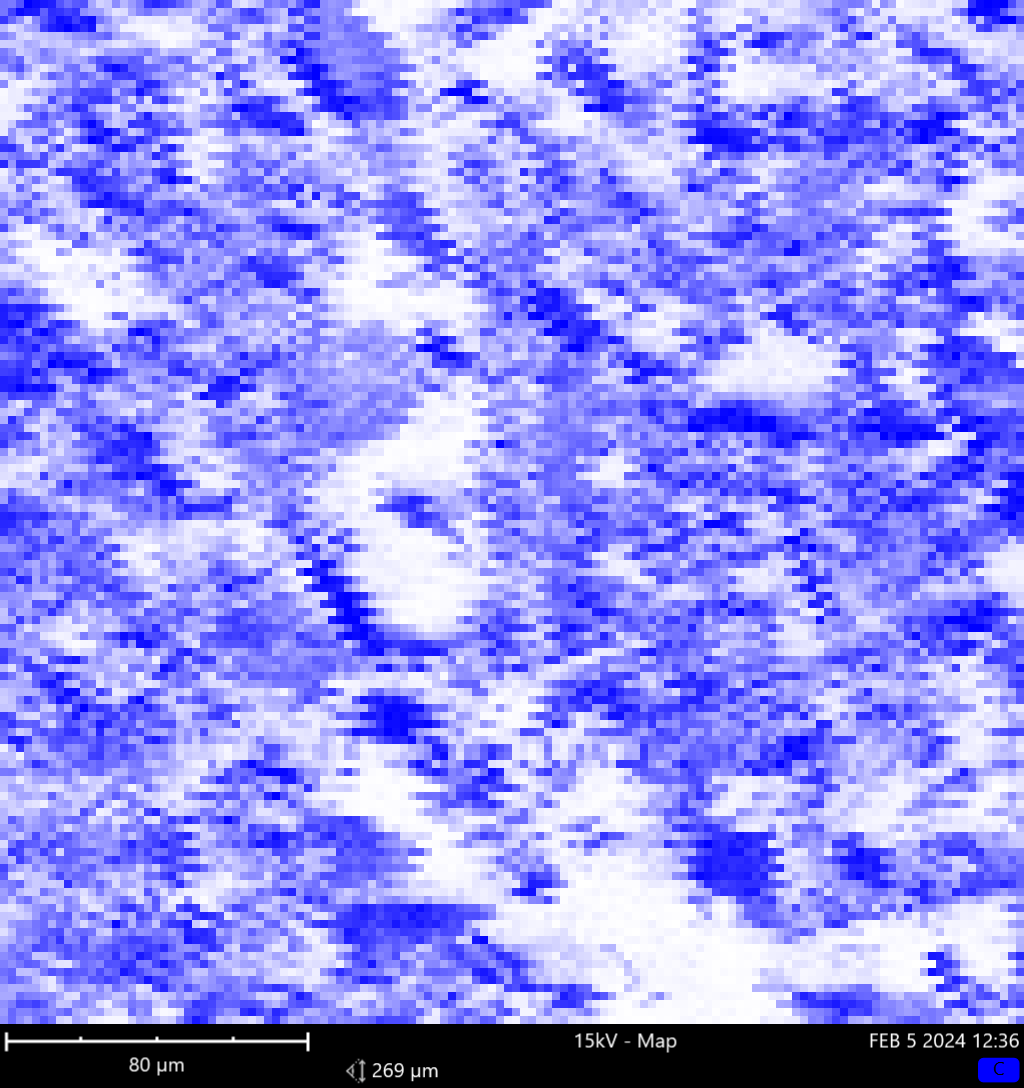

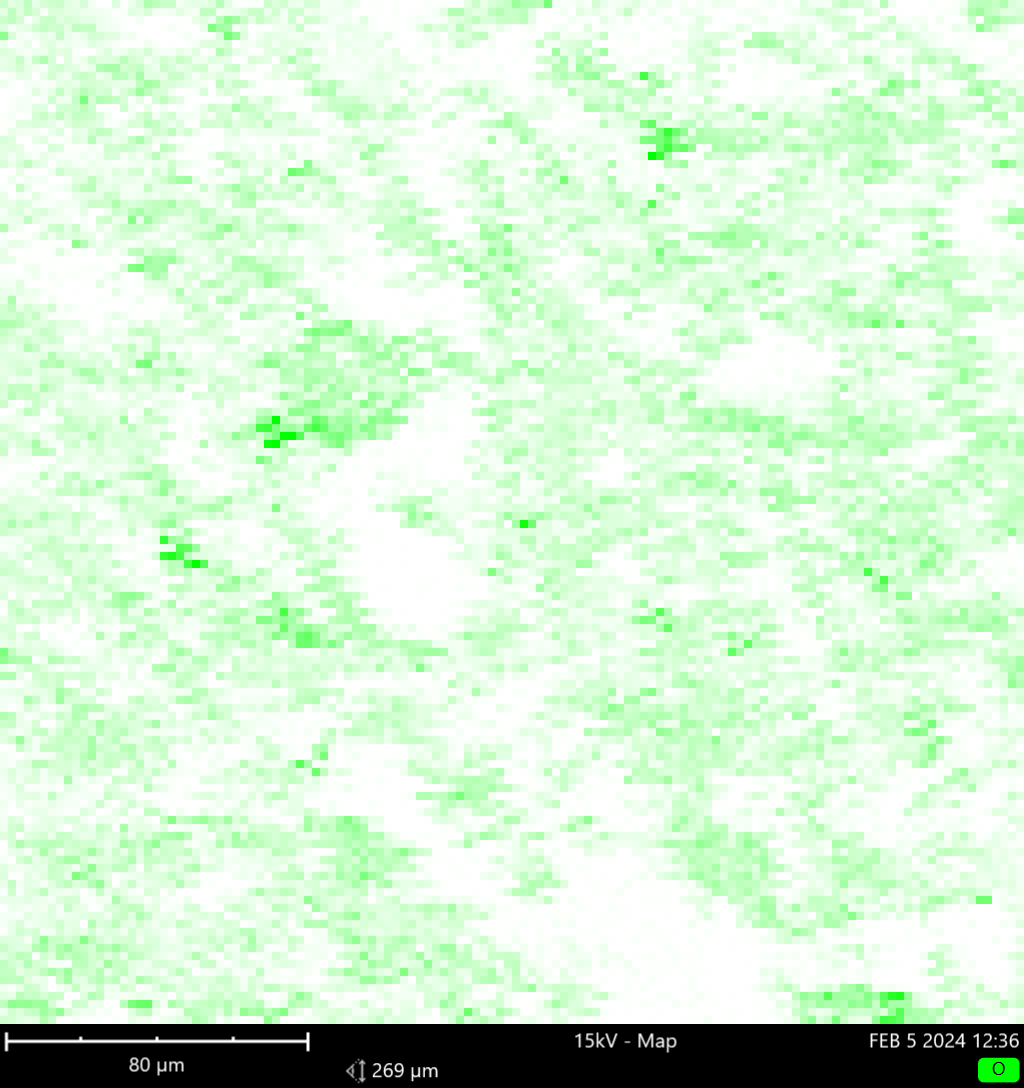

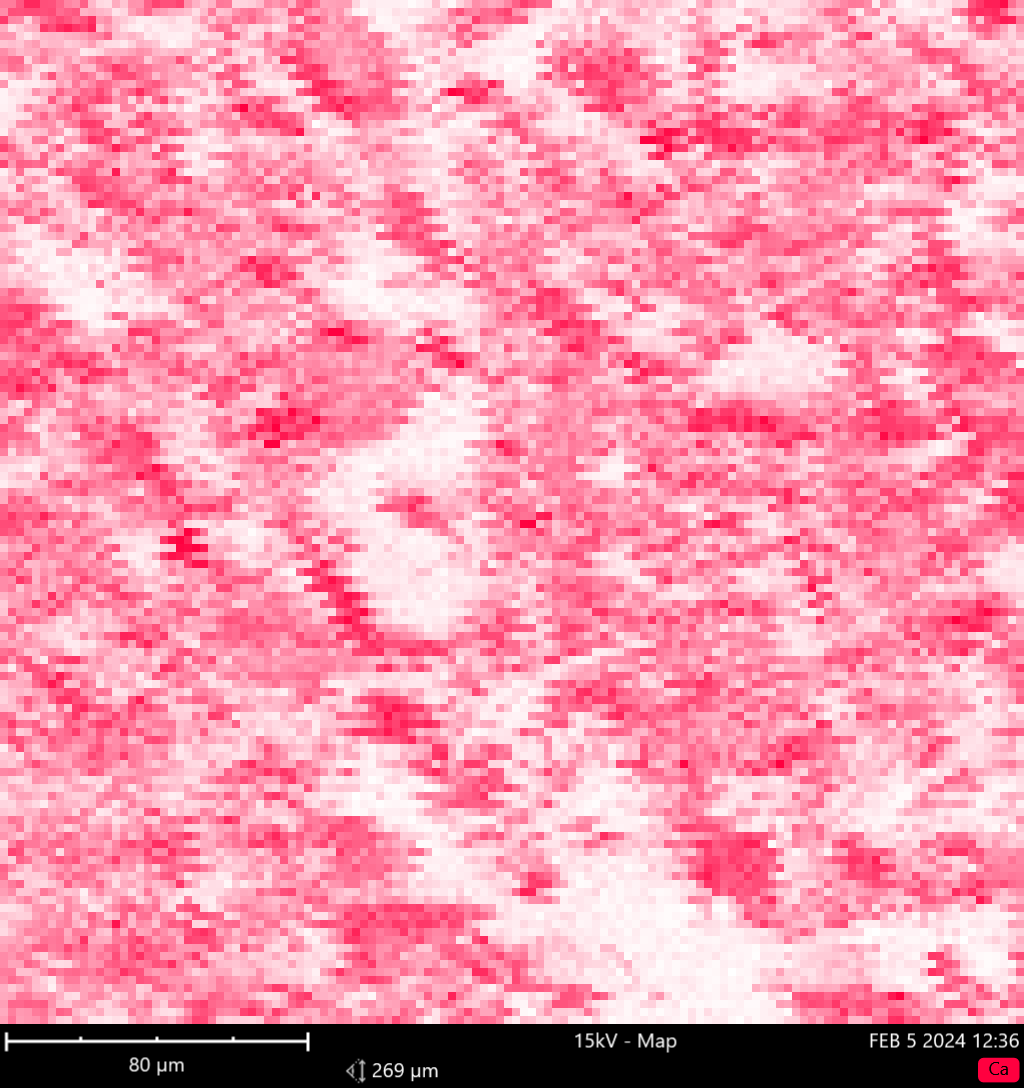

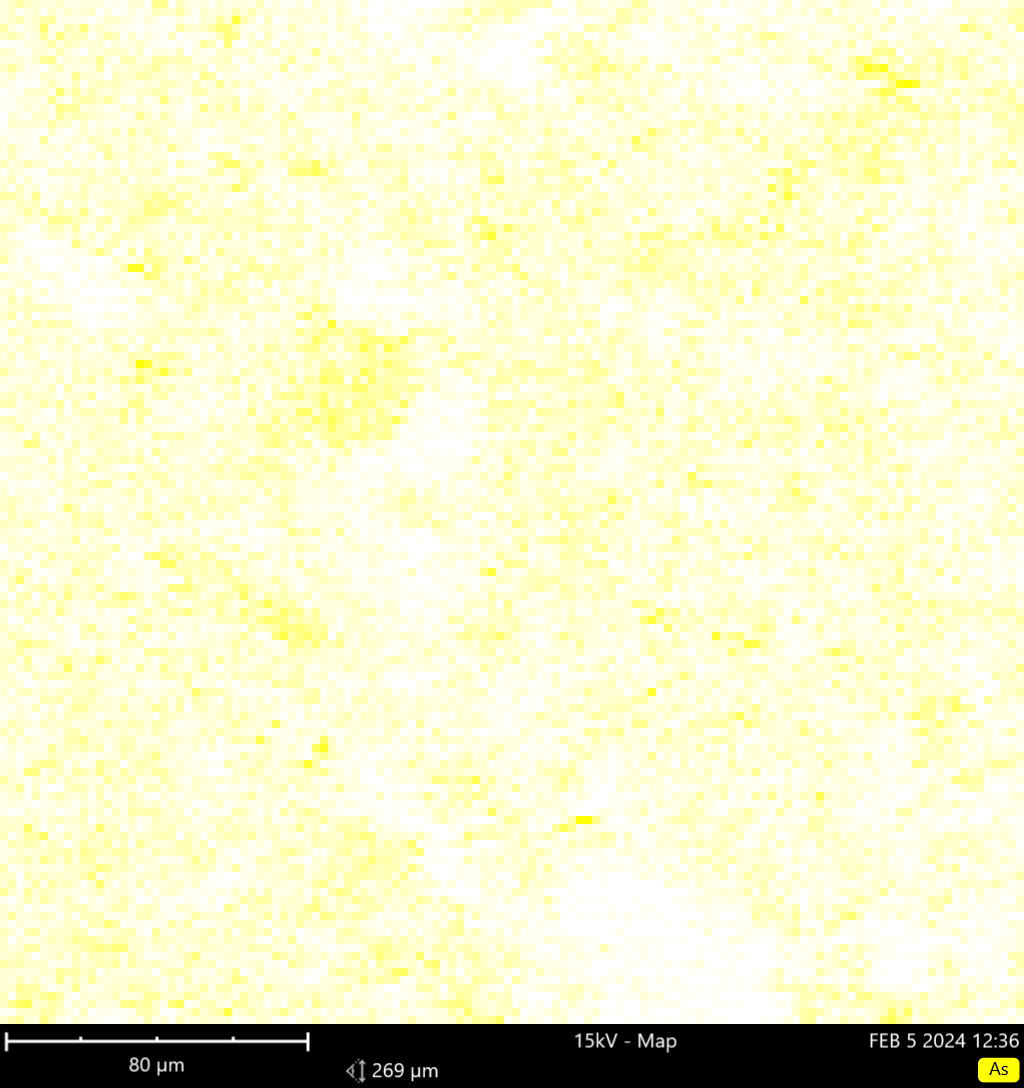


**Ca**

**As**

**C**

**O**

e)
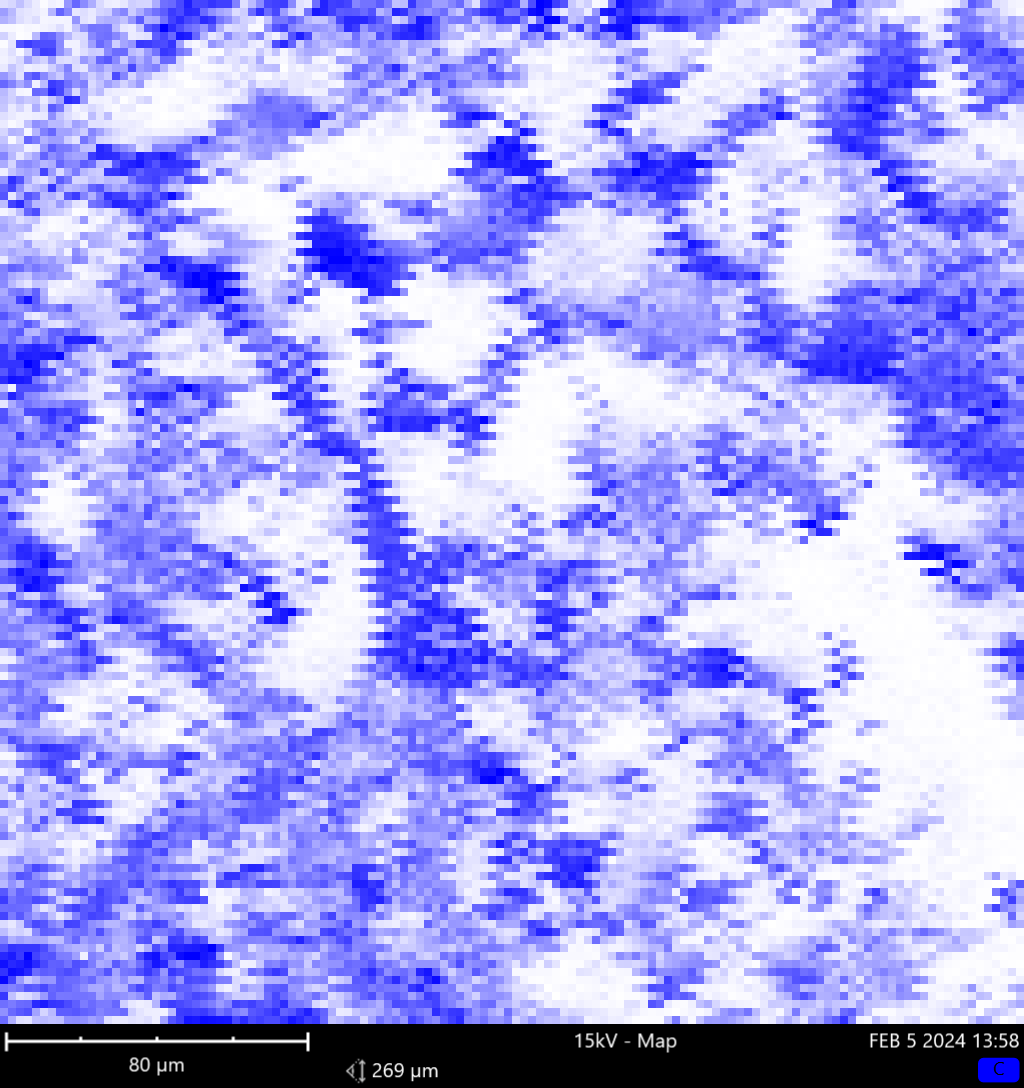

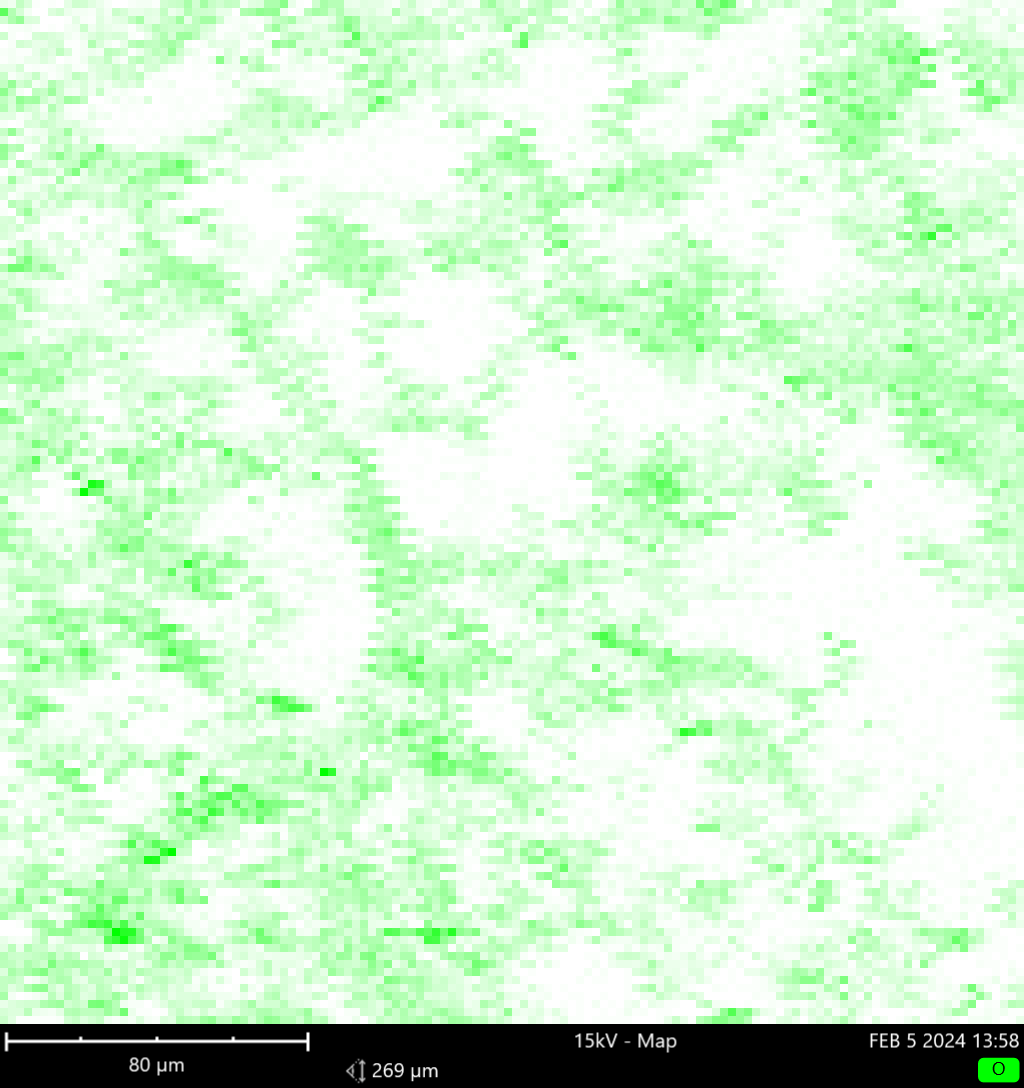

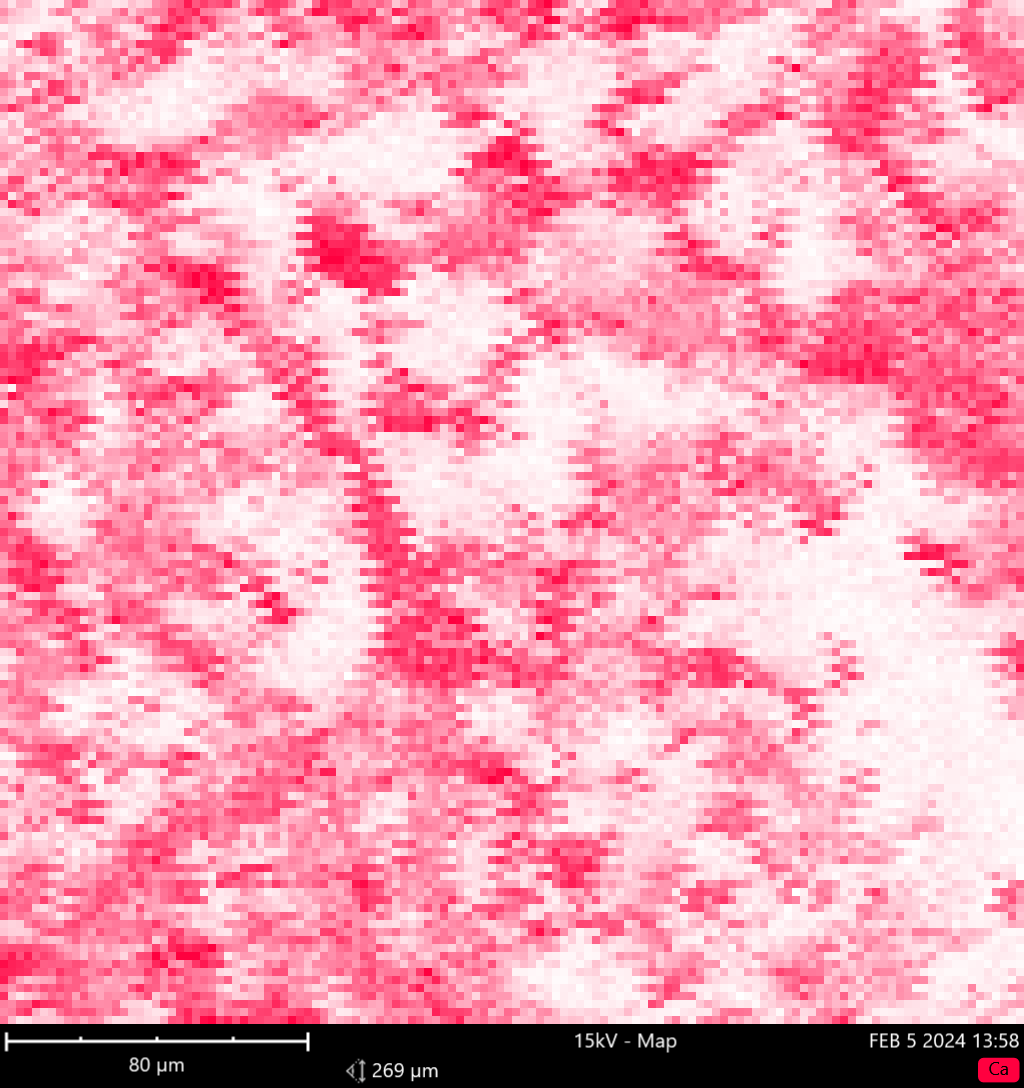

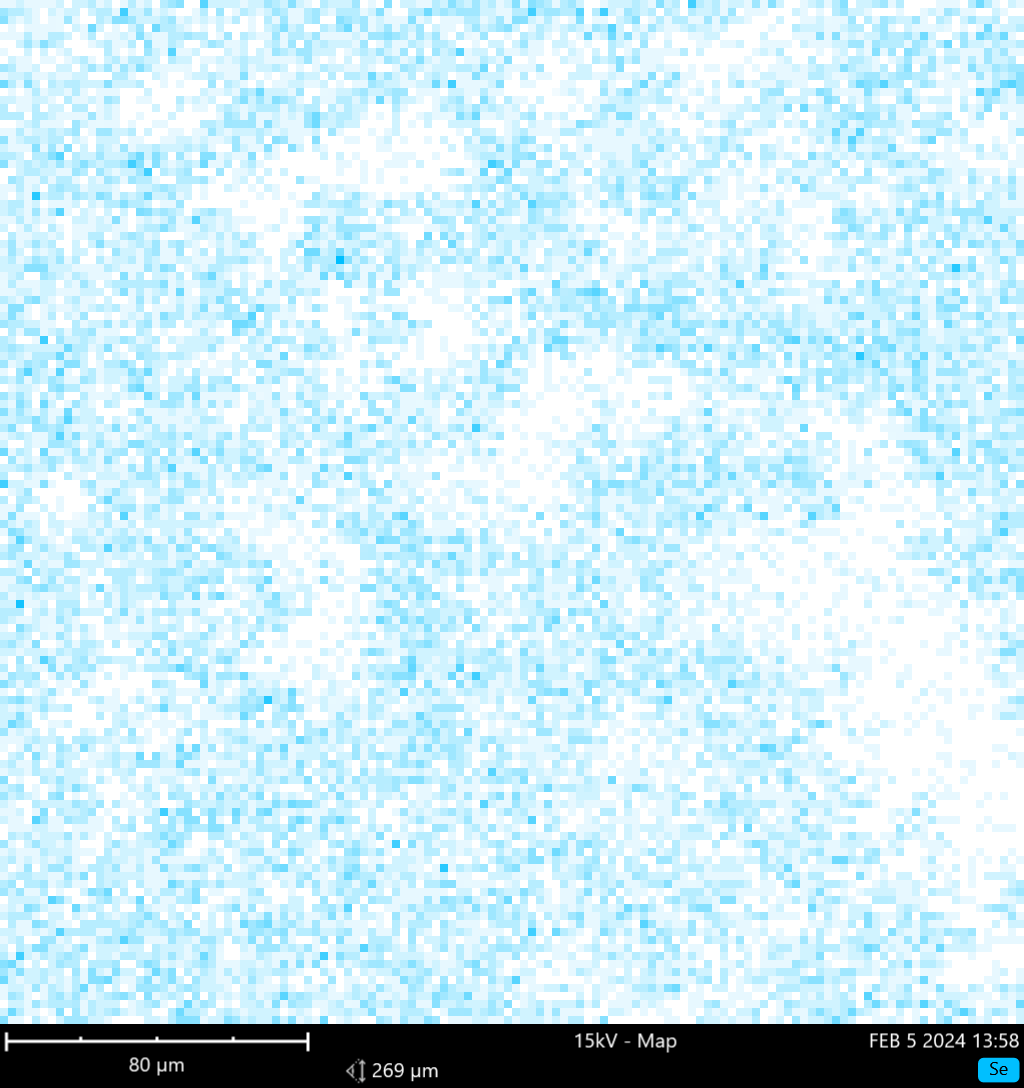


**Ca**

**Se**

**O**

**C**

Fig. S10. SEM-EDS maps for the OFM800 material before (a) and after the adsorption of: Cd(II) (b), Cu(II) (c), As(V) (d), and Se(IV) (e) ions (C – carbon, O – oxygen, Ca – calcium)

**References**

[1] Skorupska, A., Janczarek, M., Marczak, M., Mazur, A. & Król, J. Rhizobial exopolysaccharides: genetic control and symbiotic functions. *Microb. Cell Fact.* **5**, (2006).

[2] Estrada, D., Echeverry, L., Ramirez, A. & Gutierrez, L. Molybdenite Flotation in the Presence of a Polyacrylamide of Low Anionicity Subjected to Different Conditions of Mechanical Shearing. *Minerals* **10**, 895 (2020).

[3] Giacomazzi, S. & Cochet, N. Environmental impact of diuron transformation: a review. *Chemosphere* **56**, 1021–1032 (2004).

[4] Peillex, C. & Pelletier, M. The impact and toxicity of glyphosate and glyphosate-based herbicides on health and immunity. *J. Immunotoxic.* **17**, 163–174 (2020).

[5] S. Lagergren. Zur theorie der sogenannten adsorption gelöster stoffe, Kunglinga Svenska Vetenskapsakademiens. *Handlingar* **24**, 1–39 (1898).

[6] Ho, Y. S. & McKay, G. Pseudo-second order model for sorption processes. *Process Biochem*. **34**, 451–465 (1999).

[7] Ouyang, J., Zhou, L., Liu, Z., Heng, J. Y. Y. & Chen, W. Biomass-derived activated carbons for the removal of pharmaceutical mircopollutants from wastewater: A review. *Sep. Pur. Tech.* **253**, 117536 (2020).

[8] Foo, K. Y. & Hameed, B. H. Insights into the modeling of adsorption isotherm systems. *Chem. Eng.* J. **156**, 2–10 (2009).

[9] Szewczuk-Karpisz, K. *et al.* Impact of Sinorhizobium meliloti Exopolysaccharide on Adsorption and Aggregation in the Copper(II) Ions/Supporting Electrolyte/Kaolinite System. *Materials* **14**, 1950 (2021).

[10] Tomczyk, A., Kubaczyński, A. & Szewczuk-Karpisz, K. Assessment of agricultural waste biochars for remediation of degraded water-soil environment: Dissolved organic carbon release and immobilization of impurities in one- or two-adsorbate systems. *Waste Management* **155**, 87–98 (2023).

[11] Kończyk, J., Kluziak, K. & Kołodyńska, D. Adsorption of vanadium (V) ions from the aqueous solutions on different biomass-derived biochars. *J. Environ. Manag.* **313**, 114958 (2022).

[12] Razzaq, Z. *et al.* Removal of As(V) and Cr(VI) with Low-Cost Novel Virgin and Iron-Impregnated Banana Peduncle-Activated Carbons. *ACS Omega* **8**, 2098–2111 (2023).

[13] Tuna, A. Ö. A., Özdemir, E., Şimşek, E. B. & Beker, U. Removal of As(V) from aqueous solution by activated carbon-based hybrid adsorbents: Impact of experimental conditions. *Chem. Eng. J.* **223**, 116–128 (2013).

[14] An experimental design approach for modeling As(V) adsorption from aqueous solution by activated carbon. *Water Sci. Tech*. **71**, 203–210 (2014).

[15] López-Toyos, L., Rodríguez, E., García, R., Martínez-Tarazona, M. R. & López-Antón, M. A. Sorption of Selenium(IV) and Selenium(VI) onto Iron Oxide/Hydroxide-Based Carbon Materials: Activated Carbon and Carbon Foam. *Water* **15**, 3499 (2023).

[16] Jegadeesan, G. B., Mondal, K. & Lalvani, S. B. Adsorption of Se (IV) and Se (VI) Using Copper-Impregnated Activated Carbon and Fly Ash-Extracted Char Carbon. *Water Air & Soil Pollution* **226**, (2015).

[17] Strong, O. K. L., France, H. E., Scotland, K., Wright, K. & Vreugdenhil, A. J. Selenite Adsorption and Reduction via Iron(II) Impregnated Activated Carbon Produced from the Phosphoric Acid Activation of Construction Waste Wood. *Arch. Environ. Contam. Toxicol*. **85**, 485–497 (2023).

[18] Zhang, N., Lin, L.-S. & Gang, D. Adsorptive selenite removal from water using iron-coated GAC adsorbents. *Water Res.* **42**, 3809–3816 (2008).

[19] Madhavarao, M., Ramesh, A., Purnachandrarao, G. & Seshaiah, K. Removal of copper and cadmium from the aqueous solutions by activated carbon derived from Ceiba pentandra hulls. *J. Hazard. Mater.* **129**, 123–129 (2005).

[20] Rao, M. M., Rao, G. P. C., Seshaiah, K., Choudary, N. V. & Wang, M. C. Activated carbon from Ceiba pentandra hulls, an agricultural waste, as an adsorbent in the removal of lead and zinc from aqueous solutions. *Waste Manag.* **28**, 849–858 (2007).

[21] El Malti, W. *et al.* Comparative study of the elimination of copper, cadmium, and methylene blue from water by adsorption on the citrus Sinensis peel and its activated carbon. *RSC Advances* **12**, 10186–10197 (2022).

[22] Neisan, R. S., Saady, N. M. C., Bazan, C., Zendehboudi, S. & Albayati, T. M. Adsorption of copper from water using TiO2-modified activated carbon derived from orange peels and date seeds: Response surface methodology optimization. *Heliyon* **9** (2023).

[23] Darweesh, M. A. *et al.* Adsorption isotherm, kinetic, and optimization studies for copper (II) removal from aqueous solutions by banana leaves and derived activated carbon. *S. Afr. J. Chem. Eng.* **40**, 10–20 (2022).

[24] Obregón-Valencia, D. & Del Rosario Sun-Kou, M. Comparative cadmium adsorption study on activated carbon prepared from aguaje (Mauritia flexuosa) and olive fruit stones (Olea europaea L.). *J. Environ. Chem. Eng.* **2**, 2280–2288 (2014).

[25] Pap, S. *et al.* Utilization of fruit processing industry waste as green activated carbon for the treatment of heavy metals and chlorophenols contaminated water. *J. Clean. Prod*. **162**, 958–972 (2017).

[26] El Malti, W. *et al.* Comparative study of the elimination of copper, cadmium, and methylene blue from water by adsorption on the citrus Sinensis peel and its activated carbon. *RSC Advances* **12**, 10186–10197 (2022).

[27] Szewczuk-Karpisz, K., Bajda, T., Tomczyk, A., Kuśmierz, M. & Komaniecka, I. Immobilization mechanism of Cd2+/HCrO4-/CrO42- ions and carboxin on montmorillonite modified with Rhizobium leguminosarum bv. trifolii exopolysaccharide. *J. Hazard. Mater.* **428**, 128228 (2022).

[28] Szewczuk-Karpisz, K., Nowicki, P., Sokołowska, Z. & Pietrzak, R. Hay-based activated biochars obtained using two different heating methods as effective low-cost sorbents: Solid surface characteristics, adsorptive properties and aggregation in the mixed Cu(II)/PAM system. *Chemosphere* **250**, 126312 (2020).
